# Luminescent ion pairs with tunable emission colors for light-emitting devices and electrochromic switches†
†Electronic supplementary information (ESI) available: Details of NMR and MS spectra. See DOI: 10.1039/c6sc02837c



**DOI:** 10.1039/c6sc02837c

**Published:** 2016-08-15

**Authors:** Song Guo, Tianci Huang, Shujuan Liu, Kenneth Yin Zhang, Huiran Yang, Jianmei Han, Qiang Zhao, Wei Huang

**Affiliations:** a Key Laboratory for Organic Electronics and Information Displays and Institute of Advanced Materials (IAM) , Jiangsu National Synergetic Innovation Center for Advanced Materials (SICAM) , Nanjing University of Posts and Telecommunications (NUPT) , Nanjing 210023 , P. R. China . Email: iamqzhao@njupt.edu.cn; b Key Laboratory of Flexible Electronics (KLOFE) and Institute of Advanced Materials (IAM) , Jiangsu National Synergetic Innovation Center for Advanced Materials (SICAM) , Nanjing Tech University (NanjingTech) , Nanjing 211816 , P. R. China . Email: wei-huang@njtech.edu.cn

## Abstract

Most recently, stimuli-responsive luminescent materials have attracted increasing interest because they can exhibit tunable emissive properties which are sensitive to external physical stimuli, such as light, temperature, force, and electric field. Among these stimuli, electric field is an important external stimulus. However, examples of electrochromic luminescent materials that exhibit emission color change induced by an electric field are limited. Herein, we have proposed a new strategy to develop electrochromic luminescent materials based on luminescent ion pairs. Six tunable emissive ion pairs (**IP1–IP6**) based on iridium(iii) complexes have been designed and synthesized. The emission spectra of ion pairs (IPs) show concentration dependence and the energy transfer process is very efficient between positive and negative ions. Interestingly, **IP6** displayed white emission at a certain concentration in solution or solid state. Thus, in this contribution, UV-chip (365 nm) excited light-emitting diodes showing orange, light yellow and white emission colors were successfully fabricated. Furthermore, IPs displayed tunable and reversible electrochromic luminescence. For example, upon applying a voltage of 3 V onto the electrodes, the emission color of the solution of **IP1** near the anode or cathode changed from yellow to red or green, respectively. Color tunable electrochromic luminescence has also been realized by using other IPs. Finally, a solid-film electrochromic switch device with a sandwiched structure using **IP1** has been fabricated successfully, which exhibited fast and reversible emission color change.

## Abstract 2

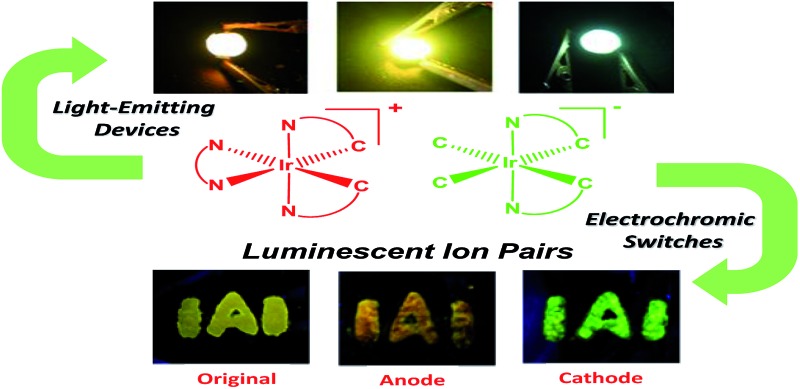
A class of luminescent ion pairs with tunable emission colors was designed and synthesized for light-emitting devices and electrochromic switches.

## Introduction

**Fig. 1:**
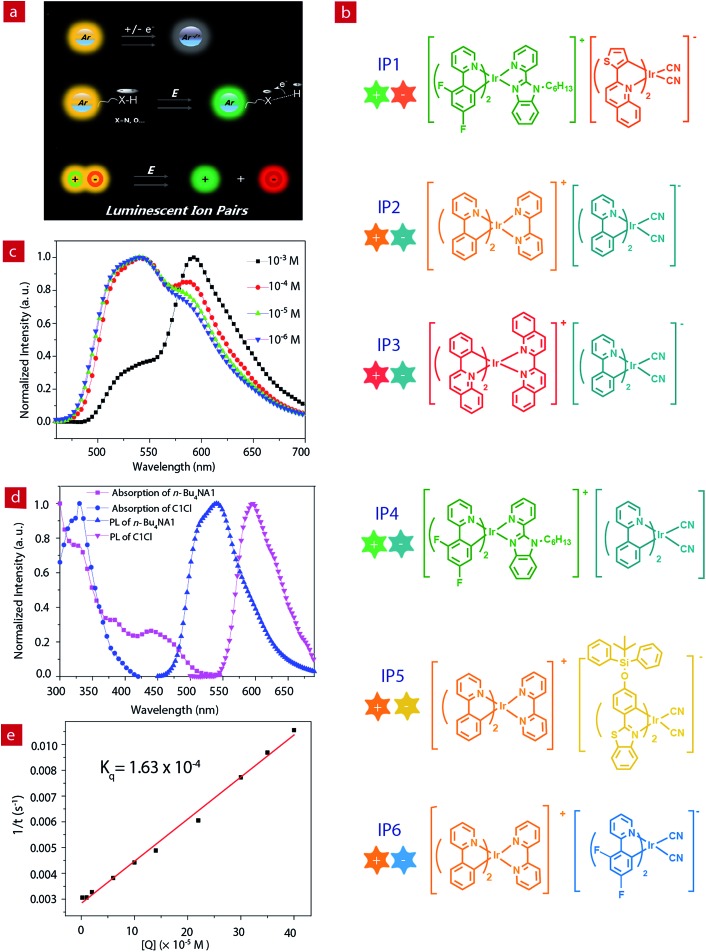
(a) The design principles of electrochromic luminescent materials. (b) The structures of six ion pairs. (c) Luminescence spectra of **IP1** at different concentrations in acetonitrile. (d) Absorption and luminescence spectra of **C1Cl** and ***n*-Bu_4_NA1** (10^–5^ M). (e) Stern–Volmer plot of the quenching study between **C1Cl** and ***n*-Bu_4_NA1** and the numerical fitting of *K*_q_.

Luminescent materials have attracted considerable attention because of their wide applications in displays, data recording and storage, chemical sensing, bioimaging, *etc.* ^1–9^ Emissive materials with tunable luminescence colors are of great significance for their optical applications. Up to now, conventional methods for tuning the photophysical properties of luminescent materials are mainly limited to the modification of the chemical structures, such as the change of π-conjugation skeletons or the introduction of functional groups.^10–21^ However, this method often requires complicated synthetic processes and tedious purification procedures. Stimuli-responsive luminescent materials have attracted increasing interest, because they can exhibit tunable emissive properties which are sensitive to external physical stimuli, such as light, temperature, force, electric field, *etc.* ^22–28^ Among these stimuli, electric field is an important external stimulus, which can be conveniently combined with the current semiconductor technology in electronic devices.^29–33^ Recently, electrochromic luminescent materials that exhibited emission color changing induced by an electric field have been reported (Fig. 1a). For example, Chidichimo and co-workers have presented π-conjugated ionic liquid crystals in which the direct electrochemical reduction leads to a reversible electrochromic luminescence (ECL) response.^34^ Beneduci and co-workers have shown an ECL-active polymer gel based on the conversion of the redox states, in which the emission properties of the polymer depend on the fluorophore contents and the voltages applied.^35^ We have also reported a series of phosphorescent iridium(iii) complexes containing a protonating functional group, which exhibited evident emission color change induced by an electric field.^36–38^ However, special functional groups, such as redox active or protonating groups, in these electrochromic luminescent materials may reduce their stability, especially toward redox reagents and pH values. Therefore, it is urgent to explore a new generation of electrochromic luminescent materials with controllable emission colour change.

Ion pairs (IPs) consist of an anionic and a cationic component which are bonded together with electrostatic and van der Waals interactions. Employment of emissive anionic and cationic components yields luminescent IPs showing a mixed luminescence color.^39–43^ Compared to single luminophores, IPs containing two luminescent chromophores exhibit richer excited states, which results from potential energy transfer between the two luminophores. In addition, IPs exhibit a “single-component” characteristic and can avoid phase separation in the solid state, which usually occurs in a physical mixture of two luminophores.

In this work, we aim to develop luminescent IPs with tunable emissive colors. Owing to the advantageous photophysical properties of phosphorescent iridium(iii) complexes, such as high luminescence quantum yields, long emission lifetimes, large Stokes shifts, high photostability, and photophysical properties sensitive to the microenvironment, a series of positively- and negatively-charged iridium(iii) complexes have been chosen as the cationic and anionic components of luminescent IPs (**IP1–IP6**, Fig. 1b), respectively.^44–53^ Upon photoexcitation, these IPs exhibited a mixed luminescence from both the cation and the anion. The emission spectra of **IP1–IP6** show concentration dependence. In particular, **IP6** displayed white emission at a suitable concentration in solution or solid state. Thus, in this contribution, UV-chip (365 nm) excited light-emitting diodes (LEDs) showing orange, light yellow and white emission colors were successfully fabricated using **IP6** as the emitter. In addition, interesting and tunable electrochromic luminescence has been observed in solution and quasi-solid devices, and the electrochromic mechanism of these IPs has been investigated.

## Results and discussion

### Synthesis and characterizations

**Scheme 1:**
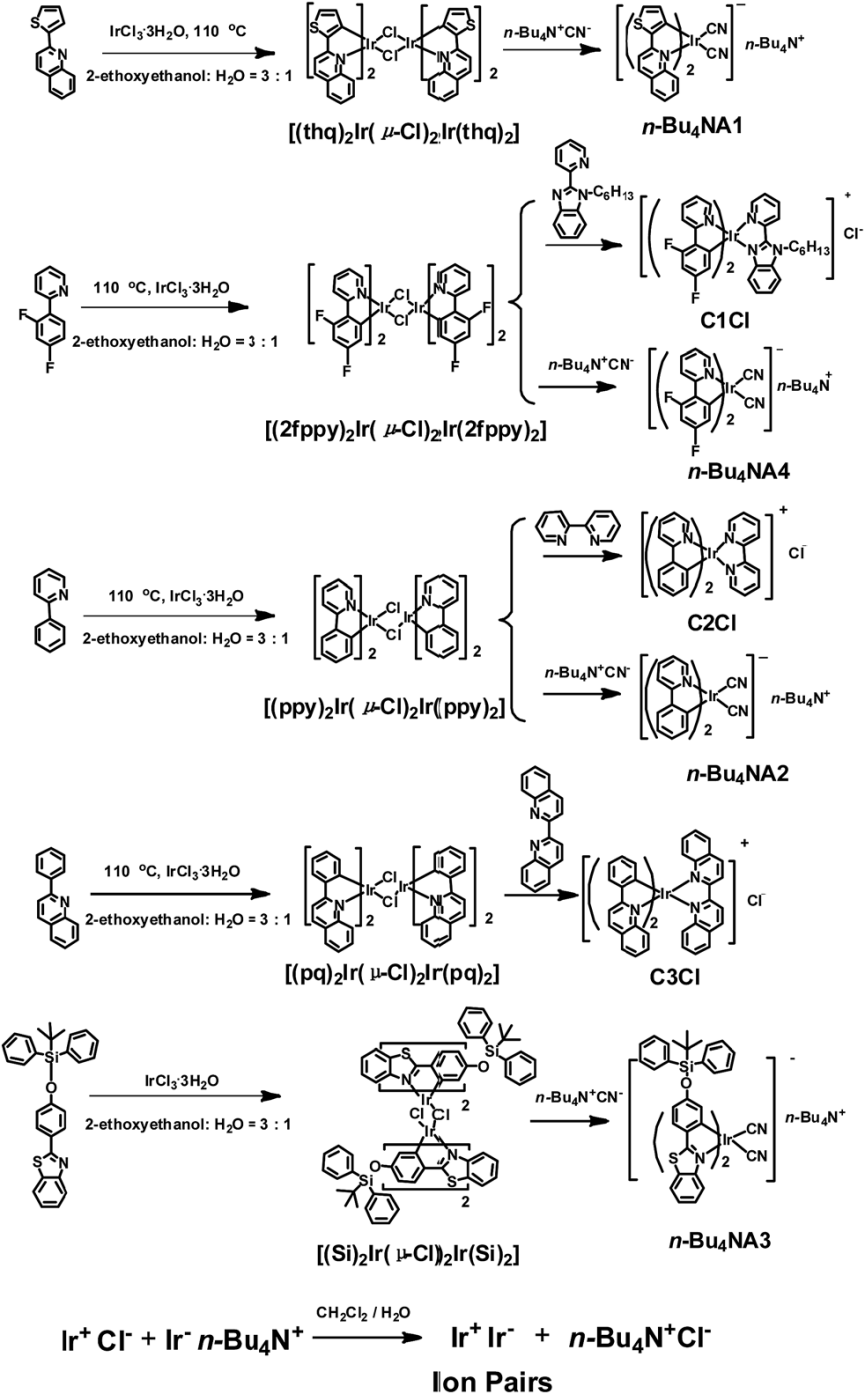
The synthetic routes of positive and negative Ir(iii) complexes and ion pairs.

The photophysical properties of iridium(iii) complexes are closely related to their ligands. Hence, to obtain IPs showing different luminescent colors, various cationic and anionic complexes ((C^N)_2_Ir(N^N)^+^Cl^–^ and (C^N)_2_Ir(CN)_2_-*n*-Bu_4_N^+^) with different HC^N ligands (HC^N = 2-phenylpyridine, 2-phenylquinoline, 2-(2,4-disfluorophenyl)pyridine, 2-thiophenylquinoline, *N*-hexyl-2-(2-pyridyl)benzimidazole, 2-(4-((*tert*-butyldiphenylsilyl)oxy)phenyl)-phenylbenzothiazole; N^N = 2,2′-biquinoline, 2,2′-dipyridine, cyanide) were selected to prepare the luminescent IPs and the emission colors of these complexes cover the entire visible region. The luminescent IPs were synthesized in two steps. Firstly, three cationic (**C1Cl–C3Cl**) and four anionic complexes (***n*-Bu_4_NA1–*n*-Bu_4_NA4**) were synthesized by refluxing biscyclometalated iridium(iii) dichloro-bridged dimers in the presence of an excess of auxiliary N^N or CN^–^ ligands. In the second step, six IPs without small counterions, **IP1–IP6** (**C1A1**, **C2A2**, **C3A2**, **C1A2**, **C2A3** and **C2A4**), were obtained by the metathesis reactions (Schemes 1 and S1†).^43,54–58^

All the complexes and IPs have been fully characterized by ^1^H nuclear magnetic resonance (NMR), ^13^C{^1^H} NMR, ^19^F{^1^H} NMR, matrix-assisted laser desorption ionization time-of-flight mass spectrometry (MALDI-TOF MS), electrospray ionization mass spectrometry (ESI-MS) (see ESI†), UV-Vis absorption spectrometry and steady-state photoluminescence (PL) spectrometry. In ^1^H NMR spectra, the anionic complexes exhibited four peaks at 3.06, 1.58, 1.34 and 0.96 ppm in CD_3_CN, which can be assigned to the tetrabutylammonium cation. These signals were absent in the ^1^H NMR spectra of IPs, indicating the successful metathesis reaction and the quantitative synthesis of the IPs starting from equal molar quantity of the two luminescent components in dichloromethane.

### Photophysical properties

The photoluminescence properties of the complexes were investigated. The PL spectra of the cationic (**C1Cl–C3Cl**) and anionic (***n*-Bu_4_NA1–*n*-Bu_4_NA4**) complexes in acetonitrile solution are presented in Fig. 1d and Fig. S3,† peaking at 524, 591, 638, 550, 595, 475 and 523 nm, respectively. For the IPs, their emissions are contributed to by both cations and anions. Interestingly, the PL spectra of the IPs exhibit concentration dependence (Fig. S1†). Taking **IP1** as an example (Fig. 1c), at a relatively low concentration of 10^–6^ M, the emission peak of **IP1** is around 524 nm, which originated from **C1**, because the quantum yield of **C1** (21%) is much higher than that of **A1** (17%). As the concentration of solution increases, the green emission from **C1** was quenched by the anionic complex **A1**, due to energy transfer from **C1** to **A1**. When the concentration of **IP1** was increased to 10^–3^ M, the PL spectrum is dominated by the emission from the anionic component. The emission spectra of **IP1–IP6** were also measured in various solvents (THF, ethyl acetate, CHCl_3_, CH_2_Cl_2_, CH_3_OH), and there was no evident change in different solvents at the same concentration of IPs as shown in Fig. S2.†

The energy transfer process between the two components of **IP1** was investigated by Stern–Volmer quenching analysis (Fig. 1d and e). The quenching study was based on a bimolecular quenching model, *τ*_0_/*τ* = 1 + *K*_q_ *τ*_0_[Q], where *τ* and *τ*_0_ are the emission lifetimes of ***n*-Bu_4_NA1** with and without the quencher **C1Cl**, *K*_q_ is the experimental quenching rate constant and [Q] is the molar concentration of the quencher. The lifetimes of ***n*-Bu_4_NA1** in acetonitrile solution with various amounts of the quencher, **C1Cl**, were measured. The concentration of ***n*-Bu_4_NA1** was kept at 1.0 × 10^–5^ M, while that of **C1Cl** varied from 0 to 4.0 × 10^–4^ M. The Stern–Volmer plot of the mixture of the two complexes in solution revealed a good linear relationship between *τ*_0_/*τ* and [Q]. The calculation yields a *K*_q_ value of 1.63 × 10^–4^ M^–1^ s^–1^. This quenching effect can be attributed to the intermolecular triplet–triplet energy transfer.^43,46^ The results indicate that the energy transfer quenching process is very efficient between the two ionic complexes.

### White LED devices

**Fig. 2:**
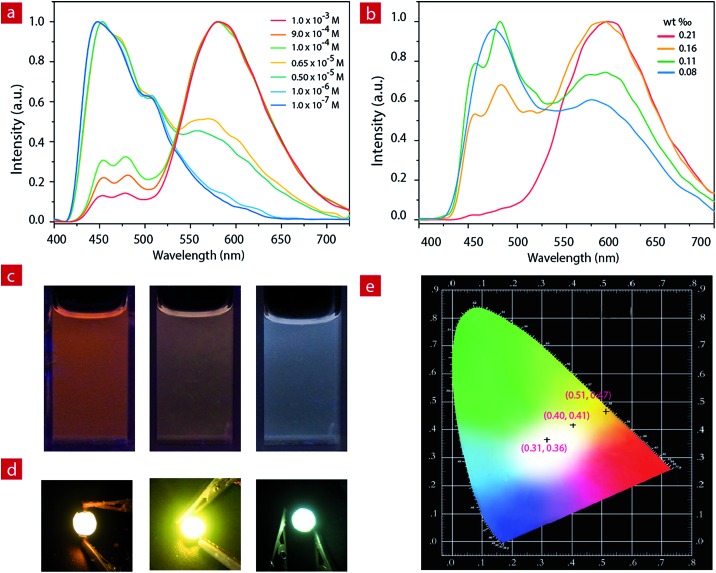
(a) The luminescence spectra of **IP6** at different concentrations in acetonitrile. (b) The solid state luminescence spectra of **IP6** doped into a polyethylene–polypropylene glycol polymer with different weight ratios. (c) Photographs of **IP6** at different concentrations (left, 1.0 × 10^–3^ M; middle, 1.0 × 10^–4^ M; right, 6.5 × 10^–6^ M) in acetonitrile under excitation at 365 nm. (d) The pictures of the orange (left, 1.7 mg in 800 mg), light-yellow (middle, 0.9 mg in 800 mg) and white (right, 0.7 mg in 800 mg) LEDs operated at 3.5 V. (e) CIE 1931 chromaticity diagram of the obtained orange, light-yellow and white light from the fabricated LED devices.

For **IP6**, the two luminescent components peak at 475 nm and 580 nm. At a concentration of 1.0 × 10^–3^ M, the ion pair shows orange emission. With the decrease of concentration in CH_3_CN, the greenish-blue emission increases gradually (Fig. 2a and c). When the concentration decreases to 6.5 × 10^–6^ M, the solution exhibits white emission with the Commission Internationale de L'Eclairage (CIE) of (0.28, 0.30) and a quantum efficiency of 0.22. As the concentration continues to decrease, the solution shows weak blue emission.

**IP6** was doped into a polyethylene–polypropylene glycol polymer to investigate the photophysical properties of the solid film. At different doping concentrations, the film showed different emission colors (Fig. 2b, and S4†). At a concentration of about 1.9 mg **IP6** in 800 mg polymer, the solid film exhibited orange emission. With the decrease of the doping concentration, the solid film showed light yellow emission gradually. Finally, when the concentration decreased to about 0.7 mg **IP6** in 800 mg polymer, the solid film showed white emission with a quantum efficiency of 0.16. **IP6**, therefore, is a potential candidate material for white LED (WLED).

Compared to the widely used inorganic phosphors, organic emitters have a greater advantage due to their large absorption cross section, which can reduce the phosphor consumption and cut down the costs.^59^ Here, to fabricate the near-UV excited LED, powdered organic emitter **IP6** was dispersed in a polyethylene–polypropylene glycol polymer and then coated onto the surface of commercially available 365 nm UV LED chips. Bright orange, light yellow and white emission were obtained when polyethylene–polypropylene glycol polymers doped with different concentrations (1.9 mg/800 mg, 0.9 mg/800 mg and 0.7 mg/800 mg) of **IP6** were coated onto the UV chip as emitters. We can clearly see the bright orange, light yellow and white emissions from the prepared devices with CIE coordinate values of (0.51, 0.47), (0.40, 0.41) and (0.31, 0.36), respectively (Fig. 2d).

### Electrochromic switches

**Fig. 3:**
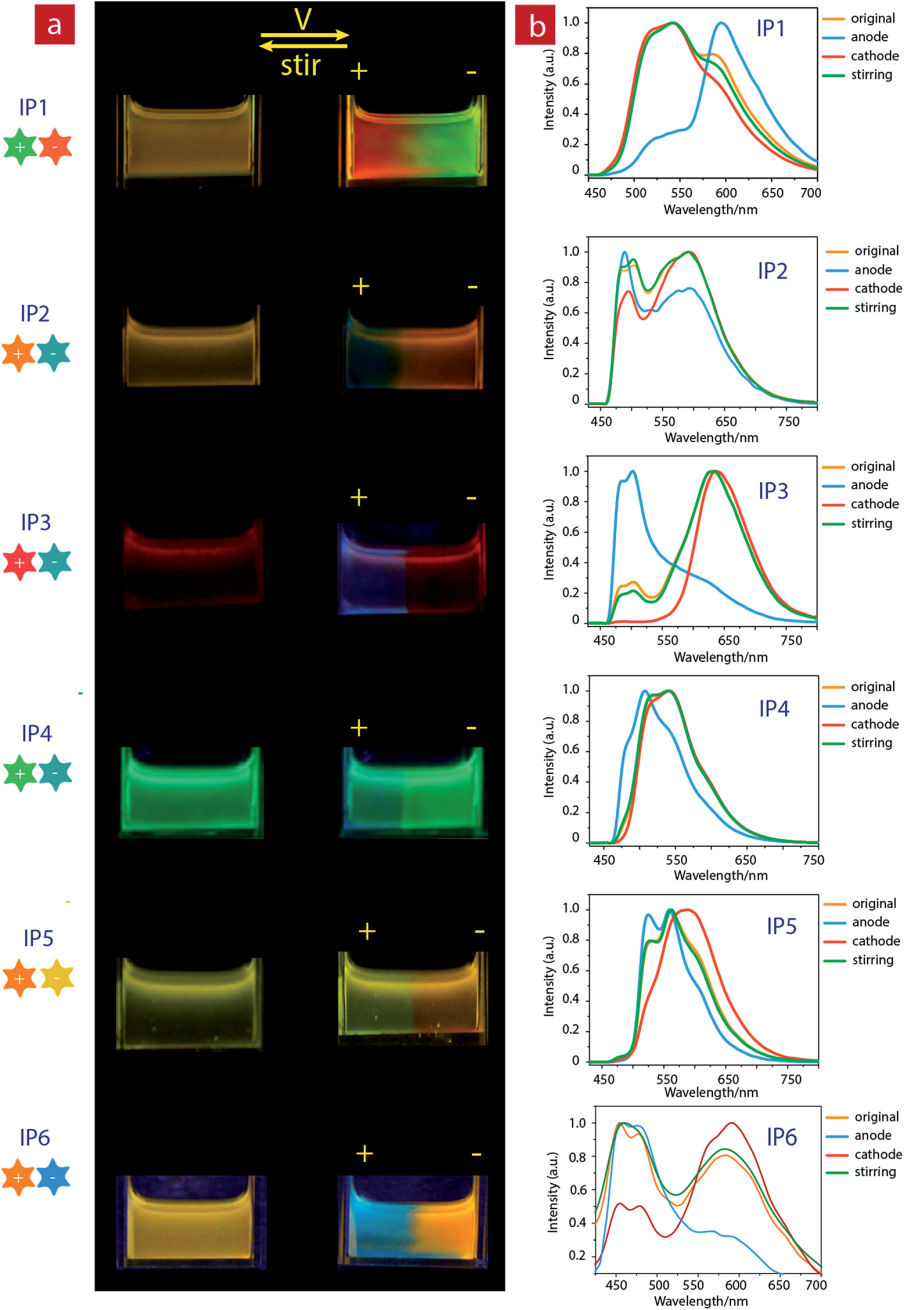
(a) Photographs of ion pairs (10^–5^ M) in acetonitrile before (left) and after (right) applying a voltage of 3 V, followed by stirring and reversing the voltage under excitation at 365 nm. (b) Luminescence spectra of ion pairs (10^–5^ M) in acetonitrile before and after applying a voltage at the cathode and anode, respectively.

Considering that IPs consist of luminescent ions with opposite charges, the electric field is anticipated to regulate the luminescent behavior. In order to demonstrate this behavior, **IP1** was used to perform the electrochromic luminescence experiment as illustrated in Fig. 3a. A concentration of 10^–5^ M of the ion pair was chosen, because the emission intensity of the two peaks of **IP1** was almost equal at this concentration. Two platinum electrodes were immersed in a CH_3_CN solution of **IP1** with a distance of 20 mm between each other. Before applying a voltage, the solution of **IP1** exhibited yellow emission. Subsequently, upon applying a voltage of 3 V onto the electrodes, the emission color of the solution near the anode changed from yellow to red within 30 s, which is in accordance with its cationic component (**C1**). Meanwhile, the yellow emission near the cathode changed to green, which is same as that of the anionic component (**A1**). Such a change of emission color gradually extends to the middle of the two electrodes, appearing as a clear boundary.

Then once the voltage was removed, the original yellow emission was recovered after stirring the solution and the electrochromic luminescence reappeared when the stirring was stopped. This observation indicates that **IP1** shows an excellent reversibility for electrochromic luminescence. To explore the mechanism of this phenomenon, the emission spectra of **IP1** near the anode and cathode after electrical stimuli are measured and shown in Fig. 3b. It was found that the emission spectra of the solution near the anode and cathode are almost the same as those of the anionic (***n*-Bu_4_NA1**) and cationic iridium(iii) complex (**C1Cl**), respectively. Therefore, we believe that the anionic component directionally moves to the anode and the cationic one shifts to the cathode because the electrostatic interaction was broken under an electric field, resulting in the emission color changes near the two electrodes.

**Fig. 4:**
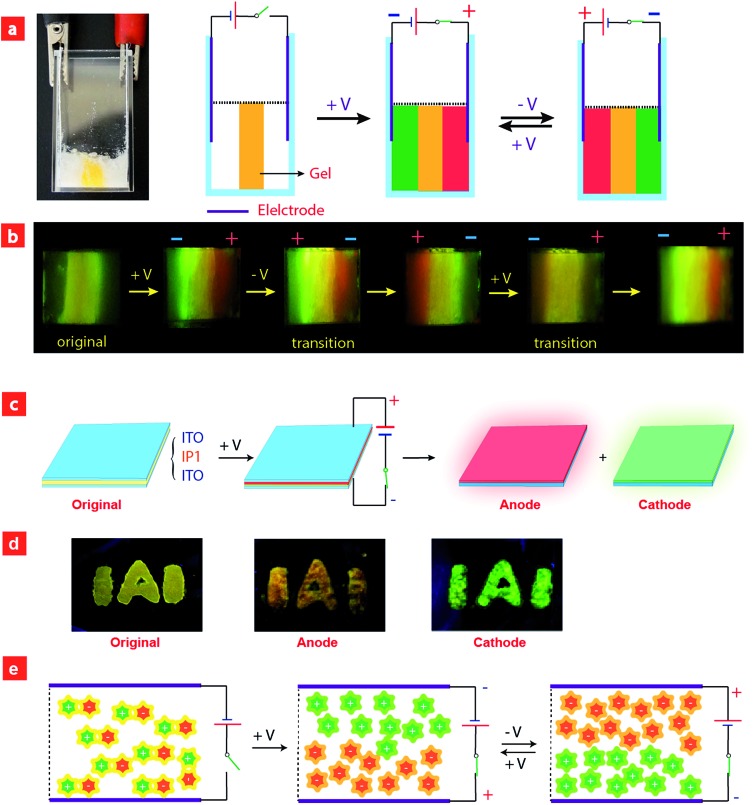
(a and c) The schematic diagram of the setup for electrochromic luminescence experiments. (b and d) Photographs of electrochromic luminescence experiments using quasi-solid film doped with **IP1**. (e) Schematic illustration of ion migration under electric stimuli.

To have a better understanding of these observations, a schematic model was proposed in Fig. 4e. Before applying a voltage, the IPs were disordered and uniformly distributed in the solution, and the interaction between cationic and anionic components is mainly through electrostatic force, which can be broken upon applying a voltage, leading to migration of cationic and anionic components to the cathode and anode, respectively. After removing the voltage and stirring the solution, the two components held together again. This mechanism was confirmed by the NMR experiments. Because of the asymmetry of the ancillary ligand in **C1Cl**, the two C^N ligands are non-chemically equivalent. There are two peaks attributed to the proton in the b-position with each integral of 1 (Fig. S5†). For ***n*-Bu_4_NA1**, the two C^N ligands are chemically equivalent owing to the symmetry of the ancillary ligands CN^–^ in the structure. Eight peaks from the C^N ligand could be observed in the NMR spectrum. The integral of the proton in the a-position is 2. In the original NMR spectrum of **IP1**, the integral ratio of protons at the a-position to the b-position is 2 : 1, indicating that the anion and cation are equal. After applying a voltage, the integral ratio in the NMR spectrum of the solution near the cathode changed to 0.56 : 1.00, while that near the anode changed to 2.00 : 0.82. These results demonstrated the migration of anions and cations to anode and cathode, respectively, under the electric field.

To prove that the formation of IPs is necessary for realizing this electrochromism phenomenon, a control experiment using a mixed solution of ***n*-Bu_4_NA1** and **C1Cl** (molar ratio 1 : 1) was carried out. Upon applying a voltage of 3 V to the electrodes for about 2 minutes, no emission color change was observed (Fig. S6†), which may be because the small counterions **Cl^–^** and ***n*-Bu_4_N^+^** moved to the anode and cathode preferentially compared to the bulky ionic iridium(iii) complexes. Hence, our design strategy based on luminescent IPs is effective to realize the electrochromic luminescence.

Furthermore, color-tunable electrochromic luminescence has been realized by using other IPs (**IP2–IP6**, Fig. 3). For example, the emission color of **IP2** can be changed from yellow to blue at the anode and orange at the cathode. For **IP3**, the emission color at the anode changed from red to blue, while that near the cathode remained red because of efficient energy transfer. For **IP4**, the emission color changed from green to blue at the anode, and remained green near the cathode. For **IP5**, the emission color at the cathode changed from yellow to orange, and that near the anode remained yellow. For **IP6**, the emission color at the cathode changed from orange to blue-green, and that near the anode remained orange.

Based on the above results, we further realized the electrochromic luminescence in a quasi-solid state. A device with a simple sandwiched structure, in which a quasi-solid film was coated between two Pt electrodes, has been fabricated. The yellow-emitting quasi-solid film was prepared by mixing ion pair **IP1** with DMSO and SiO_2_ particles (300–400 mesh). The IPs are able to freely migrate within the inorganic network channels of the SiO_2_ skeleton and the phosphorescence properties of IPs would not be affected by the presence of SiO_2_ particles. Under a voltage of 3 V, the luminescent color near the anode changed from yellow to red and that near the cathode was altered to green, which is consistent with the electrochromic luminescence observed in solution. When reversing the direction of the electric field, the emission colors near the two electrodes exchanged within 20 minutes (Fig. S7†).

Next, we carried out another experiment to further confirm that the mechanism of electrochromic luminescence is due to the migrations of the ions under an electric field, as shown in Fig. 4a and b. At first, a quasi-solid gel was prepared by adding several drops of DMSO into SiO_2_ particles and then coated between two Pt electrodes. **IP1** was doped into the middle of the gel, which showed a yellow emission. Upon applying a voltage of 3 V for 10 minutes, green emission color appeared and gradually spread to the cathode. Meanwhile, the red emission color spread to the anode gradually. Next, the direction of the electric field was exchanged, and the emission colors near the two edges were switched. This phenomenon can be repeated. The above results demonstrated that the electrochromic luminescence is induced by the migration of the ions.

Finally, a simple sandwiched structure device, in which a quasi-solid film (about 2 mm in thickness) doped with **IP1** was coated between two ITO electrodes, has been fabricated as shown in Fig. 4c and d. At first, a letter pattern of “**IAI**” was coated onto the ITO electrodes. The color of the pattern was orange. Then a voltage of 3 V was applied to the ITO electrodes for about 3–4 seconds. The emission color of the film near the anode changed to red immediately. Meanwhile, a green color appeared near the cathode. This phenomenon can be repeated very well.

## Conclusions

In summary, we have developed a class of tunable emissive materials based on luminescent ion-paired iridium(iii) complexes. The photophysical properties of the IPs have been studied in detail. Orange, light yellow and white light-emitting devices were successfully fabricated by coating the polymer films doped with **IP6** on commercially available ultraviolet LEDs. Furthermore, a new strategy for the design of electric-responsive materials which display tunable and reversible electrochromic luminescence was presented. Changing the moiety of cationic or anionic iridium(iii) complexes moiety can be used to tune the electrochromic luminescence colors of these ion pairs. To demonstrate the potential practical applications, a solid-film electrochromic switch device using IPs, which showed a fast and reversible emission color change, has been fabricated successfully. These results showed that electrochromic luminescent materials based on ion pairs will be promising candidates for applications in optoelectronic fields.

## Experimental

### General

Unless otherwise stated, all starting materials and reagents were purchased from commercial suppliers and were used without further purification. Silica particles (Aerosil 200, 12 nm) were purchased from Degussa Company (Evonik Industries AG, Rellinghauser Strabe 1-11, 45128 Essen, Germany). CH_2_Cl_2_, CH_3_CN and CH_3_OH were dried under reflux over CaH_2_ or sodium for several hours at 70–75 °C, distilled at these conditions and used fresh. NMR spectra were recorded on a Bruker Ultra Shield Plus 400 MHz NMR instrument (^1^H: 400 MHz, ^13^C{^1^H}: 100 MHz, ^19^F{^1^H}: 377 MHz). Chemical shifts (ppm) were reported relative to tetramethylsilane (TMS). Mass spectra were obtained on a Bruker autoflex MALDI-TOF/TOF mass spectrometer and a ESI-MS (LCQ Fleet, Thermo Fisher Scientific). UV-Vis absorption spectra were recorded on a UV-1700 Shimadzu UV-Vis spectrophotometer. The photoluminescence spectra and emission lifetimes were recorded on an Edinburgh FL920 spectrofluorometer system.

### Synthesis of **1**

A mixture of 2-bromopyridine (1.03 g, 6.52 mmol), 2,4-difluorophenylboronic acid (0.94 g, 5.95 mmol), Pd(PPh_3_)_4_ (0.21 g, 0.18 mmol) and potassium carbonate (1.0 g) in toluene (30.0 mL), H_2_O (10.0 mL) and EtOH (10.0 mL) was stirred for 24 h at 85 °C under nitrogen. The reaction mixture was cooled down to room temperature, then 50.0 mL H_2_O was added, and the solution was extracted with dichloromethane. The organic layer was dried over Na_2_SO_4_, and the solvents were removed *in vacuo*. The crude material was purified by column chromatography over silica gel (PE → PE/EA, 75 : 1), yielding the yellow oil. Yield: 1.08 g (95.0%).

^1^H NMR (400 MHz, DMSO-*d*_6_): *δ* = 8.70 (ddd, *J*_1_ = 0.8 Hz, *J*_2_ = 1.6 Hz, *J*_3_ = 4.8 Hz, 1H); 7.98 (dt, *J*_1_ = 6.8 Hz, *J*_2_ = 9.2 Hz, 1H); 7.88 (dt, *J*_1_ = 1.6 Hz, *J*_2_ = 7.6 Hz, 1H); 7.74 (m, 1H); 7.38 (ddd, *J*_1_ = 1.2 Hz, *J*_2_ = 4.8 Hz, *J*_3_ = 7.6 Hz, 1H); 7.35 (ddd, *J*_1_ = 2.4 Hz, *J*_2_ = 9.2 Hz, *J*_3_ = 11.6 Hz, 1H); 7.20 (ddt, *J*_1_ = 0.8 Hz, *J*_2_ = 2.4 Hz, *J*_3_ = 8.8 Hz, 1H). ^13^C{^1^H} NMR (100 MHz, DMSO): *δ* = 162.57 (dd, ^3^ *J*_C–F_ = 12.3 Hz, ^1^ *J*_C–F_ = 247.1 Hz); 159.90 (dd, ^3^ *J*_C–F_ = 12.3 Hz, ^1^ *J*_C–F_ = 249.6 Hz); 151.64 (d, ^3^ *J*_C–F_ = 2.5 Hz), 149.81; 136.94; 132.23 (dd, ^3^ *J*_C–F_ = 4.5 Hz, ^3^ *J*_C–F_ = 9.8 Hz); 123.93 (d, ^3^ *J*_C–F_ = 8.6 Hz); 123.70 (d, ^2^ *J*_C–F_ = 11.8 Hz); 122.90; 112.05 (d, ^2^ *J*_C–F_ = 21.1 Hz); 104.56 (t, ^2^ *J*_C–F_ = 27.0 Hz). ^19^F{^1^H} NMR (376.5 MHz, DMSO) *δ* = –109.35 (d, *J* = 8.28 Hz); –112.826 (d, *J* = 8.28 Hz). ESI-MS (*m*/*z*): calcd for C_11_H_7_F_2_N, 191.05; found, 191.12.

### Synthesis of **2**

A mixture of 2-(2-pyridyl)benzimidazole (200.0 mg, 2.5 mmol), *n*-hexane (175.0 mg, 2.6 mmol) and potassium hydroxide (60.0 mg, 2.5 mmol) in DMF (5.0 mL) was heated at room temperature under nitrogen atmosphere for 4 h, and then extracted with CH_2_Cl_2_ and water. The crude product was purified through column chromatography on silica gel (PE → PE/EA, 30 : 1) to yield pure product as a colorless oil. Yield: 0.46 mg (61.0%).

^1^H NMR (400 MHz, CDCl_3_): *δ* = 8.67 (d, *J* = 4.0 Hz, 1H); 8.38 (d, *J* = 8.0 Hz, 1H); 7.85–7.81 (m, 2H); 7.44 (dd, *J*_1_ = 6.7 Hz, *J*_2_ = 2.0 Hz, 1H); 7.35–7.27 (m, 3H); 4.81 (t, *J* = 8.0 Hz, 3H); 1.86 (dt, *J* = 15.0 Hz, 7.6 Hz, 2H); 1.36–1.24 (m, 6H); 0.85 (t, *J* = 7.1 Hz, 3H). ^13^C{^1^H} NMR (100 MHz, CDCl_3_, 298 K): *δ* = 150.92; 150.08; 148.76; 142.81; 136.89; 136.77; 124.82; 123.80; 123.31; 122.60; 120.21; 110.37; 45.58; 31.47; 30.13; 26.62; 22.64; 14.12. ESI-MS (*m*/*z*): calcd for C_18_H_21_N_3_, 279.17; found, 279.28.

### Synthesis of **3**

A mixture of 2-aminothiophenol (4.0 g) and 4-hydroxybenzaldehyde (4.12 mL) in DMF (10 mL) was heated to reflux under nitrogen atmosphere at 110 °C for 72 h. Next, the reaction mixture was cooled down to room temperature, and then the mixture was extracted with H_2_O and ethyl acetate. The solvent was removed under reduced pressure and the product was recrystallized with ethanol.

### Synthesis of **4**

A mixture of **3**, imidazole (200 mg), *tert*-butylchlorodiphenylsilane (2 mL) and DMF (4.6 mL) in a two-necked flask was heated to reflux under nitrogen atmosphere at 40 °C for 24 h. Next, the reaction mixture was cooled down to room temperature, and then the mixture was extracted with H_2_O and ethyl acetate. The solvent was removed under reduced pressure and the product was recrystallized with ethanol.

^1^H NMR (400 MHz, CDCl_3_): *δ* = 7.99 (d, *J* = 8.0 Hz, 1H); 7.86–7.82 (m, 3H); 7.74–7.72 (m, 4H); 7.47–7.31 (m, 8H); 6.85 (d, *J* = 8.4 Hz, 2H); 1.12 (s, 9H). ESI-MS (*m*/*z*): calcd for C_29_H_27_NOSSi, 465.17; found, 464.97.

### Synthesis of **5**

Water (56.0 mL), anhydrous ethanol (112.0 mL) and acetic acid (112.0 mL) were added into a bottle. Then, iron powder (13.15 g, 0.235 mol) was added and the temperature was adjusted to 75 °C. Lastly, benzaldehyde (5.0 g, 0.033 mol) was poured into the bottle slowly and stirred for 15 min. Then the reaction mixture was cooled down to room temperature, the crude product was filtered and the filtrate was collected, which was then extracted with CH_2_Cl_2_ and water. Next, the organic phase was washed with saturated sodium bicarbonate solution (300 mL × 3 times) and deionized water (300 mL × 3 times), and then dried over anhydrous magnesium sulfate. The solvent was removed under reduced pressure to afford a yellow oily liquid. Yield: 3.25 g (81.0%).

^1^H NMR (400 MHz, CDCl_3_): *δ* = 9.81 (d, *J* = 0.8 Hz, 1H); 7.53 (dd, *J*_1_ = 1.6 Hz, *J*_2_ = 7.6 Hz, 1H); 7.29 (ddd, *J*_1_ = 1.6 Hz, *J*_2_ = 7.2 Hz, *J*_3_ = 8.4 Hz; 1H); 7.10 (brs, 2H); 6.70–6.85 (m, 1H); 6.63 (ddd, *J*_1_ = 1.2 Hz, *J*_2_ = 7.2 Hz, *J*_3_ = 8.0 Hz, 1H). ESI-MS (*m*/*z*): calcd for C_7_H_7_NO, 121.06; found, 120.98.

### Synthesis of **6**

Sodium hydroxide (1.0 g) was dissolved in anhydrous ethanol (15.0 mL). To this solution was added *o*-amino-benzaldehyde (600.0 mg, 4.96 mmol) and 2-acetylthiophene (688.0 mg, 5.46 mmol). The reaction mixture was refluxed at 80 °C with stirring for 12 h. The reaction mixture was cooled down to room temperature and the mixture was extracted with CH_2_Cl_2_, and then the solvent was removed under reduced pressure. Purifying the residue using silica gel column chromatography (CH_2_Cl_2_/PE, 1 : 10) gave a white solid. Yield: 835.4 mg (80.0%).

^1^H NMR (CDCl_3_): *δ* = 8.14 (d, *J* = 8.8 Hz, 1H); 8.09 (d, *J* = 8.8 Hz, 1H); 7.79 (d, *J* = 8.8 Hz, 1H); 7.76 (dd, *J*_1_ = 1.6 Hz, *J*_2_ = 8.4 Hz, 1H); 7.74 (dd, *J*_1_ = 0.8 Hz, *J*_2_ = 4.0 Hz, 1H); 7.69 (ddd, *J*_1_ = 1.6 Hz, *J*_2_ = 7.2 Hz, *J*_3_ = 8.4 Hz; 1H); 7.49 (ddd, *J*_1_ = 1.2 Hz, *J*_2_ = 6.8 Hz, *J*_3_ = 8.0 Hz; 1H); 7.47 (dd, *J*_1_ = 1.2 Hz, *J*_2_ = 5.2 Hz, 1H). ESI-MS (*m*/*z*): calcd for C_13_H_9_NS, 211.05; found, 211.16.

### Synthesis of **7**

Sodium hydroxide (1.0 g) was dissolved in anhydrous ethanol (15.0 mL). To this solution was added *o*-amino benzaldehyde (600.0 mg, 4.96 mmol) and acetophenone (0.6 mL, 4.28 mmol). The reaction mixture was refluxed at 80 °C with stirring for 12 h. The reaction mixture was cooled down to room temperature and extracted with CH_2_Cl_2_, and the solvent was removed under reduced pressure. The crude product was purified using column chromatography on silica gel (CH_2_Cl_2_/PE, 1 : 10) to yield the pure product as a white solid. Yield: 686.5 mg (81.0%).

^1^H NMR (CDCl_3_): *δ* = 8.23 (d, *J* = 8.4 Hz, 1H); 8.19–8.16 (m, 3H); 7.89 (d, *J* = 8.4 Hz, 1H); 7.84 (dd, *J*_1_ = 1.2 Hz, *J*_2_ = 8.0 Hz, 1H); 7.74 (ddd, *J*_1_ = 1.2 Hz, *J*_2_ = 6.8 Hz, *J*_3_ = 8.4 Hz, 1H); 7.56–7.52 (m, 3H); 7.47 (tt, *J*_1_ = 2.4 Hz, *J*_2_ = 7.2 Hz, 1H). ESI-MS (*m*/*z*): calcd for C_15_H_11_N, 205.09; found, 205.18.

### Synthesis of **[(2fppy)_2_Ir(μ-Cl)_2_Ir(2fppy)_2_]**

A mixture of IrCl_3_·3H_2_O (200.0 mg, 0.56 mmol) and 2-(2,4-difluorophenyl)pyridine (2fppy) (235.8 mg, 0.91 mmol) in 2-ethoxyethanol/water (8.0 mL, 3 : 1 v/v) was heated to reflux under nitrogen atmosphere for 24 h. Next, the reaction mixture was cooled down to room temperature, a large amount of water was added into the mixture, which was filtered. Then the residue was dried to give the desired yellow-green powder.

### Synthesis of **C1Cl**

A mixture of yellow powder of **[(2fppy)_2_Ir(μ-Cl)_2_Ir(2fppy)_2_]** (100.0 mg, 0.093 mmol) and auxiliary ligand *N*-hexyl-2-(2-pyridyl)benzimidazole (**2**) (52.0 mg, 0.186 mmol) in MeOH (3.0 mL) and CH_2_Cl_2_ (9.0 mL) was stirred for 4 h at 40 °C. The reaction mixture was cooled down to room temperature and evaporated to dryness. The crude material was purified by column chromatography over silica gel (CH_2_Cl_2_ → CH_2_Cl_2_/MeOH, 45 : 1), yielding the orange-red solid. Yield: 99.6 mg (60.4%).

^1^H NMR (400 MHz, DMSO-*d*_6_): *δ* = 8.68 (d, *J* = 8.4 Hz, 2H); 8.38 (dt, *J*_1_ = 1.2 Hz, *J*_2_ = 8.0 Hz, 1H); 8.31 (d, *J* = 8.4 Hz, 1H); 8.21 (d, *J* = 8.8 Hz, 1H); 8.05–7.94 (m, 4H); 7.83 (dd, *J*_1_ = 0.8 Hz, *J*_2_ = 5.6 Hz, 1H); 7.76–7.73 (m, 2H); 7.47 (ddd, *J*_1_ = 0.6 Hz, *J*_2_ = 6.4 Hz, *J*_3_ = 7.6 Hz, 1H); 7.22–7.16 (m, 3H); 7.04 (ddd, *J*_1_ = 2.4 Hz, *J*_2_ = 9.6 Hz, *J*_3_ = 12.4 Hz, 1H); 6.99 (ddd, *J*_1_ = 2.4 Hz, *J*_2_ = 9.6 Hz, *J*_3_ = 12.4 Hz, 1H); 6.25 (d, *J* = 8.0 Hz, 1H); 5.72 (dd, *J*_1_ = 2.4 Hz, *J*_2_ = 8.8 Hz, 1H); 5.62 (dd, *J*_1_ = 2.4 Hz, *J*_2_ = 8.8 Hz, 1H); 5.02–4.86 (m, 2H); 1.96–1.86 (m, 2H); 1.28–1.16 (m, 6H); 0.77 (t, *J* = 7.2 Hz, 3H). ^13^C{^1^H} NMR (100 MHz, DMSO): *δ* = 162.97 (dd, ^3^ *J*_C–F_ = 12.5 Hz, ^1^ *J*_C–F_ = 254.0 Hz); 162.39 (dd, ^3^ *J*_C–F_ = 12.5 Hz, ^1^ *J*_C–F_ = 253.6 Hz); 162.81 (d, ^3^ *J*_C–F_ = 6.6 Hz); 160.85 (dd, ^3^ *J*_C–F_ = 13.7 Hz, ^1^ *J*_C–F_ = 259.0 Hz); 160.51 (dd, ^3^ *J*_C–F_ = 12.4 Hz, ^1^ *J*_C–F_ = 257.0 Hz); 155.56 (d, ^3^ *J*_C–F_ = 6.2 Hz); 152.01; 151.93; 151.92; 151.68; 150.23; 149.81; 146.16; 140.63; 139.92; 139.73; 138.39; 136.37; 129.10; 128.27 (dd, *J*_1_ = 2.4 Hz, *J*_2_ = 4.1 Hz); 127.86; 126.18; 126.13; 125.92; 125.35; 124.42 (d, *J* = 11.1 Hz); 123.35 (d, *J* = 18.3 Hz); 122.96 (d, *J* = 19.6 Hz); 116.17; 113.73 (d, *J* = 16.7 Hz); 113.10; 113.16 (d, *J* = 15.8 Hz); 99.06 (t, ^2^ *J*_C–F_ = 27.1 Hz); 98.84 (t, ^2^ *J*_C–F_ = 27.1 Hz); 45.49; 30.78; 29.14; 25.49; 21.94; 13.79. ^19^F{^1^H} NMR (376.5 MHz, DMSO): *δ* = –106.49 (d, *J* = 10.17 Hz); –106.91 (d, *J* = 10.54 Hz); –108.61 (d, *J* = 10.54 Hz); –109.12 (d, *J* = 10.17 Hz). MALDI-TOF-MS (*m*/*z*): calcd for C_40_H_33_IrF_4_N_5_Cl, 887.20; found, 852.06 (M – PF_6_)^+^. Anal. calcd, C 54.14, H 3.75, N 7.89; found, C 54.19, H 3.81, N 7.92.

### Synthesis of **[(ppy)_2_Ir(μ-Cl)_2_Ir(ppy)_2_]**

A mixture of IrCl_3_·3H_2_O (200.0 mg, 0.56 mmol) and 2-phenylpyridine (ppy) (173.8 mg, 0.16 mmol) in 2-ethoxyethanol/water (8.0 mL, 3 : 1 v/v) was heated to reflux under nitrogen atmosphere for 24 h. Next, the reaction mixture was cooled down to room temperature, a large amount of water was added into the mixture, which was filtered. Then the residue was dried to give the desired yellow powder. Yield: 353.8 mg (59.0%).

### Synthesis of **C2Cl**

A mixture of yellow powder of **[(ppy)_2_Ir(μ-Cl)_2_Ir(ppy)_2_]** (100.0 mg, 0.093 mmol) and auxiliary ligand 2,2-dipyridyl (dpy) (29.0 mg, 0.186 mmol) in MeOH (3.0 mL) and CH_2_Cl_2_ (9.0 mL) was stirred for 4 h at 40 °C. The reaction mixture was then cooled to room temperature and evaporated to dryness. The crude material was purified by column chromatography over silica gel (CH_2_Cl_2_ → CH_2_Cl_2_/MeOH, 45 : 1), yielding the orange-red solid. Yield: 41.9 mg (65.4%).

^1^H NMR (400 MHz, DMSO-*d*_6_): *δ* = 8.90 (d, *J* = 8.4 Hz, 2H); 8.29–8.25 (m, 4H); 7.96–7.91 (m, 4H); 7.87 (dd, *J*_1_ = 0.8 Hz, *J*_2_ = 5.2 Hz, 2H); 7.69 (ddd, *J*_1_ = 0.8 Hz, *J*_2_ = 5.2 Hz, *J*_3_ = 6.4 Hz, 2H); 7.61 (d, *J* = 5.2 Hz, 2H); 7.16 (ddd, *J*_1_ = 1.2 Hz, *J*_2_ = 5.6 Hz, *J*_3_ = 7.2 Hz, 2H); 7.02 (dt, *J*_1_ = 1.2 Hz, *J*_2_ = 8.0 Hz, 2H); 6.90 (dt, *J*_1_ = 1.2 Hz, *J*_2_ = 7.6 Hz, 2H); 6.19 (dd, *J*_1_ = 0.8 Hz, *J*_2_ = 7.6 Hz, 2H). ^13^C{^1^H} NMR (100 MHz, DMSO-*d*_6_): *δ* = 166.86; 155.392; 150.49; 149.87; 148.91; 143.84; 139.75; 138.85; 131.12; 130.31; 128.73; 125.14; 125.07; 123.99; 122.36; 120.11. MALDI-TOF-MS (*m*/*z*): calcd for C_32_H_24_IrN_4_Cl, 692.13; found, 656.99 (M – PF_6_)^+^. Anal. calcd, C 55.52, H 3.49, N 8.09; found, C 55.07, H 3.90, N 7.98.

### Synthesis of ***n*-Bu_4_NA2**

The complex [Ir(ppy)_2_(CN)_2_]TBA, where TBA is the tetrabutylammonium ion, was prepared according to the literature procedure, with some modifications.^39^ The dimeric iridium(iii) complex **[(ppy)_2_Ir(μ-Cl)_2_Ir(ppy)_2_]** (200.0 mg) was dissolved in 30.0 mL of dichloromethane solvent under nitrogen. To this solution was added tetrabutylammonium cyanide (100.0 mg) ligand. The reaction mixture was refluxed with stirring for 8 h. The reaction mixture was cooled down to room temperature, and then the solution was washed three times with distilled H_2_O, and extracted with dichloromethane. The organic layer was dried over Na_2_SO_4_, and the solvents were removed *in vacuo*. The crude material was purified by column chromatography over silica gel (CH_2_Cl_2_ → CH_2_Cl_2_/MeOH, 50 : 1), yielding the green solid. Yield: 114.9 mg (77.5%).

^1^H NMR (400 MHz, DMSO-*d*_6_): *δ* = 9.52 (dd, *J*_1_ = 0.8 Hz, *J*_2_ = 6.0 Hz, 2H); 8.07 (d, *J* = 8.4 Hz, 2H); 7.89 (dt, *J*_1_ = 1.6 Hz, *J*_2_ = 7.6 Hz, 2H); 7.66 (d, *J* = 7.6 Hz, 2H); 7.31 (dt, *J*_1_ = 5.6 Hz, *J*_2_ = 7.2 Hz, 2H); 6.73 (dt, *J*_1_ = 1.2 Hz, *J*_2_ = 6.8 Hz, 2H); 6.61 (dt, *J*_1_ = 1.2 Hz, *J*_2_ = 7.2 Hz, 2H); 6.08 (dd, *J*_1_ = 1.2 Hz, *J*_2_ = 7.6 Hz, 2H); 3.17–3.13 (m, 8H); 1.60–1.52 (m, 8H); 1.34–1.25 (m, 8H); 0.93 (t, *J* = 7.6 Hz, 12H). ^13^C{^1^H} NMR (100 MHz, DMSO-*d*_6_): *δ* = 168.02; 163.78; 153.48; 144.55; 136.42; 131.48; 130.88; 128.50; 123.81; 122.74; 120.24; 119.14; 57.71; 23.21; 19.36; 13.64. MALDI-TOF-MS, ESI-MS (*m*/*z*): calcd for C_40_H_52_IrN_5_, 795.38; found, 553.17 (M – *n*-Bu_4_N)^–^, 242.42 (*n*-Bu_4_N)^+^. Anal. calcd, C 60.57, H 6.49, N 8.56; found, C 60.42, H 6.59, N 8.81.

### Synthesis of **[(thq)_2_Ir(μ-Cl)_2_Ir(thq)_2_]**

A mixture of IrCl_3_·3H_2_O (385.0 mg, 1.10 mmol) and 2-(thiophenyl)quinoline (500.2 mg, 2.37 mmol) in 2-ethoxyethanol/water (8.0 mL, 3 : 1 v/v) was heated to reflux under nitrogen atmosphere for 24 h. Next, the reaction mixture was cooled down to room temperature and a large amount of water was added into the mixture. The mixture was filtered. Then the residue was dried to give the desired red powder. Yield: 650.8 mg (85.0%).

### Synthesis of ***n*-Bu_4_NA1**

The dimeric iridium(iii) complex **[(thq)_2_Ir(μ-Cl)_2_Ir(thq)_2_]** (200.0 mg, 0.15 mmol) and tetrabutylammonium cyanide (495.7 mg, 1.85 mmol) ligand were dissolved in 30.0 mL of dichloromethane solvent under nitrogen. The reaction mixture was refluxed with stirring for 8 h. Then the mixture was cooled to room temperature and extracted with CH_2_Cl_2_ and water. The crude product was purified through column chromatography on silica gel (CH_2_Cl_2_ → CH_2_Cl_2_/MeOH, 50 : 1) to give the pure product as a red solid. Yield: 85.6 mg (61.0%).

^1^H NMR (400 MHz, CDCl_3_): *δ* = 9.99 (d, *J* = 9.2 Hz, 2H); 7.97 (d, *J* = 8.4 Hz, 2H); 7.68–7.65 (m, 4H); 7.60 (d, *J* = 8.4 Hz, 2H); 7.42 (t, *J* = 7.2 Hz, 2H); 7.03 (d, *J* = 4.4 Hz, 2H); 6.09 (d, *J* = 4.4 Hz, 2H); 3.11–3.07 (m, 8H); 1.47–1.39 (m, 8H); 1.11–1.06 (m, 8H); 0.77 (t, *J* = 7.6 Hz, 12H). ^13^C{^1^H} NMR (100 MHz, CDCl_3_): *δ* = 170.56; 167.67; 150.11; 141.51; 137.76; 133.34; 131.12; 130.42; 128.95; 127.68; 125.76; 124.71; 117.47; 58.48; 23.80; 19.47; 13.59. MALDI-TOF-MS, ESI-MS (*m*/*z*): calcd for C_44_H_52_IrN_5_S_2_, 907.33; found, 665.17 (M – *n*-Bu_4_N)^–^, 242.42 (*n*-Bu_4_N)^+^. Anal. calcd, C 58.25, H 5.78, N 7.72; found, C 58.36, H 5.89, N 7.61.

### Synthesis of **[(phq)_2_Ir(μ-Cl)_2_Ir(phq)_2_]**

A mixture of IrCl_3_·3H_2_O (385.0 mg, 1.10 mmol) and 2-phenylquinoline (phq) (500.5 mg, 2.37 mmol) in 2-ethoxyethanol/water (8.0 mL, 3 : 1 v/v) was heated to reflux under nitrogen atmosphere for 24 h. Next, the reaction mixture was cooled down to room temperature and a large amount of water was added into the mixture. The mixture was filtered. Then the residue was dried to give the desired red powder. Yield: 600.5 mg (77.1%).

### Synthesis of **C3Cl**

A mixture of yellow powder of **[(phq)_2_Ir(μ-Cl)_2_Ir(phq)_2_]** (100.0 mg, 0.093 mmol) and auxiliary ligand 2,2′-biquinoline (bql) (52.0 mg, 0.20 mmol) in MeOH (3 mL) and CH_2_Cl_2_ (9 mL) was stirred for 4 h at 40 °C. The reaction mixture was cooled down to room temperature and then evaporated to dryness. The crude material was purified by column chromatography over silica gel (CH_2_Cl_2_ → CH_2_Cl_2_/MeOH, 45 : 1), yielding the orange-red solid. Yield: 41.0 mg (44.8%).

^1^H NMR (400 MHz, DMSO-*d*_6_): *δ* = 8.71 (d, *J* = 8.8 Hz, 2H); 8.49 (d, *J* = 8.8 Hz, 2H); 8.31 (d, *J* = 8.8 Hz, 2H); 8.20 (d, *J* = 8.8 Hz, 2H); 8.06 (d, *J* = 8.8 Hz, 2H); 7.96 (d, *J* = 8.0 Hz, 2H); 7.91 (d, *J* = 8.0 Hz, 2H); 7.56 (t, *J* = 7.6 Hz, 2H); 7.51 (d, *J* = 8.8 Hz, 2H); 7.43–7.37 (m, 4H); 7.09 (t, *J* = 8.0 Hz, 2H); 7.01 (t, *J* = 7.2 Hz, 2H); 6.86–6.81 (m, 4H); 6.31 (d, *J* = 7.2 Hz, 2H). ^13^C{^1^H} NMR (100 MHz, DMSO): *δ* = 169.88; 159.57; 149.09; 146.72; 145.44; 140.99; 140.59; 137.55; 131.01; 130.96; 130.43; 129.55; 129.43; 129.02; 128.99; 128.34; 128.24; 127.60; 127.05; 126.46; 124.47; 123.00; 121.37; 117.65. MALDI-TOF-MS (*m*/*z*): calcd for C_48_H_32_IrN_4_Cl, 892.20; found, 857.03 (M – PF_6_)^+^. Anal. calcd, C 64.60, H 3.61, N 6.28; found, C 64.50, H 3.52, N 6.54.

### Synthesis of **[(Si)_2_Ir(μ-Cl)_2_Ir(Si)_2_]**

A mixture of IrCl_3_·3H_2_O (200.0 mg, 0.56 mmol) and **7** (173.8 mg, 0.16 mL, 1.12 mmol) in 2-ethoxyethanol/water (8.0 mL, 3 : 1 v/v) was heated to reflux under nitrogen atmosphere for 24 h. Next, the reaction mixture was cooled down to room temperature, and a large amount of water was added into the mixture, which was then filtered. Then the residue was dried to give the desired yellow powder. Yield: 147.1 mg (68.0%).

### Synthesis of ***n*–Bu_4_NA3**

The dimeric iridium(iii) complex **[(Si)_2_Ir(μ-Cl)_2_Ir(Si)_2_]** (200.0 mg) was dissolved in 30.0 mL of dichloromethane solvent under nitrogen. To this solution was added tetrabutylammonium cyanide (100.0 mg) ligand. The reaction mixture was refluxed with stirring for 8 h. The mixture was cooled down to room temperature, and then the solution was washed three times with distilled H_2_O, and extracted with dichloromethane. The organic layer was dried over Na_2_SO_4_, and the solvents were removed *in vacuo*. The crude material was purified by column chromatography over silica gel (CH_2_Cl_2_ → CH_2_Cl_2_/MeOH, 50 : 1) to give a green solid. Yield: 115.4 mg (77.5%).

^1^H NMR (400 MHz, DMSO-*d*_6_): *δ* = 9.26 (dd, *J*_1_ = 2.4 Hz, *J*_2_ = 7.2 Hz, 2H); 8.00 (dd, *J*_1_ = 2.4 Hz, *J*_2_ = 7.2 Hz, 2H); 7.50–7.30 (m, 18H); 7.23–7.19 (m, 8H); 6.32 (dd, *J*_1_ = 2.4 Hz, *J*_2_ = 8.4 Hz, 2H); 5.79 (d, *J* = 2.4 Hz, 2H); 3.18–3.14 (m, 8H); 1.61–1.52 (m, 8H); 1.35–1.26 (m, 8H); 0.93 (t, *J* = 7.2 Hz, 12H); 0.75 (s, 18H). ^13^C{^1^H} NMR (100 MHz, CDCl_3_): *δ* = 179.17; 166.57; 156.95; 151.727; 135.12; 135.08; 134.67; 133.29; 133.13; 130.65; 129.41; 129.38; 127.44; 126.67; 126.58; 124.11; 121.43; 113.44; 58.59; 26.53; 23.77; 19.51; 13.59. MALDI-TOF-MS, ESI-MS (*m*/*z*): calcd for C_76_H_88_IrN_5_S_2_O_2_Si_2_, 1415.55; found, 1173.67 (M – *n*-Bu_4_N)^–^, 242.42 (*n*-Bu_4_N)^+^. Anal. calcd, C 64.46, H 6.26, N 4.95; found, C 64.21, H 6.39, N 5.11.

### Synthesis of **IP1**

**C1Cl** (38.1 mg, 0.043 mmol) and ***n*-Bu_4_NA1** (39.0 mg, 0.043 mmol) were added to water (15.0 mL) and CH_2_Cl_2_ (15.0 mL). The reaction mixture was stirred for 1 h at room temperature and then extracted with deionized water. The combined organic extracts were concentrated by rotary evaporation. The resulting solid was washed with *n*-hexane to afford **IP1** as a red solid. Yield: 39.6 mg (60.7%).

^1^H NMR (400 MHz, DMSO-*d*_6_): *δ* = 9.90 (d, *J* = 8.8 Hz, 2H); 8.64 (d, *J* = 8.4 Hz, 2H); 8.34–8.29 (m, 3H); 8.21 (d, *J* = 8.8 Hz, 1H); 8.03–7.91 (m, 6H); 7.81 (d, *J* = 5.2 Hz, 1H); 7.72–7.67 (m, 6H); 7.56 (t, *J* = 6.8 Hz, 1H); 7.46 (t, *J* = 8.0 Hz, 1H); 7.39–7.35 (m, 1H); 7.23–7.15 (m, 5H); 7.02 (ddd, *J*_1_ = 2.0 Hz, *J*_2_ = 9.2 Hz, *J*_3_ = 12.0 Hz, 1H); 6.96 (ddd, *J*_1_ = 2.4 Hz, *J*_2_ = 9.6 Hz, *J*_3_ = 12.4 Hz, 1H); 6.24 (d, *J* = 8.4 Hz, 1H); 5.71 (d, *J* = 4.8 Hz, 2H); 5.61 (dd, *J*_1_ = 2.4 Hz, *J*_2_ = 8.4 Hz, 1H); 4.97–4.84 (m, 2H); 1.94–1.86 (m, 2H); 1.25–1.21 (m, 6H); 0.75 (t, *J* = 6.8 Hz, 3H). ^19^F{^1^H} NMR (376.5 MHz, DMSO): *δ* = –106.49 (d, *J* = 10.17 Hz); –106.91 (d, *J* = 10.54 Hz); –108.61 (d, *J* = 10.54 Hz); –109 (d, *J* = 10.17 Hz). Because of the asymmetry of the ancillary ligand in **C1**, the two C^N ligands are non-chemically equivalent. In addition, a coupling process exists between the fluorine nucleus and carbon nucleus, and we did not obtain the ^13^C{^1^H} NMR of **IP1**. MALDI-TOF-MS, ESI-MS (*m*/*z*): calcd for C_68_H_49_Ir_2_F_4_N_9_S_2_, 1517.27; found, 665.08 (A1^–^), 852.11 (C1^+^). Anal. calcd, C 53.85, H 3.26, N 8.31; found, C 53.63, H 3.09, N 8.07.

### Synthesis of **IP2**

**C2Cl** (29.7 mg, 0.043 mmol) and ***n*-Bu_4_NA2** (34.1 mg, 0.043 mmol) were added to water (15.0 mL) and CH_2_Cl_2_ (15.0 mL). The reaction mixture was stirred for 1 h at room temperature and then extracted with deionized water. The combined organic extracts were concentrated by rotary evaporation. The resulting solid was washed with *n*-hexane to afford **IP2** as a yellow solid. Yield: 32.3 mg (62.3%).

^1^H NMR (400 MHz, DMSO-*d*_6_): *δ* = 9.53 (d, *J* = 5.6 Hz, 2H); 8.86 (d, *J* = 8.4 Hz, 2H); 8.27–8.24 (m, 4H); 8.06 (d, *J* = 8.0 Hz, 2H); 7.95–7.86 (m, 8H); 7.70–7.61 (m, 6H); 7.30 (t, *J* = 6.0 Hz, 2H); 7.15 (t, *J* = 6.8 Hz, 2H); 7.02 (t, *J* = 7.6 Hz, 2H); 6.90 (t, *J* = 7.2 Hz, 2H); 6.72 (t, *J* = 7.2 Hz, 2H); 6.61 (t, *J* = 7.2 Hz, 2H); 6.19 (d, *J* = 7.6 Hz, 2H); 6.08 (d, *J* = 7.2 Hz, 2H). ^13^C{^1^H} NMR (100 MHz, DMSO): *δ* = 167.93; 166.83; 163.83; 155.36; 153.32; 150.48; 149.83; 148.88; 144.42; 143.82; 139.69; 138.82; 136.13; 131.08; 130.76; 130.28; 128.69; 128.29; 125.12; 125.06; 123.97; 123.63; 122.53; 122.32; 120.08; 119.95; 118.93. MALDI-TOF-MS, ESI-MS (*m*/*z*): calcd for C_56_H_40_Ir_2_F_4_N_8_, 1210.26; found, 657.25 (A2^–^), 553.25 (C2^+^). Anal. calcd, C 55.61, H 3.33, N 9.27; found, C 55.20, H 3.39, N 8.91.

### Synthesis of **IP3**

**C3Cl** (40.1 mg, 0.045 mmol) and ***n*-Bu_4_NA2** (35.7 mg, 0.045 mmol) were added to water (15.0 mL) and CH_2_Cl_2_ (15.0 mL). The reaction mixture was stirred for 1 h at room temperature and then extracted with deionized water. The combined organic extracts were concentrated by rotary evaporation. The resulting solid was washed with *n*-hexane to afford **IP3** as a red solid. Yield: 35.6 mg (56.2%).

^1^H NMR (400 MHz, DMSO-*d*_6_): *δ* = 9.51 (d, *J* = 5.2 Hz, 2H); 8.70 (d, *J* = 8.8 Hz, 2H); 8.47 (d, *J* = 8.8 Hz, 2H); 8.30 (d, *J* = 8.8 Hz, 2H); 8.19 (d, *J* = 8.8 Hz, 2H); 8.08–8.04 (m, 4H); 7.95 (dd, *J*_1_ = 0.8 Hz, *J*_2_ = 8.0 Hz, 2H); 7.91 (d, *J* = 7.2 Hz, 2H); 7.85 (ddd, *J*_1_ = 1.6 Hz, *J*_2_ = 6.8 Hz, *J*_3_ = 8.4 Hz, 2H); 7.66 (d, *J* = 7.6 Hz, 2H); 7.56 (ddd, *J*_1_ = 0.8 Hz, *J*_2_ = 6.8 Hz, *J*_3_ = 8.0 Hz; 2H); 7.51 (d, *J*_1_ = 8.8 Hz, 2H); 7.43–7.36 (m, 4H); 7.30 (t, *J* = 7.2 Hz, 2H); 7.09 (ddd, *J*_1_ = 1.6 Hz, *J*_2_ = 6.8 Hz, *J*_3_ = 8.8 Hz; 2H); 7.01 (dt, *J*_1_ = 1.2 Hz, *J*_2_ = 7.2 Hz, 2H); 6.86–6.81 (m, 4H); 6.73 (dt, *J*_1_ = 1.2 Hz, *J*_2_ = 7.6 Hz, 2H); 6.61 (dt, *J*_1_ = 0.8 Hz, *J*_2_ *J*_2_ = 7.2 Hz, 2H); 6.30 (dd, *J*_1_ = 0.8 Hz, *J*_2_ = 8.0 Hz, 2H); 6.07 (dd, *J*_1_ = 0.8 Hz, *J*_2_ = 7.6 Hz, 2H). ^13^C{^1^H} NMR (100 MHz, DMSO): *δ* = 169.88; 168.05; 159.57; 153.46; 149.09; 146.72; 145.44; 144.59; 140.99; 140.59; 137.55; 136.51; 131.82; 131.01; 130.96; 130.91; 130.43; 129.55; 129.43; 129.02; 128.99; 128.58; 128.34; 128.24; 127.60; 127.05; 126.46; 124.47; 123.89; 123.00; 122.83; 121.37; 120.34; 119.22; 119.08; 117.65. MALDI-TOF-MS, ESI-MS (*m*/*z*): calcd for C_72_H_48_Ir_2_N_8_, 1410.33; found, 553.17 (A2^–^), 857.11 (C3^+^). Anal. calcd, C 61.35, H 3.43, N 7.95; found, C 60.97, H 3.79, N 7.86.

### Synthesis of **IP4**

**C1Cl** (39.9 mg, 0.045 mmol) and ***n*-Bu_4_NA2** (35.7 mg, 0.045 mmol) were added to water (15.0 mL) and CH_2_Cl_2_ (15.0 mL). The reaction mixture was stirred for 1 h at room temperature and then extracted with deionized water. The combined organic extracts were concentrated by rotary evaporation. The resulting solid was washed with *n*-hexane to afford **IP4** as a yellow solid. Yield: 29.2 mg (46.2%).

^1^H NMR (400 MHz, DMSO-*d*_6_): *δ* = 9.52 (d, *J* = 6.0 Hz, 2H); 8.66 (d, *J* = 8.4 Hz, 2H); 8.37–8.34 (m, 1H); 8.30 (d, *J* = 7.6 Hz, 1H); 8.21 (d, *J* = 8.4 Hz, 1H); 8.07–7.86 (m, 8H); 7.83 (d, *J* = 5.6 Hz, 1H); 7.73–7.71 (m, 2H); 7.65 (d, *J* = 7.6 Hz, 2H); 7.47 (t, *J* = 7.2 Hz, 1H); 7.31–7.15 (m, 5H); 7.06–6.96 (m, 2H); 6.71 (dt, *J*_1_ = 1.2 Hz, *J*_2_ = 6.8 Hz, 2H); 6.60 (dt, *J*_1_ = 0.8 Hz, *J*_2_ = 7.2 Hz, 2H); 6.25 (d, *J* = 8.0 Hz, 1H); 6.08 (dd, *J*_1_ = 1.2 Hz, *J*_2_ = 7.6 Hz, 2H); 5.75 (m, 1H); 5.62 (dd, *J*_1_ = 1.6 Hz, *J*_2_ = 8.4 Hz, 1H); 5.02–4.84 (m, 2H); 1.98–1.84 (m, 2H); 1.28–1.19 (m, 6H); 0.77 (t, *J* = 7.2 Hz, 3H). ^19^F{^1^H} NMR (376.5 MHz, DMSO): *δ* = –106.49 (d, *J* = 10.17 Hz); –106.91 (d, *J* = 10.54 Hz); –108.61 (d, *J* = 10.54 Hz); –109.12 (d, *J* = 10.17 Hz). Because of the asymmetry of the ancillary ligand in **C1**, the two C^N ligands are non-chemically equivalent. In addition, a coupling process exists between the fluorine nucleus and carbon nucleus, and there is a low signal to noise ratio in the spectrum of ^13^C{^1^H} NMR. MALDI-TOF-MS, ESI-MS (*m*/*z*): calcd for C_64_H_49_Ir_2_F_4_N_9_, 1405.32; found, 553.08 (A2^–^), 852.11 (C1^+^). Anal. calcd, C 54.73, H 3.52, N 8.98; found, C 54.27, H 3.64, N 8.56.

### Synthesis of **IP5**

**C2Cl** (29.7 mg, 0.043 mmol) and ***n*-Bu_4_NA3** (60.8 mg, 0.043 mmol) were added to water (15.0 mL) and CH_2_Cl_2_ (15.0 mL). The reaction mixture was stirred for 1 h at room temperature and then extracted with deionized water. The combined organic extracts were concentrated by rotary evaporation. The resulting solid was washed with *n*-hexane to afford **IP5** as a yellow solid. Yield: 46.5 mg (59.2%).

^1^H NMR (400 MHz, DMSO-*d*_6_): *δ* = 9.25 (dd, *J*_1_ = 1.2 Hz, *J*_2_ = 8.0 Hz; 2H); 8.87 (d, *J* = 8.4 Hz; 2H); 8.28–8.24 (m; 4H); 8.01–7.99 (m; 2H); 7.95–7.91 (m; 4H); 7.86 (dd, *J*_1_ = 1.2 Hz, *J*_2_ = 5.6 Hz; 2H); 7.68 (ddd, *J*_1_ = 0.8 Hz, *J*_2_ = 5.6 Hz, *J*_3_ = 6.8 Hz; 2H); 7.61 (dd, *J*_1_ = 0.8 Hz, *J*_2_ = 5.6 Hz; 2H); 7.50–7.42 (m; 6H); 7.39–7.30 (m; 12H); 7.25–7.19 (m; 8H); 7.15 (ddd, *J*_1_ = 1.6 Hz, *J*_2_ = 6.0 Hz, *J*_3_ = 7.6 Hz; 2H); 7.02 (dt, *J*_1_ = 1.2 Hz, *J*_2_ = 7.6 Hz; 2H); 6.90 (dt, *J*_1_ = 1.2 Hz, *J*_2_ = 7.6 Hz; 2H); 6.32 (dd, *J*_1_ = 2.4 Hz, *J*_2_ = 8.0 Hz; 2H); 6.19 (dd, *J*_1_ = 1.2 Hz, *J*_2_ = 7.6 Hz; 2H); 5.78 (d, *J* = 2.4 Hz; 2H); 0.75 (s; 18H). ^13^C{^1^H} NMR (100 MHz, DMSO): *δ* = 178.51; 166.94; 156.32; 155.46; 151.23; 150.57; 149.93; 148.96; 143.92; 139.82; 138.95; 134.72; 134.68; 132.54; 132.51; 131.20; 130.41; 130.32; 129.96; 129.36; 128.78; 127.81; 126.80; 126.72; 125.21; 125.11; 124.93; 124.06; 123.15; 122.85; 122.68; 122.48; 120.19; 113.15; 26.67; 19.14. MALDI-TOF-MS, ESI-MS (*m*/*z*): calcd for C_92_H_76_Ir_2_O_2_N_8_S_2_Si_2_, 1830.43; found, 1173.67 (A3^–^), 657.01 (C2^+^). Anal. calcd, C 60.37, H 4.19, N 6.12; found, C 60.11, H 4.36, N 6.30.

**IP6** and ***n*-Bu_4_NA4** were synthesized according to the literature.^39^

### Synthesis of ***n*-Bu_4_NA4**

^1^H NMR (400 MHz, DMSO-*d*_6_): *δ* = 9.54 (dd, *J*_1_ = 1.2 Hz, *J*_2_ = 6.0 Hz; 2H); 8.22 (d, *J* = 8.4 Hz; 2H); 8.04 (dt, *J*_1_ = 1.2 Hz, *J*_2_ = 7.6 Hz; 2H); 7.45 (ddd, *J*_1_ = 1.2 Hz, *J*_2_ = 6.0 Hz, *J*_3_ = 7.0 Hz; 2H); 6.61 (ddd, *J*_1_ = 2.4 Hz, *J*_2_ = 9.2 Hz, *J*_3_ = 12.0 Hz; 2H); 5.54 (dd, *J*_1_ = 2.4 Hz, *J*_2_ = 8.0 Hz; 2H); 3.17–3.13 (m; 8H); 1.59–1.51 (m; 8H); 1.32–1.24 (m; 8H); 0.92 (t; 12H). ^13^C{^1^H} NMR (100 MHz, DMSO): *δ* = 169.79–169.66 (m); 164.36 (d, ^3^ *J*_C–F_ = 7.3 Hz); 162.82 (dd, ^3^ *J*_C–F_ = 11.1 Hz, ^1^ *J*_C–F_ = 252.6 Hz); 161.12 (dd, ^3^ *J*_C–F_ = 11.9 Hz, ^1^ *J*_C–F_ = 258.2 Hz); 154.19; 138.044; 128.51; 128.29 (t, ^4^ *J*_C–F_ = 2.2 Hz); 123.86; 122.92 (d, ^2^ *J*_C–F_ = 20.3 Hz); 112.36 (dd, ^4^ *J*_C–F_ = 2.5 Hz, ^2^ *J*_C–F_ = 15.2 Hz); 97.02 (t, ^2^ *J*_C–F_ = 26.2 Hz); 57.98; 23.51; 19.65; 13.95. ^19^F{^1^H} NMR (376.5 MHz, DMSO): *δ* = –109.91 (d, *J* = 9.05 Hz); –110.96 (d, *J* = 9.42 Hz). MALDI-TOF-MS, ESI-MS (*m*/*z*): calcd for C_40_H_48_IrN_5_F_4_, 795.38; found, 624.41 (M – *n*-Bu_4_N)^–^, 242.42 (*n*-Bu_4_N)^+^. Anal. calcd, C 55.41, H 5.58, N 8.08; found, C 55.72, H 5.70, N 8.21.

### Synthesis of **IP6**

^1^H NMR (400 MHz, CDCl_3_): *δ* = 9.74 (d, *J* = 5.6 Hz; 2H); 8.95 (d, *J* = 8.4 Hz; 2H); 8.06–8.17 (m; 4H); 7.86–7.91 (m; 4H); 7.73 (t, *J* = 8.0 Hz; 2H); 7.67 (d, *J* = 8.4 Hz; 2H); 7.62 (t, *J* = 8.0 Hz; 2H); 7.47 (d, *J* = 5.6 Hz; 2H); 7.34 (t, *J* = 6.4 Hz; 2H); 7.03 (t, *J* = 7.6 Hz; 2H); 6.99–6.90 (m; 6H); 6.28 (d, *J* = 7.6 Hz; 2H); 6.18 (ddd, *J*_1_ = 2.0 Hz, *J*_2_ = 9.2 Hz, *J*_3_ = 12.4 Hz; 2H); 5.72 (dd, *J*_1_ = 2.4 Hz, *J*_2_ = 8.4 Hz; 2H). ^19^F{^1^H} NMR (376.5 MHz, CDCl_3_): *δ* = –106.57 (d, *J* = 10.56 Hz); –108.8 (d, *J* = 10.56 Hz). MALDI-TOF-MS (*m*/*z*): calcd for C_56_H_36_Ir_2_F_4_N_8_, 1282.23; found, 624.44 (A4^–^), 656.99 (C2^+^). Anal. calcd, C 52.49, H 2.83, N 8.74; found, C 52.09, H 2.66, N 6.49.
