# Supplementary material for: Luminescent ion pairs with tunable emission colors for light-emitting devices and electrochromic switches
Source: Chem Sci. 2016 Aug 15;8(1):348–60. doi: 10.1039/c6sc02837c (PMC5365054; doi:10.1039/c6sc02837c)
Supplement: Supplementary file 1 [file SC-008-C6SC02837C-s001.pdf]

Electronic Supplementary Information

Luminescent ion pairs with tunable emission colors for light-emitting devices and electrochromic switches

Song Guo,[a] Tianci Huang,[a] Shujuan Liu,[a] Kenneth Yin Zhang,[a] Huiran Yang,[a] Jianmei Han,[a] Qiang Zhao,*[a] and Wei Huang*[ab]

Table S1. The emission wavelengths, lifetimes and quantum yields of the positive and negative complexes and IPs (10^{-5} M in acetonitrile).

Sample	λ (nm)	τ (ns)	Φ (%)
**C1Cl**	550	323	21
**C2Cl**	580	62	27
**C3Cl**	630	352	8
***n*-Bu$_4$NA1**	600	163	17
***n*-Bu$_4$NA2**	472 / 510	32	11
***n*-Bu$_4$NA3**	520 / 570	200	20
***n*-Bu$_4$NA4**	452 / 479	469	31
**IP1**	550	305	8
	600	248	
**IP2**	472 / 510	43	35
	580	59	
**IP3**	472 / 510	34	13
	630	337	
**IP4**	472 / 510	40	37
	550	301	
**IP5**	520	190	20
	580	162	
**IP6**	580	102	22
	452 / 479	416	

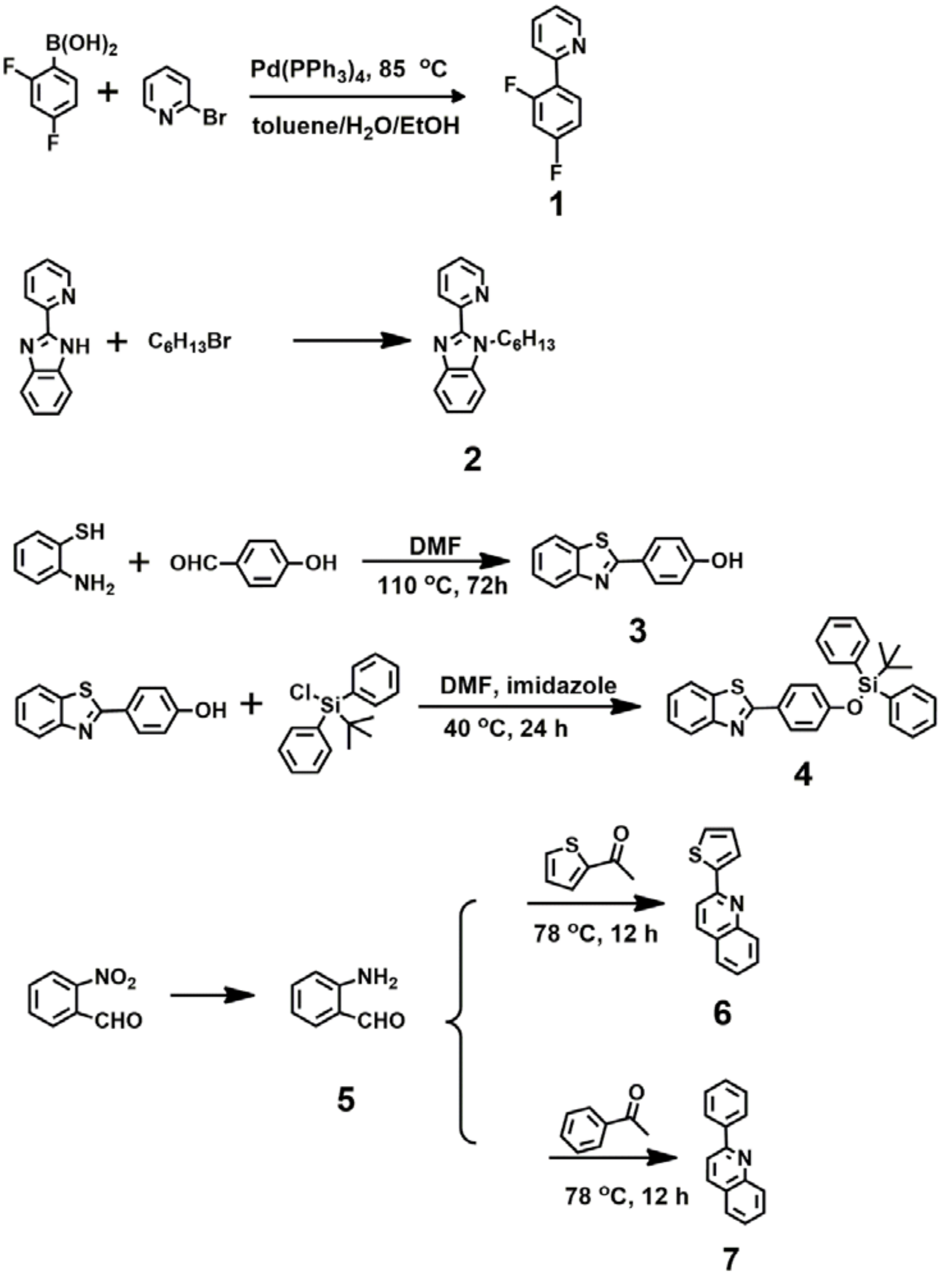

Scheme S1 The synthetic routes of ligands.

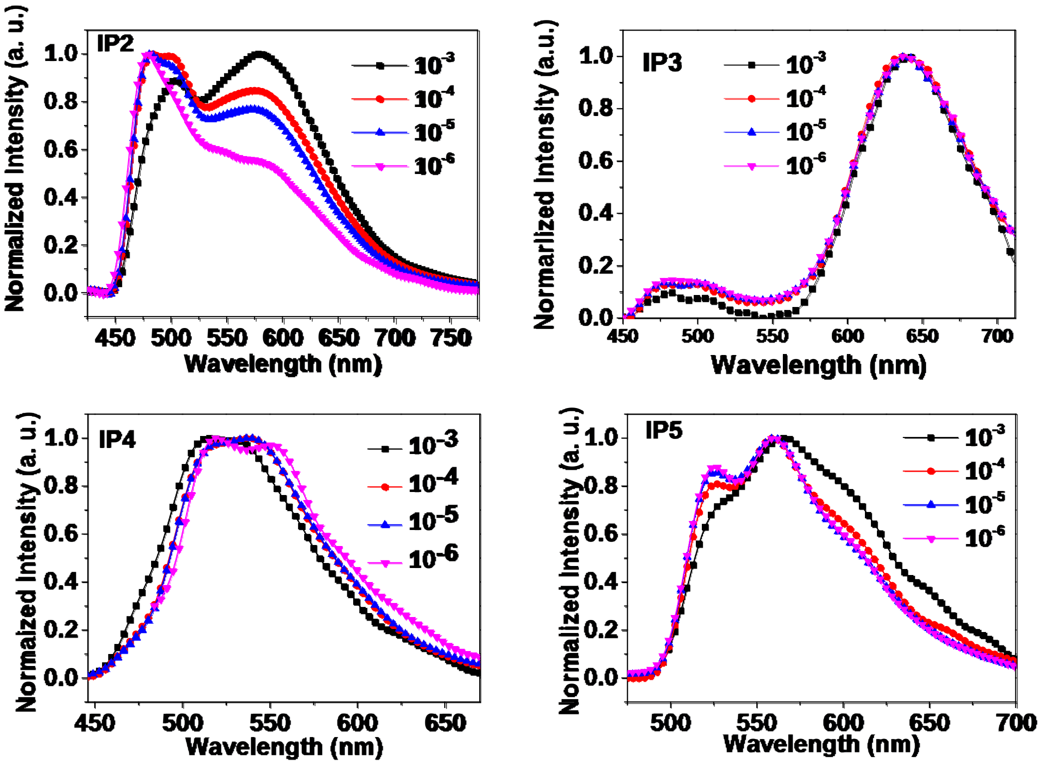

Fig. S1. The PL spectra of the IPs at different concentrations in acetonitrile**.**

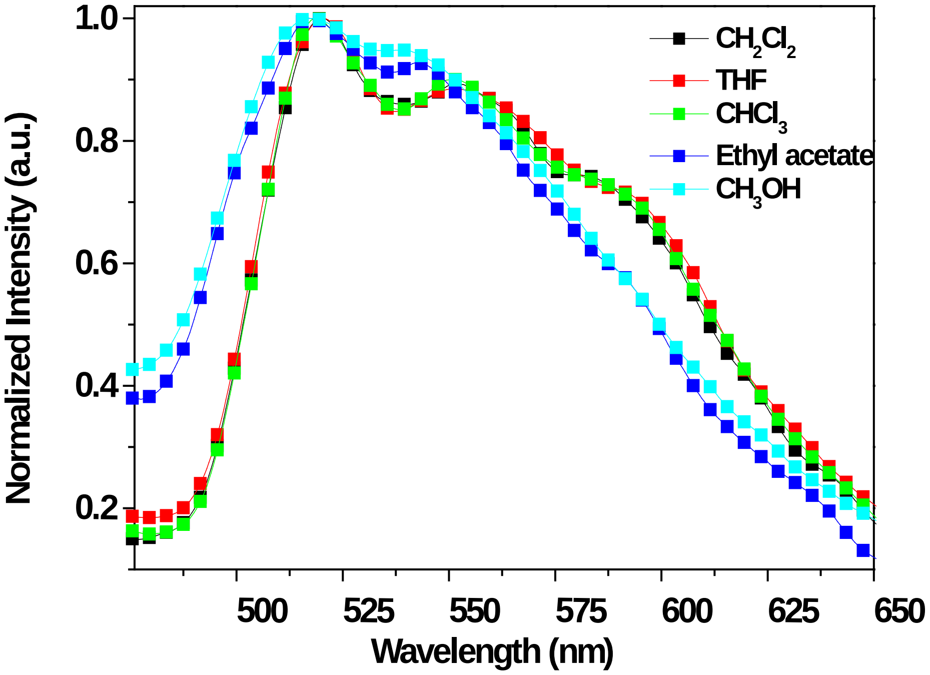

Fig. S2. The emission spectra of **IP1** in different solvents (10^{-5} M).

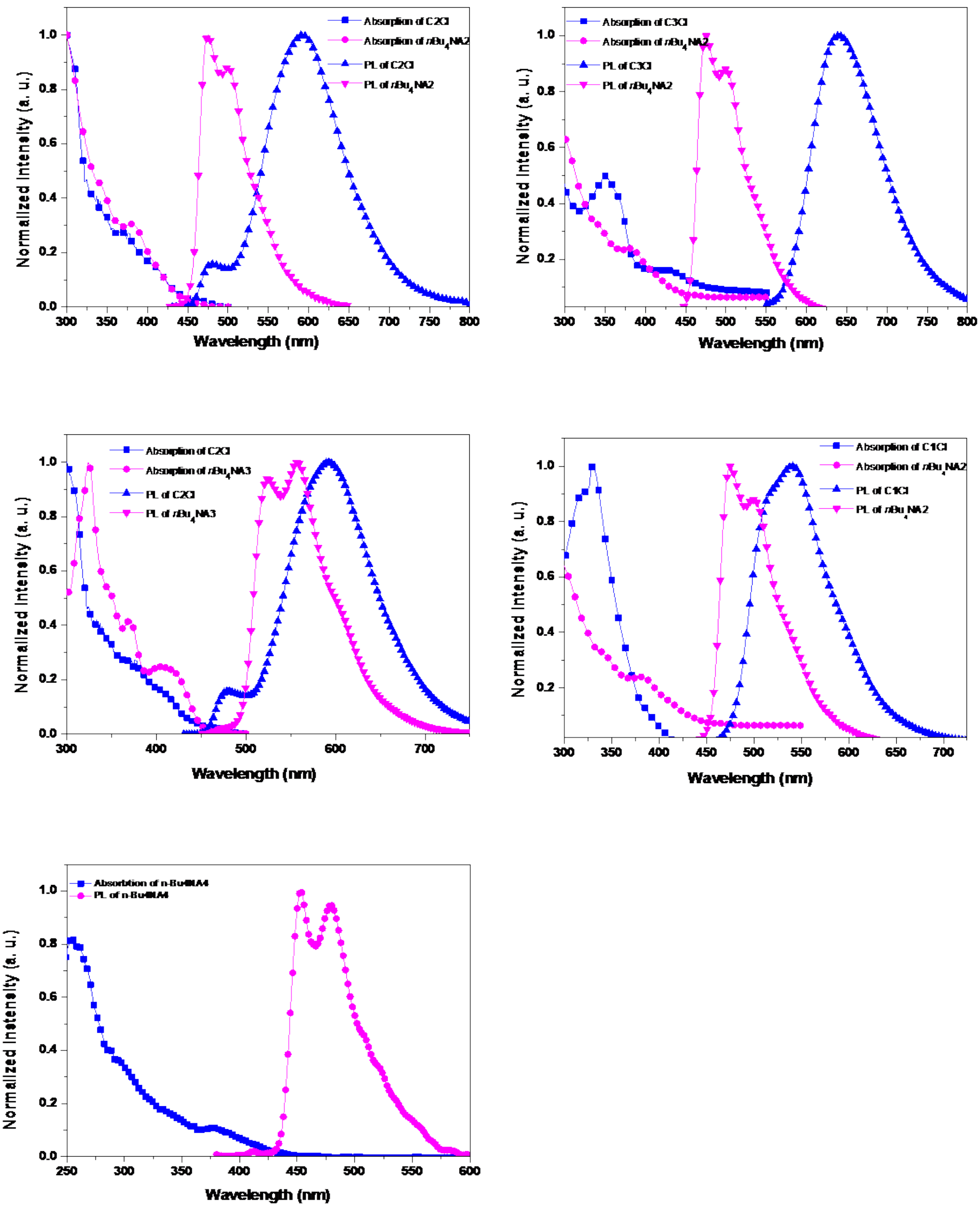

Fig. S3. Absorption and luminescence spectra of positive and negative complexes.

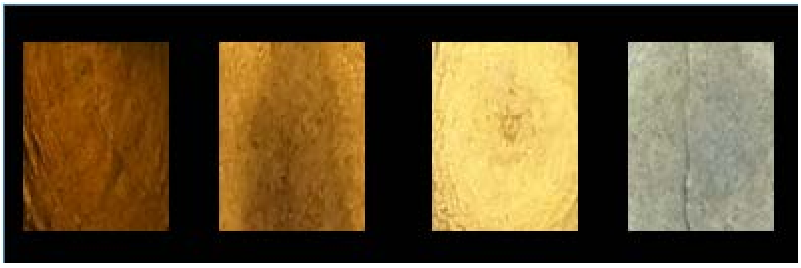

Fig. S4. Photographs of **IP6** doped in polymer at different contents under excitation at 365 nm, From left to right, the contents are 1.7 mg / 800 mg, 1.3 mg / 800 mg, 0.9 mg / 800 mg, 0.7 mg / 800 mg, respectively.

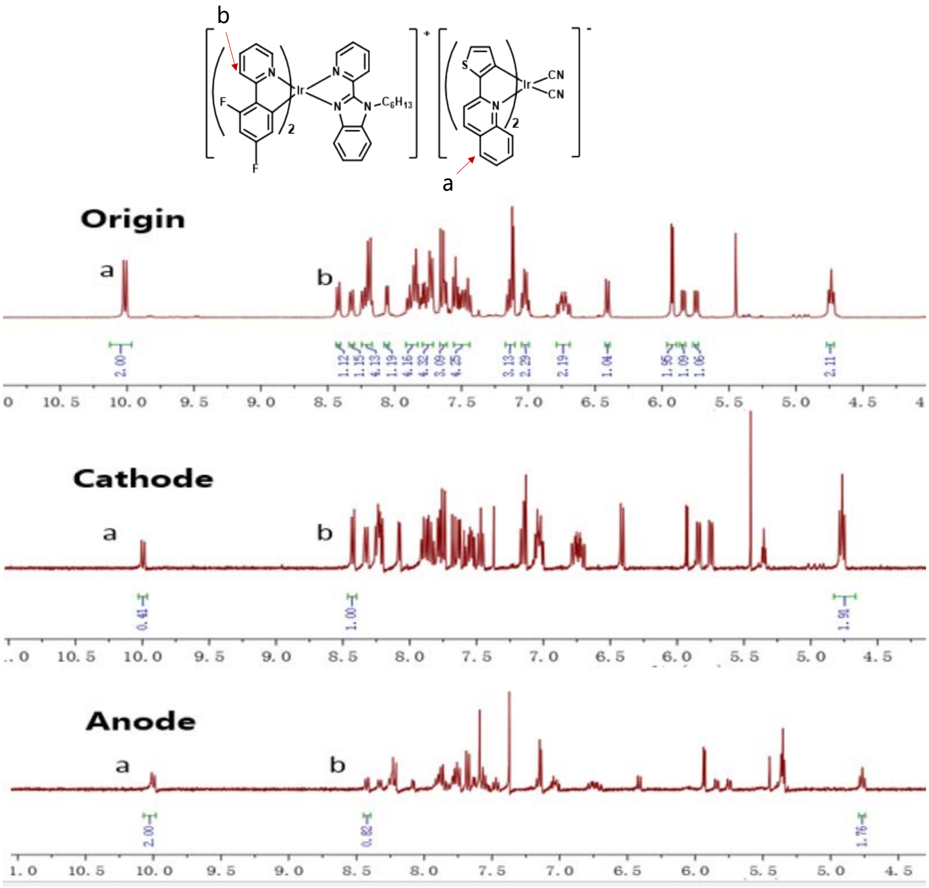

Fig. S5. The ^{1}H NMR spectra of **IP1** in acetonitrile-d_3 before and after applying a voltage of 3 V at anode and cathode.

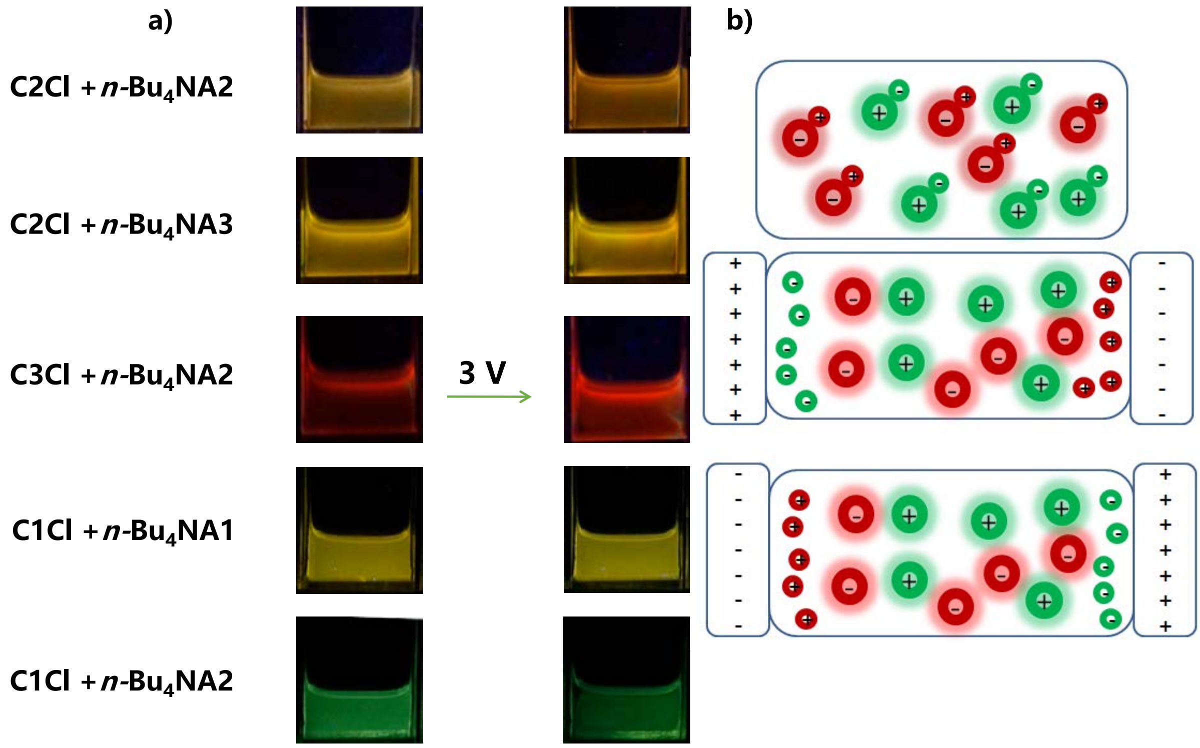

Fig. S6. (a) Photographs of the mixture of positive and negative complexes (equal molar quantity) in acetonitrile (10 μM) before (left) and after (right) applying a voltage of 3 V under excitation at 365 nm. (b) The mechanism diagram to explain the phenomenon of (a).

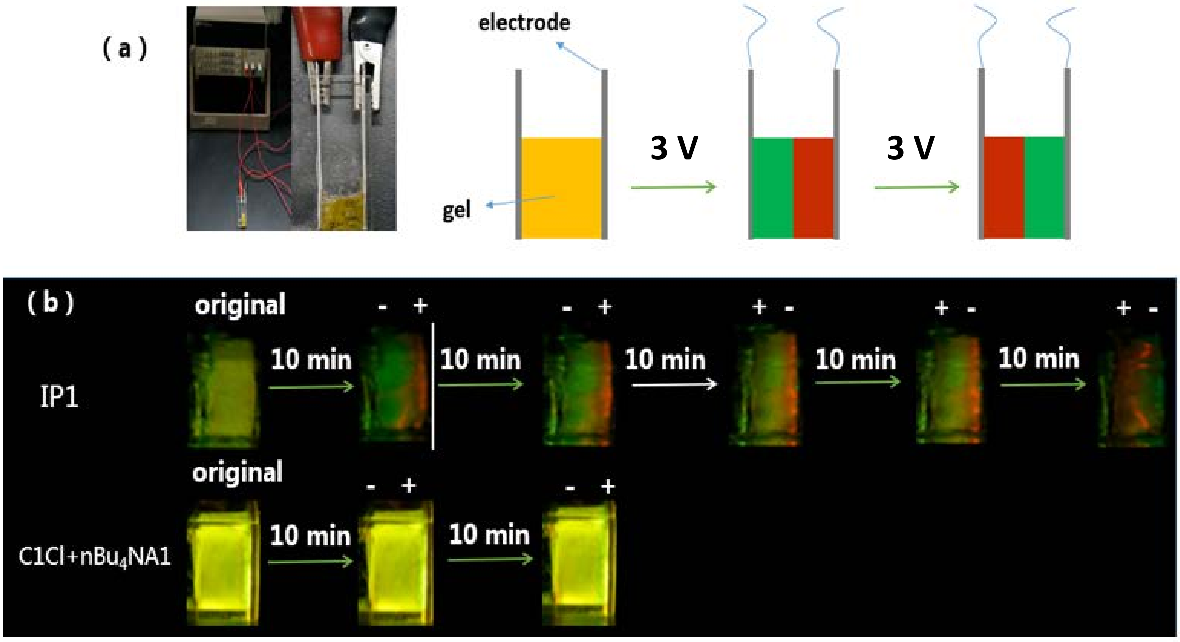

Fig. S7. (a) The schematic diagram of the setup for electrochromic luminescence experiments. (b) Photographs of electrochromic luminescence experiments using quasi-solid film doped with **IP1**.

The ^{1}H NMR, ^{13}C NMR, ^{19}F NMR and MS spectra of the compounds.

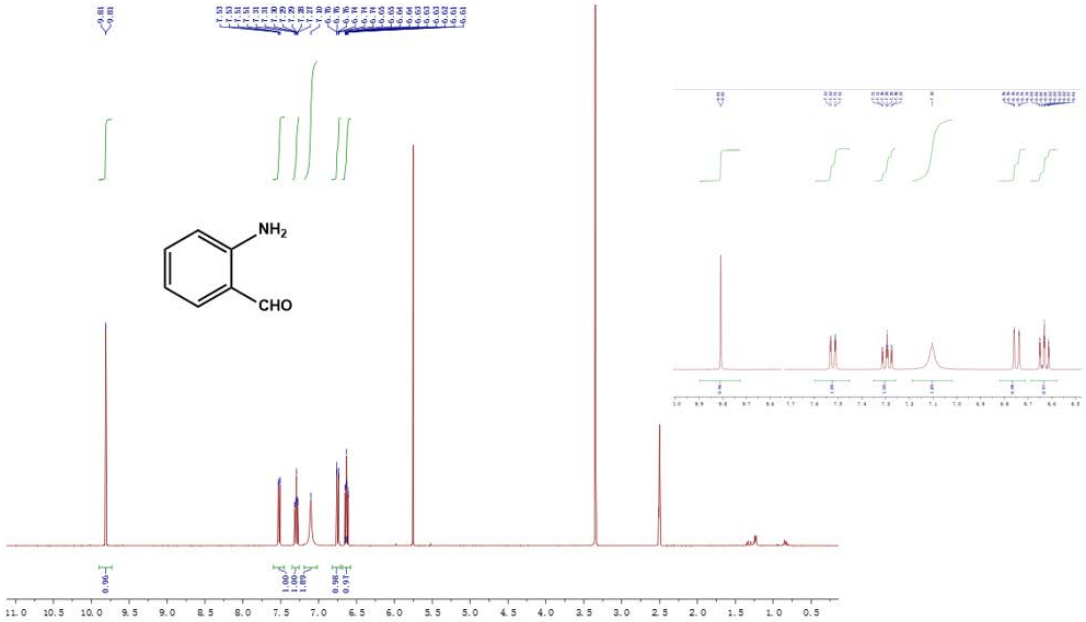

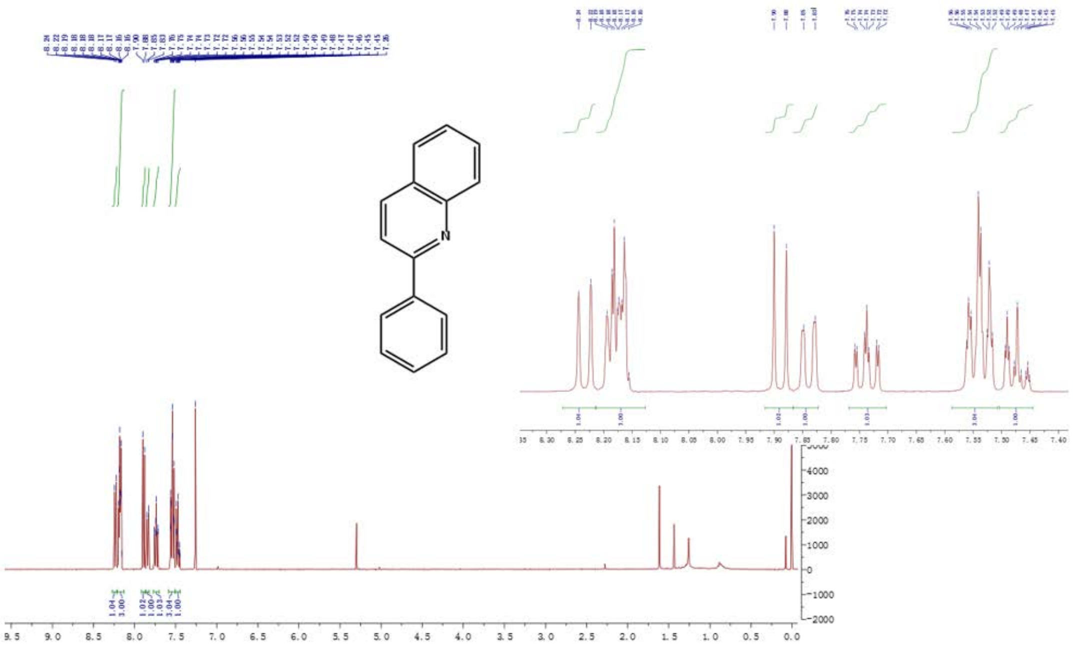
N
9.5 9.0 8.5 8.0 7.5 7.0 6.5 6.0 5.5 5.0 4.5 4.0 3.5 3.0 2.5 2.0 1.5 1.0 0.5 0.0
4000
3000
2000
1000
0
-1000
-2000
1.04
3.00
1.02
1.00
1.03
3.04
1.00

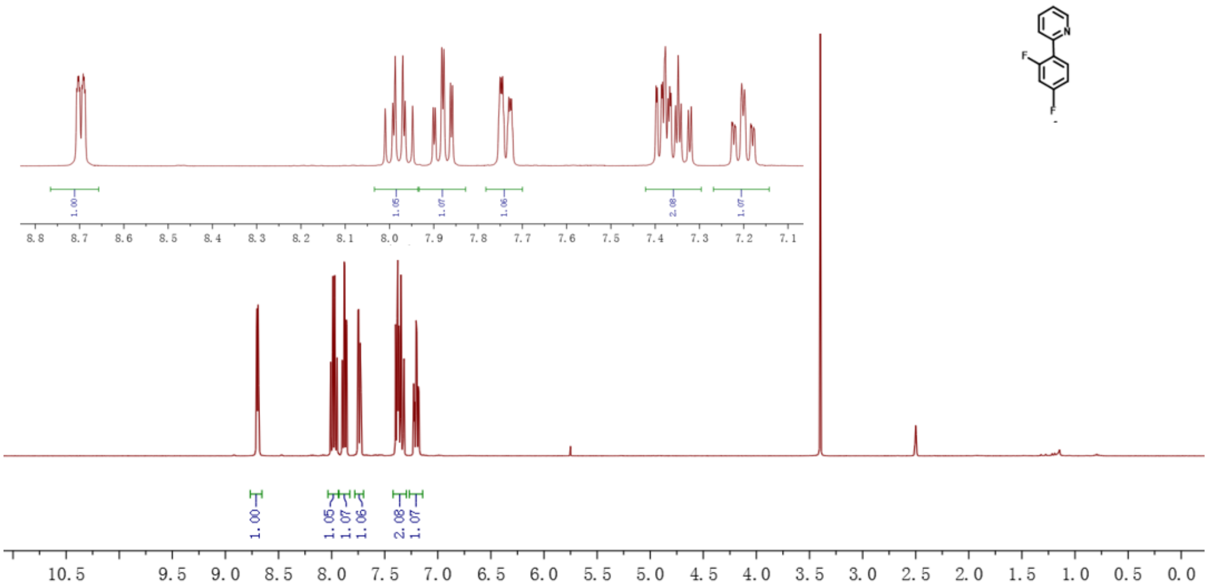

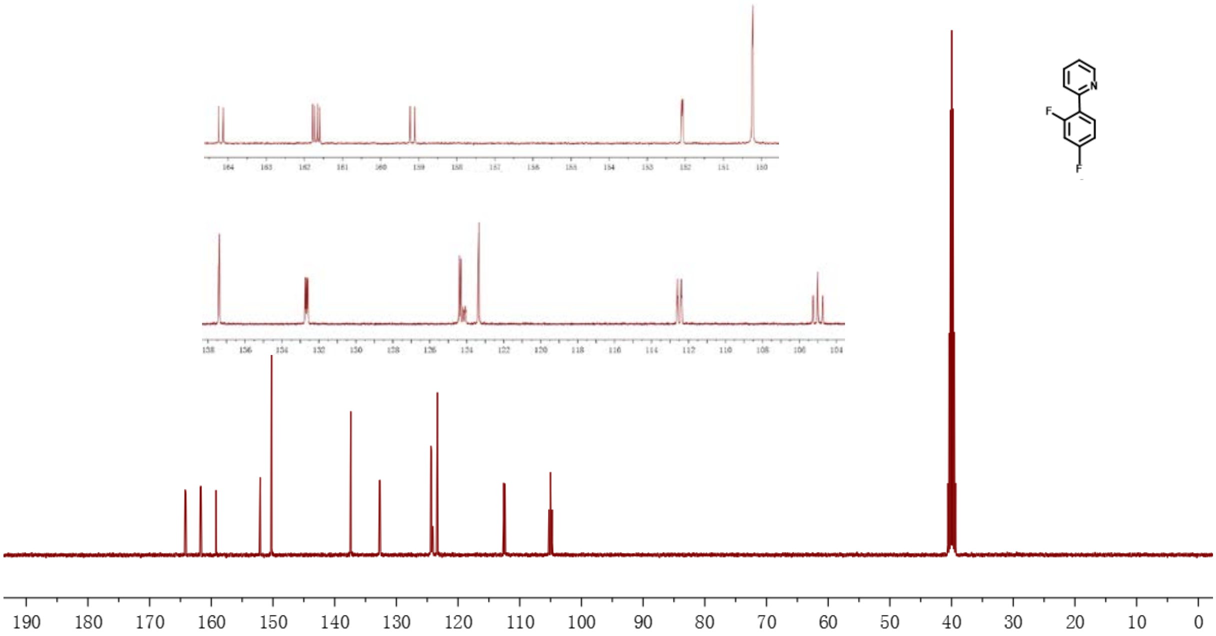
190
180
170
160
150
140
130
120
110
100
90
80
70
60
50
40
30
20
10
0

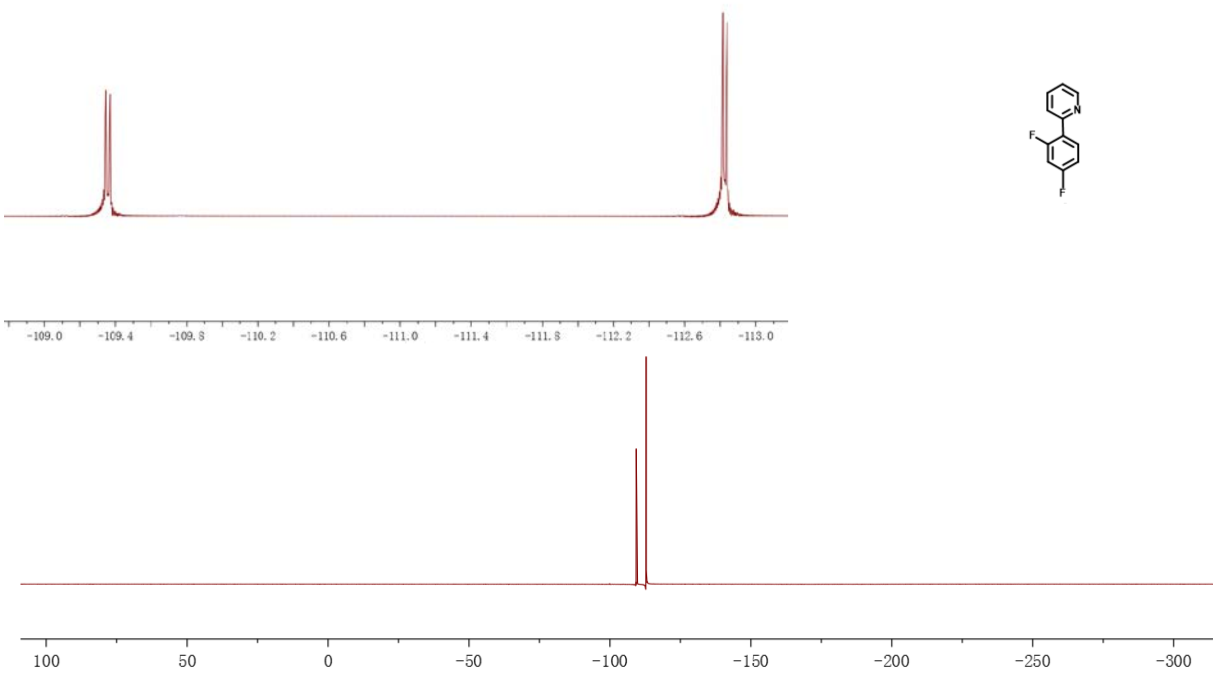

-109.0
-109.4
-109.8
-110.2
-110.6
-111.0
-111.4
-111.8
-112.2
-112.6
-113.0
F
N
F
100
50
0
−50
−100
−150
−200
−250
−300

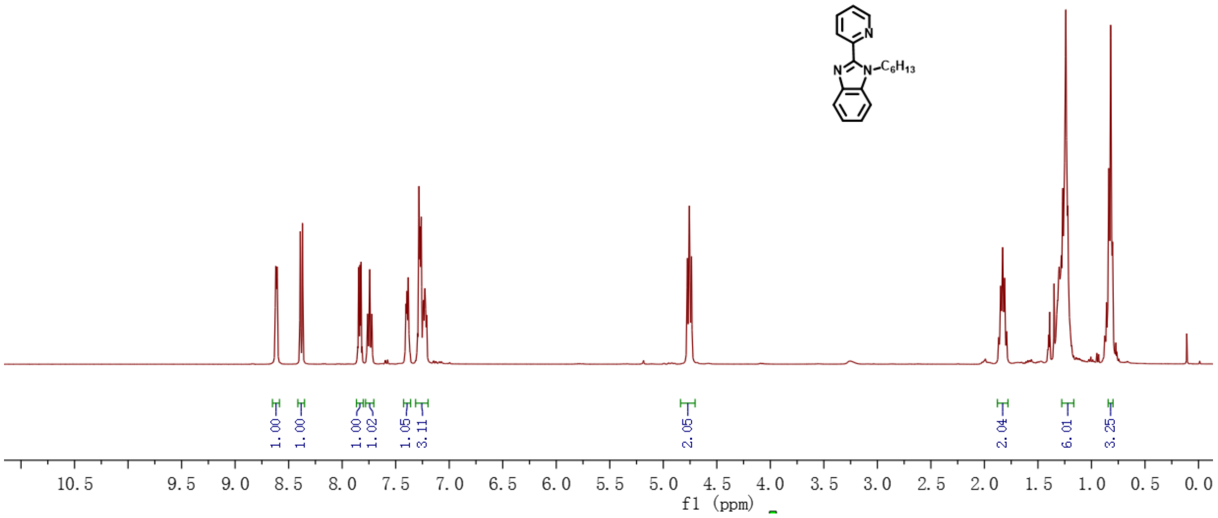
N
N
N
C6H13
1.00
1.00
1.00
1.02
1.05
3.11
2.05
2.04
6.01
3.25
10.5
9.5
9.0
8.5
8.0
7.5
7.0
6.5
6.0
5.5
5.0
4.5
4.0
3.5
3.0
2.5
2.0
1.5
1.0
0.5
0.0
f1 (ppm)

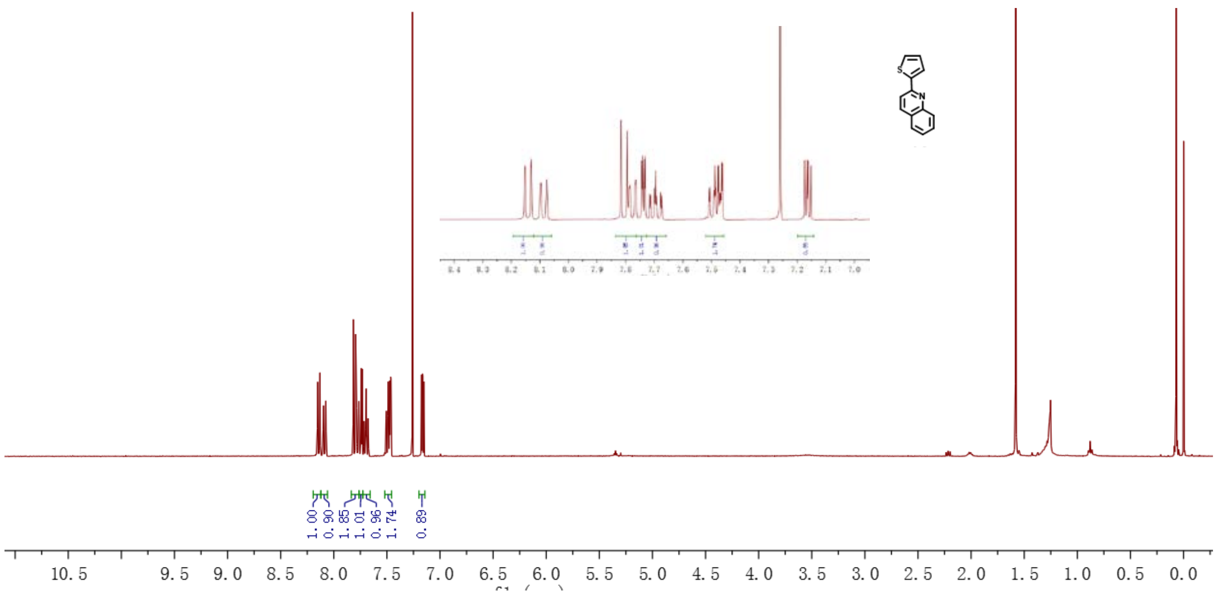

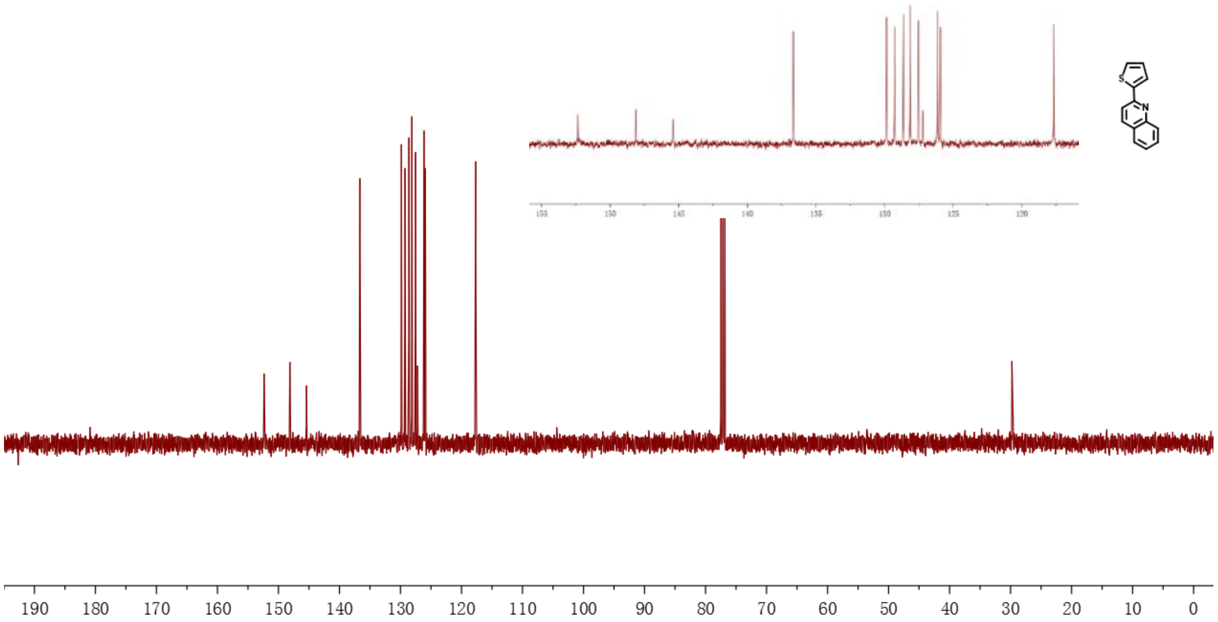
190 180 170 160 150 140 130 120 110 100 90 80 70 60 50 40 30 20 10 0

C1Cl

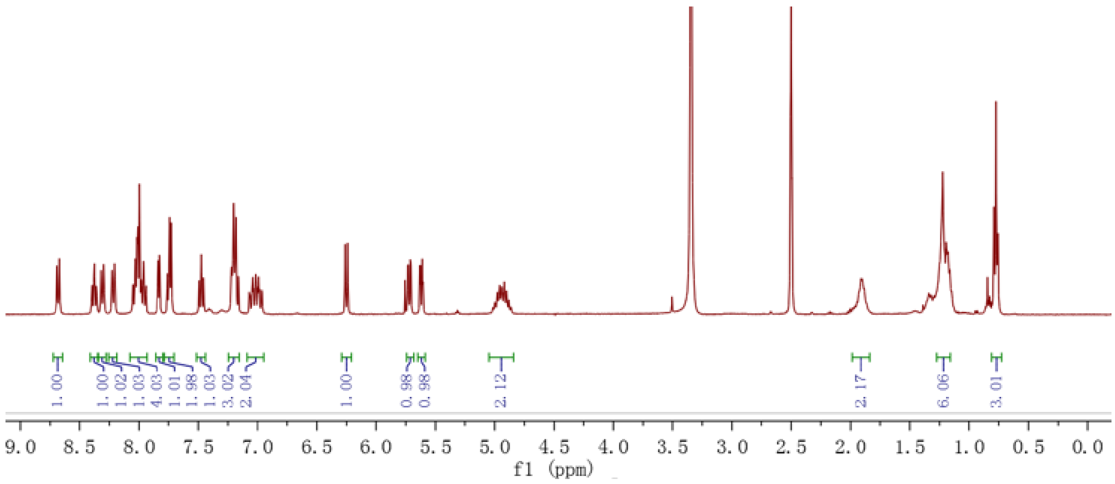

C1Cl

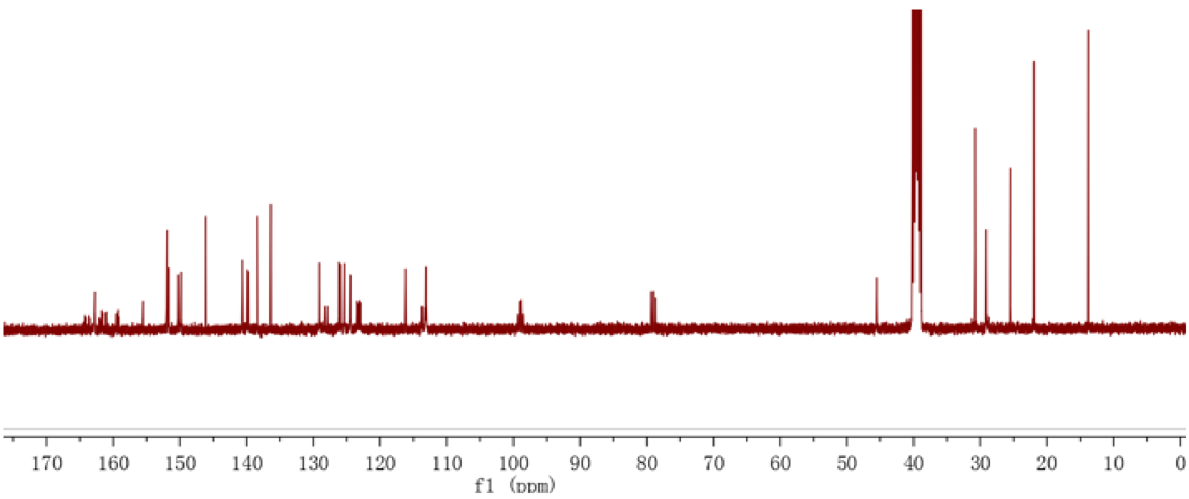

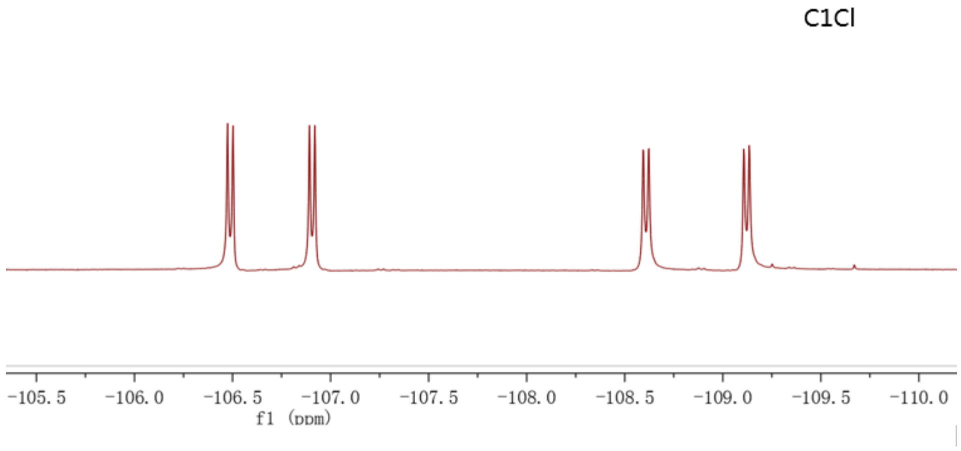

$^{19}F\{^{1}H\}$NMR (377 M)

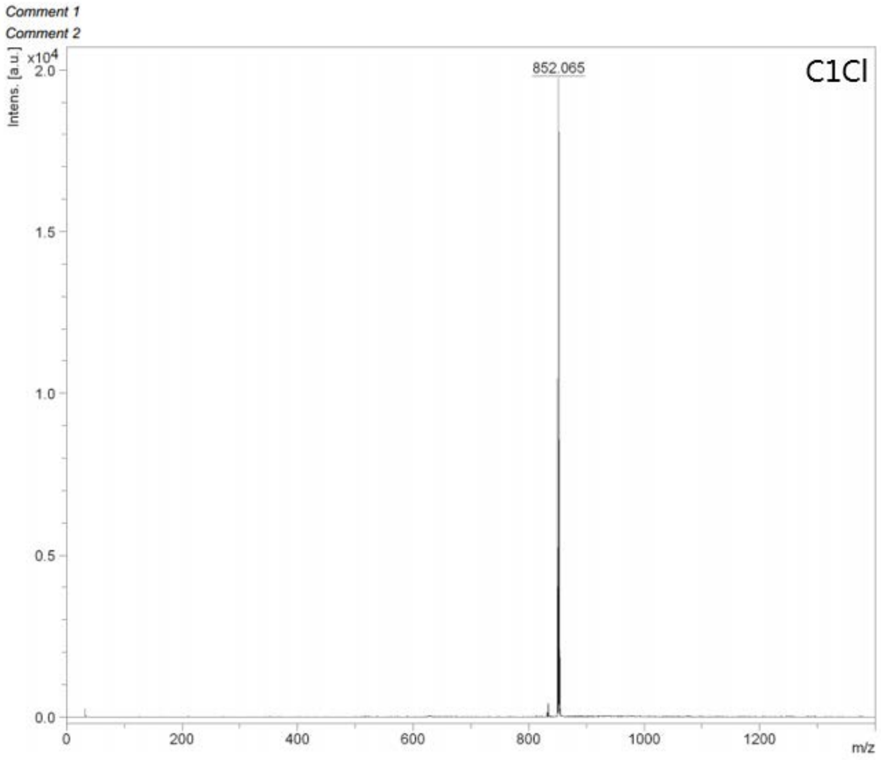
Comment 1
Comment 2
Intens. [a.u.]
x10^4
2.0
1.5
1.0
0.5
0.0
852.065
C1Cl
0
200
400
600
800
1000
1200
m/z

C2Cl

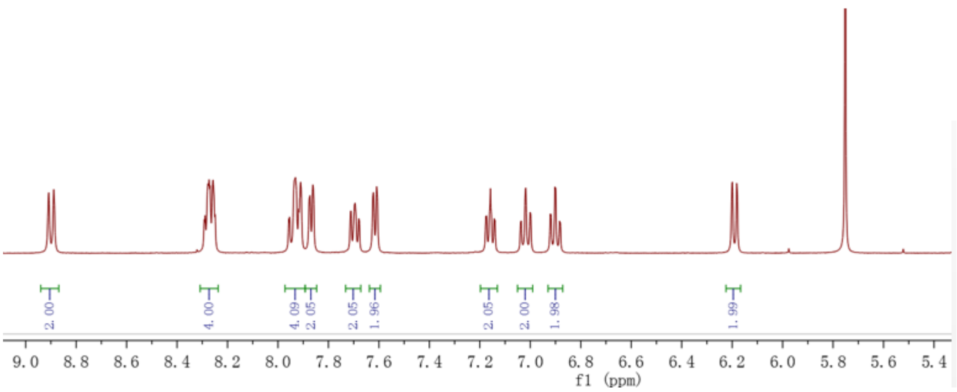
2.00
4.00
4.09
2.05
2.05
1.96
2.05
2.00
1.98
1.99
9.0 8.8 8.6 8.4 8.2 8.0 7.8 7.6 7.4 7.2 7.0 6.8 6.6 6.4 6.2 6.0 5.8 5.6 5.4
f1 (ppm)

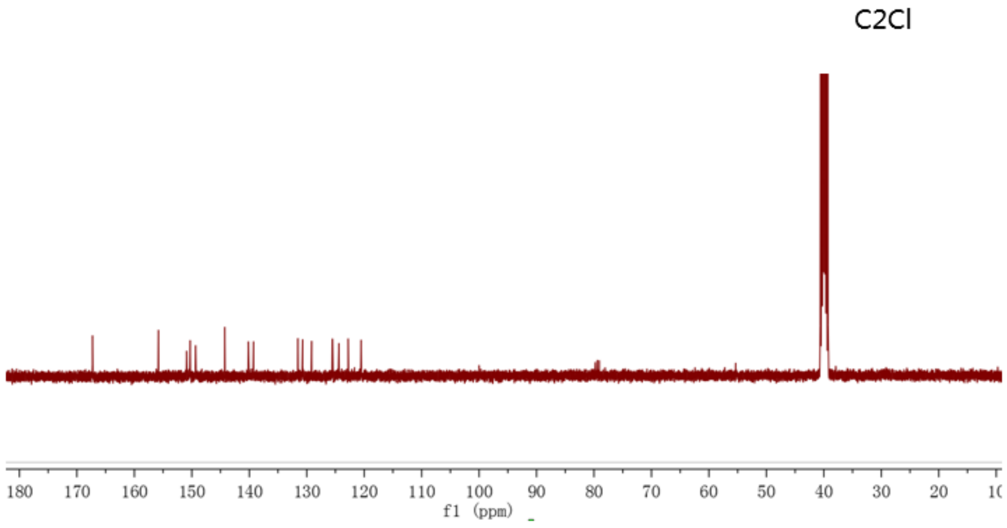
C2Cl
180
170
160
150
140
130
120
110
100
90
80
70
60
50
40
30
20
f1 (ppm)

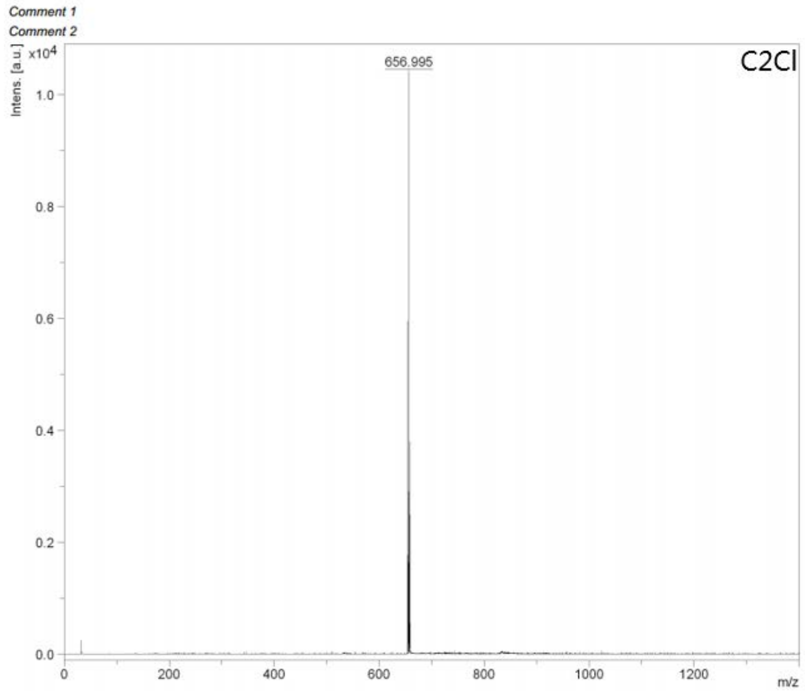
Comment 1
Comment 2
Intens. [a.u.]
x10^4
656.995
C2Cl
1.0
0.8
0.6
0.4
0.2
0.0
0
200
400
600
800
1000
1200
m/z

C3Cl

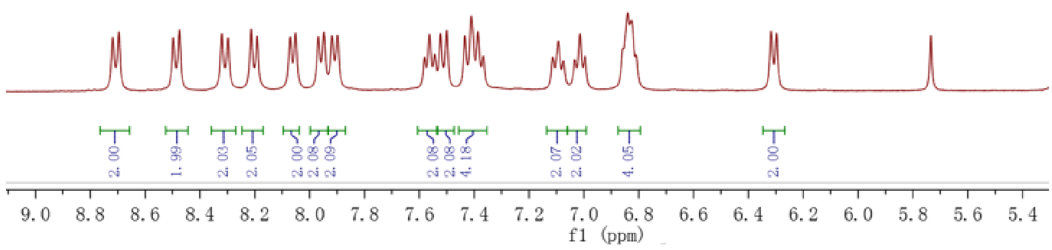
2.00
1.99
2.03
2.05
2.00
2.08
2.09
2.08
2.08
4.18
2.07
2.02
4.05
2.00
9.0 8.8 8.6 8.4 8.2 8.0 7.8 7.6 7.4 7.2 7.0 6.8 6.6 6.4 6.2 6.0 5.8 5.6 5.4
f1 (ppm)

C3Cl

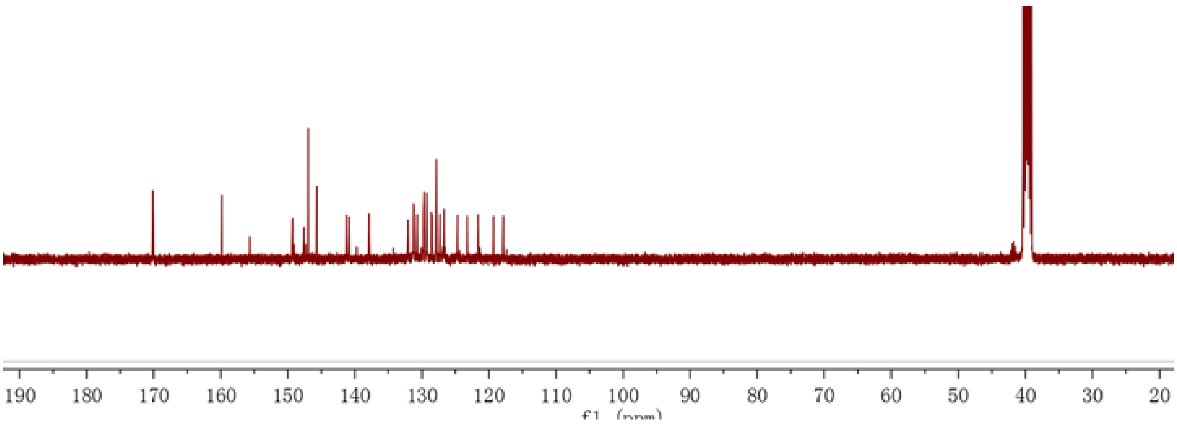
190 180 170 160 150 140 130 120 110 100 90 80 70 60 50 40 30 20
f1 (ppm)

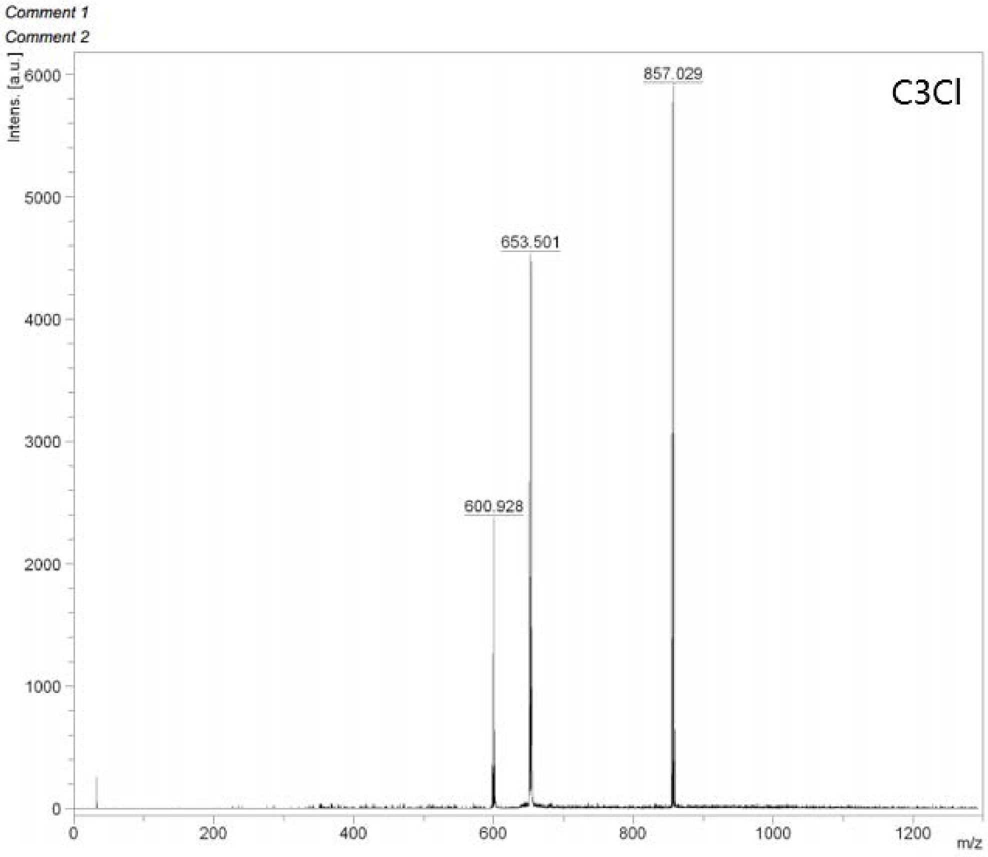
Comment 1
Comment 2
Intens. [a.u.]
6000
5000
4000
3000
2000
1000
0
857.029
C3Cl
653.501
600.928
0
200
400
600
800
1000
1200
m/z

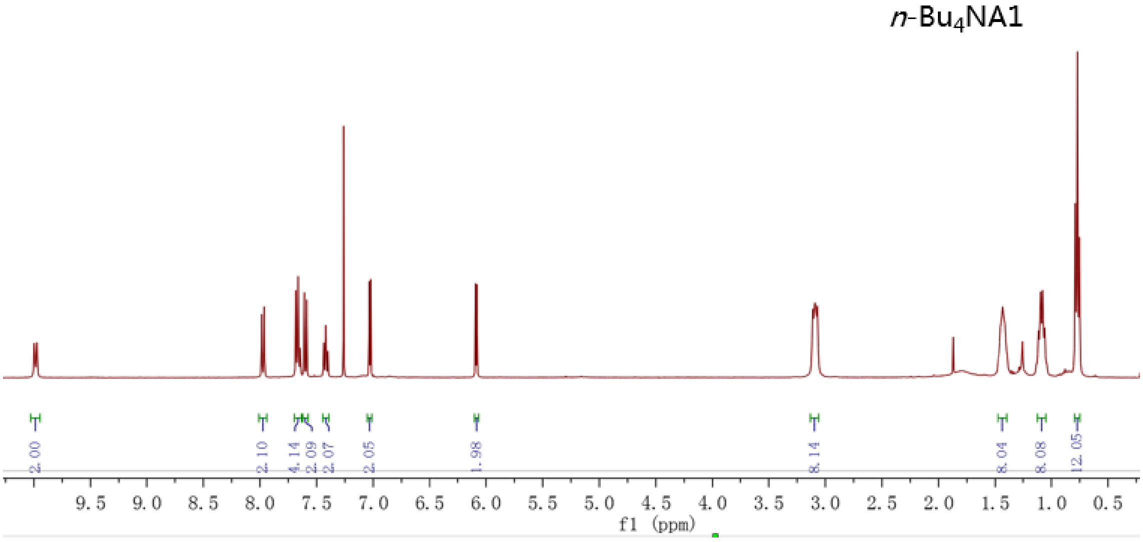
n-Bu4NA1
2.00
2.10
4.14
2.09
2.07
2.05
1.98
8.14
8.04
8.08
12.05
9.5 9.0 8.5 8.0 7.5 7.0 6.5 6.0 5.5 5.0 4.5 4.0 3.5 3.0 2.5 2.0 1.5 1.0 0.5
f1 (ppm)

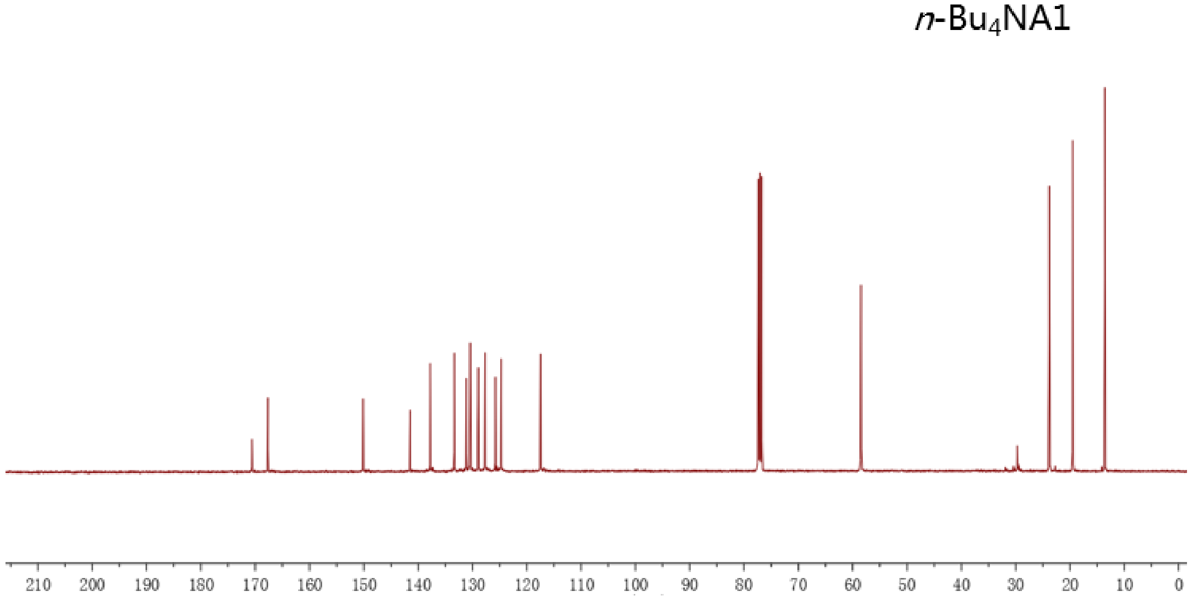
n-Bu4NA1
210 200 190 180 170 160 150 140 130 120 110 100 90 80 70 60 50 40 30 20 10 0

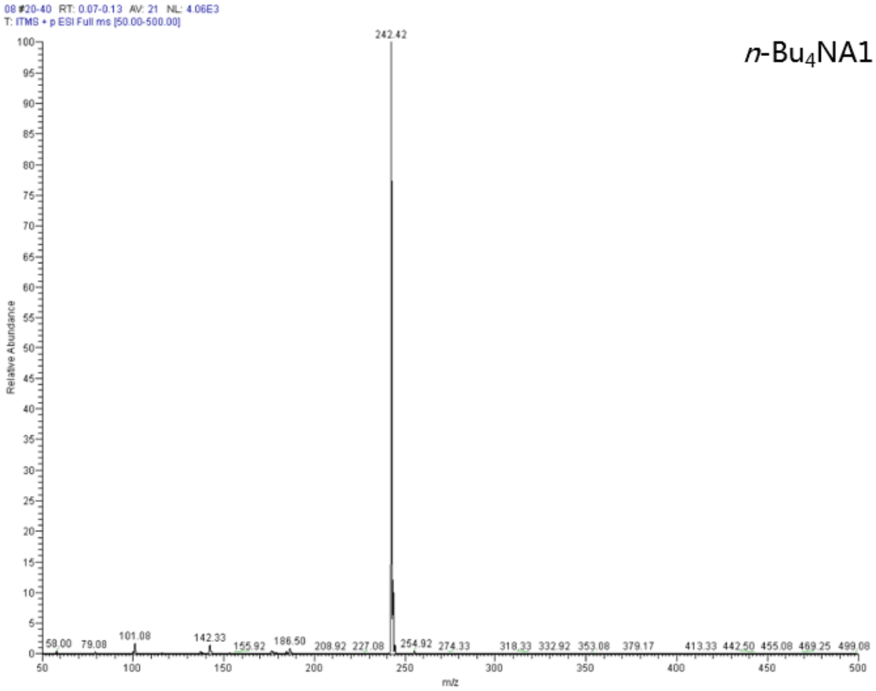
08 #20-40 RT: 0.07-0.13 AV: 21 NL: 4.06E3
T: ITMS + p ESI Full ms [50.00-500.00]
242.42
Relative Abundance
m/z
58.00
79.08
101.08
142.33
155.92
186.50
208.92
227.08
254.92
274.33
318.33
332.92
353.08
379.17
413.33
442.50
455.08
469.25
499.08

n-Bu_4NA1

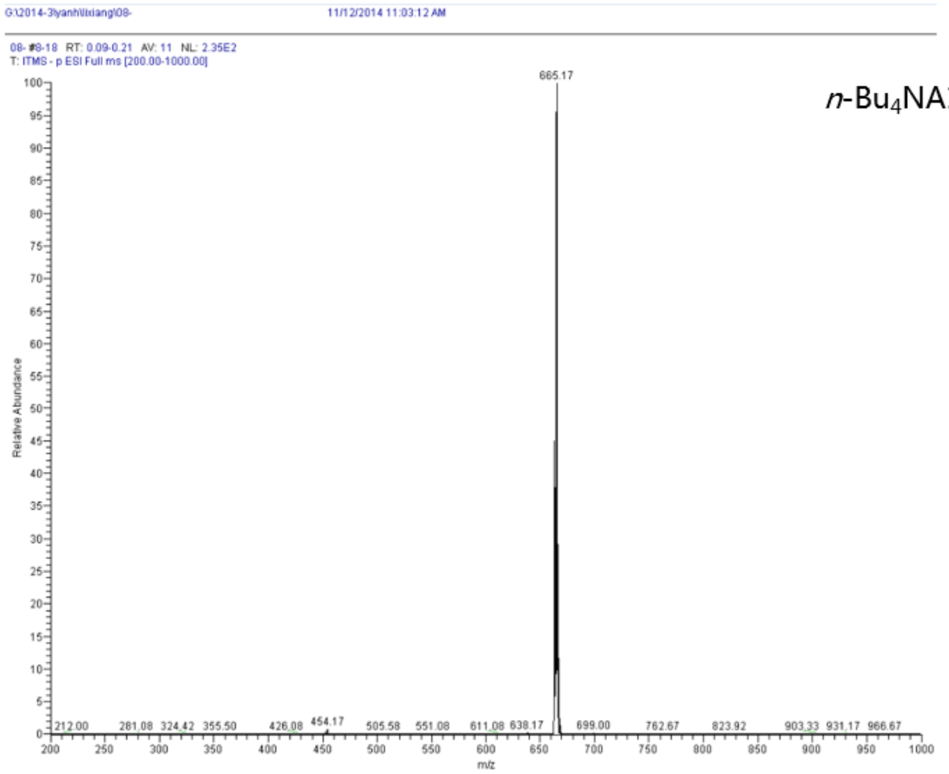
G:\2014-3\yanh\lixiang\08-
11/12/2014 11:03:12 AM
08- #8-18 RT: 0.09-0.21 AV: 11 NL: 2.35E2
T: ITMS - p ESI Full ms [200.00-1000.00]
665.17
Relative Abundance
212.00
281.08
324.42
355.50
426.08
454.17
505.58
551.08
611.08
638.17
699.00
762.67
823.92
903.33
931.17
966.67
m/z

n-Bu_4NA1

n-Bu_4NA2

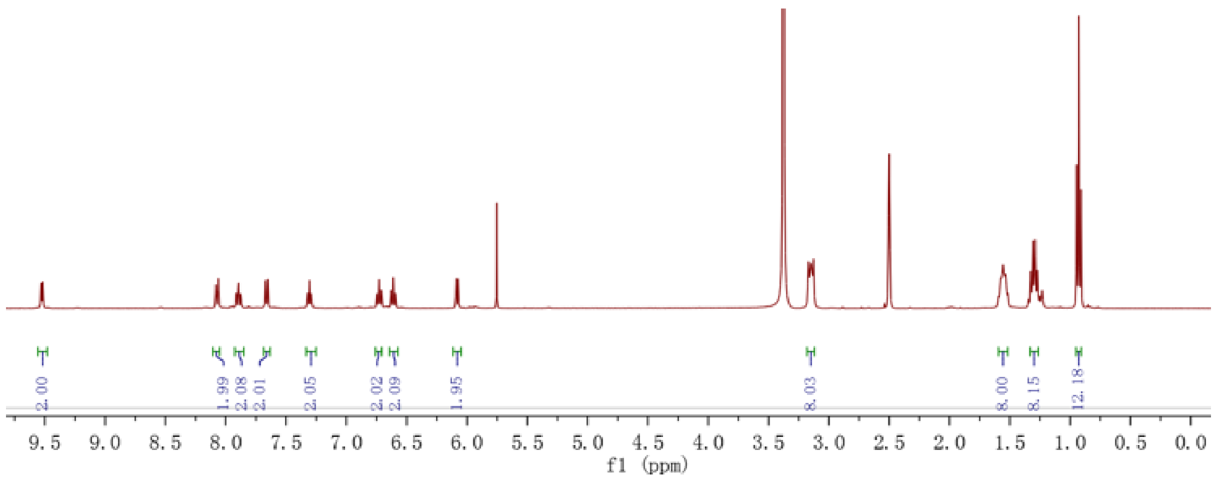
2.00
1.99
2.08
2.01
2.05
2.02
2.09
1.95
8.03
8.00
8.15
12.18
9.5 9.0 8.5 8.0 7.5 7.0 6.5 6.0 5.5 5.0 4.5 4.0 3.5 3.0 2.5 2.0 1.5 1.0 0.5 0.0
f1 (ppm)

n-Bu_4NA2

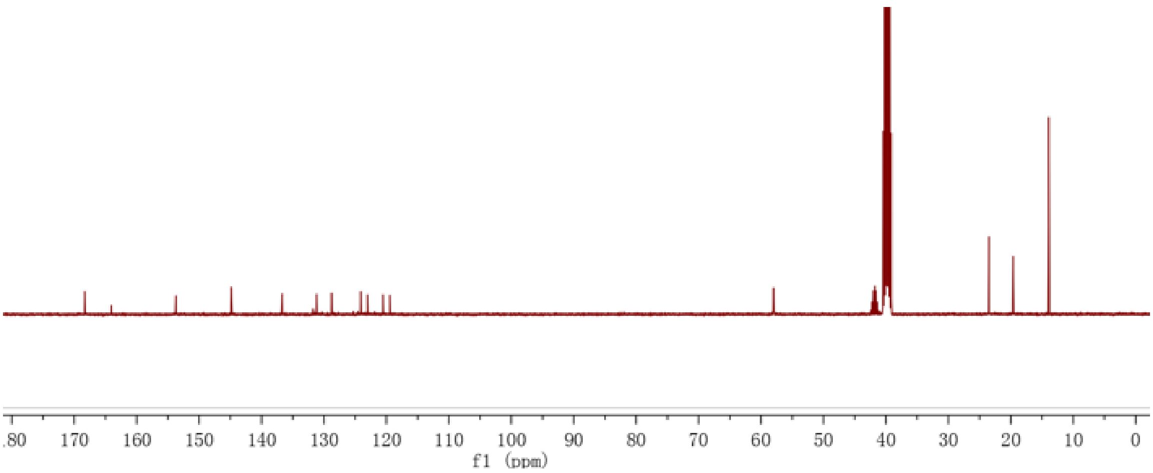

180
170
160
150
140
130
120
110
100
90
80
70
60
50
40
30
20
10
0
f1 (ppm)

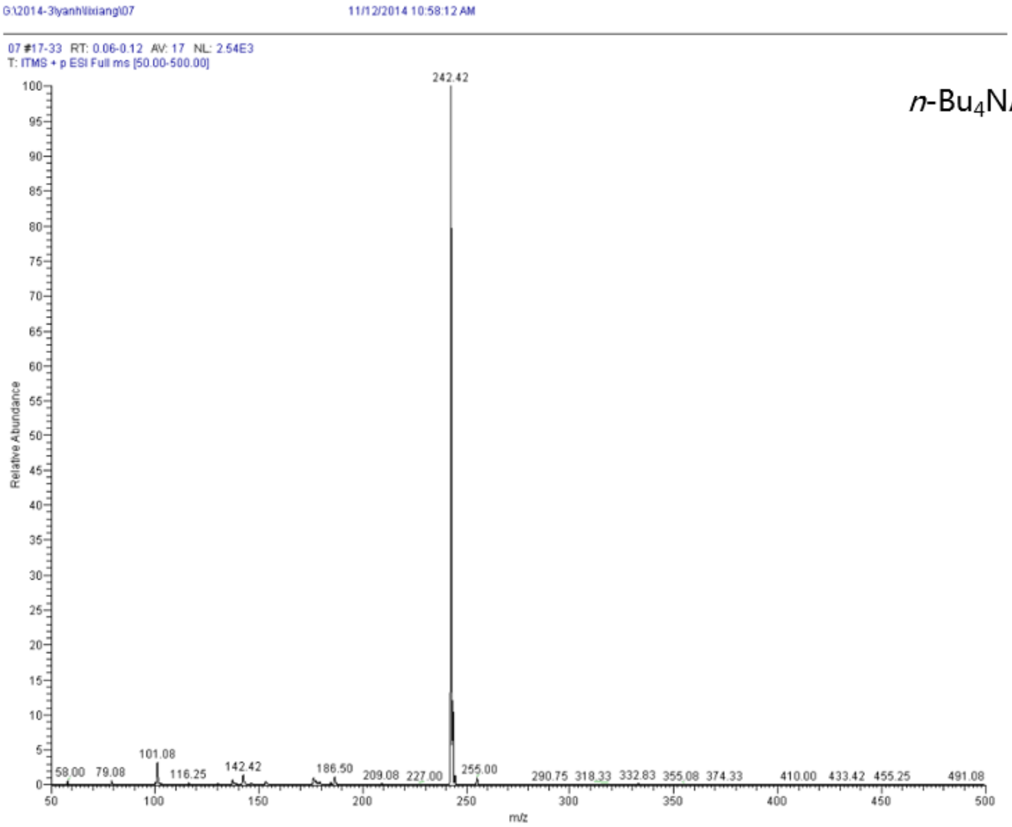
G:\2014-3\yanh\lixiang\07
11/12/2014 10:58:12 AM
07 #17-33 RT: 0.06-0.12 AV: 17 NL: 2.54E3
T: ITMS + p ESI Full ms [50.00-500.00]
242.42
Relative Abundance
101.08
142.42
186.50
255.00
m/z

n-Bu_4NA2

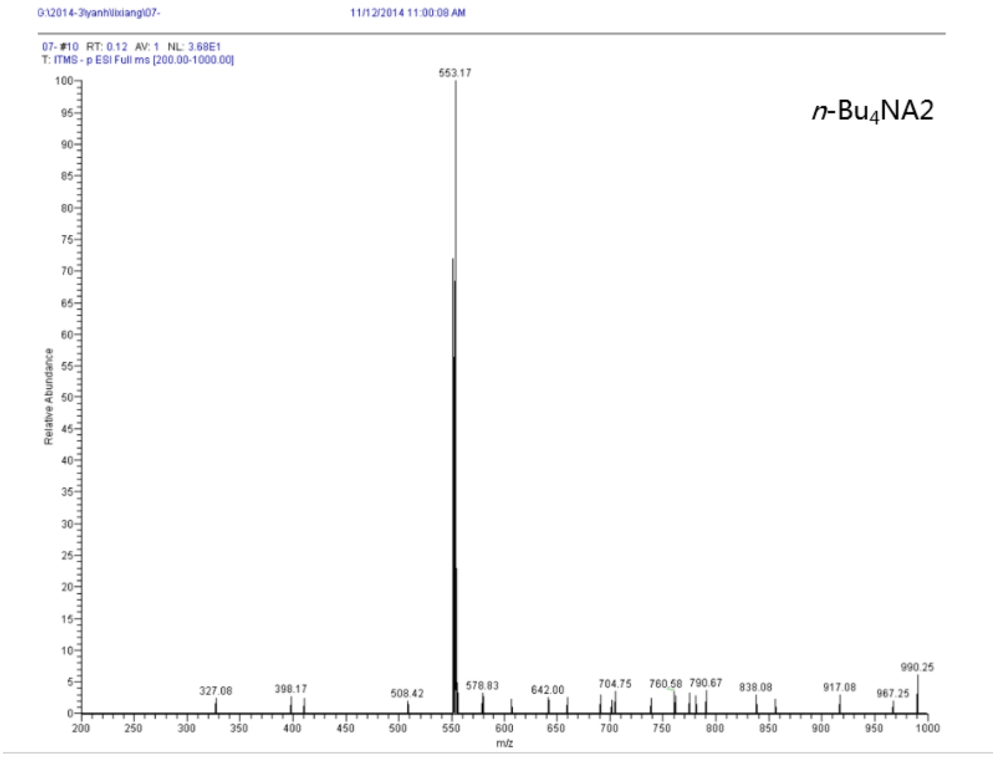
G:\2014-3\yanh\lixiang\07-
11/12/2014 11:00:08 AM
07- #10 RT: 0.12 AV: 1 NL: 3.68E1
T: ITMS - p ESI Full ms [200.00-1000.00]
n-Bu4NA2
553.17
327.08
398.17
508.42
578.83
642.00
704.75
760.58
790.67
838.08
917.08
967.25
990.25
Relative Abundance
m/z

n-Bu_4NA3

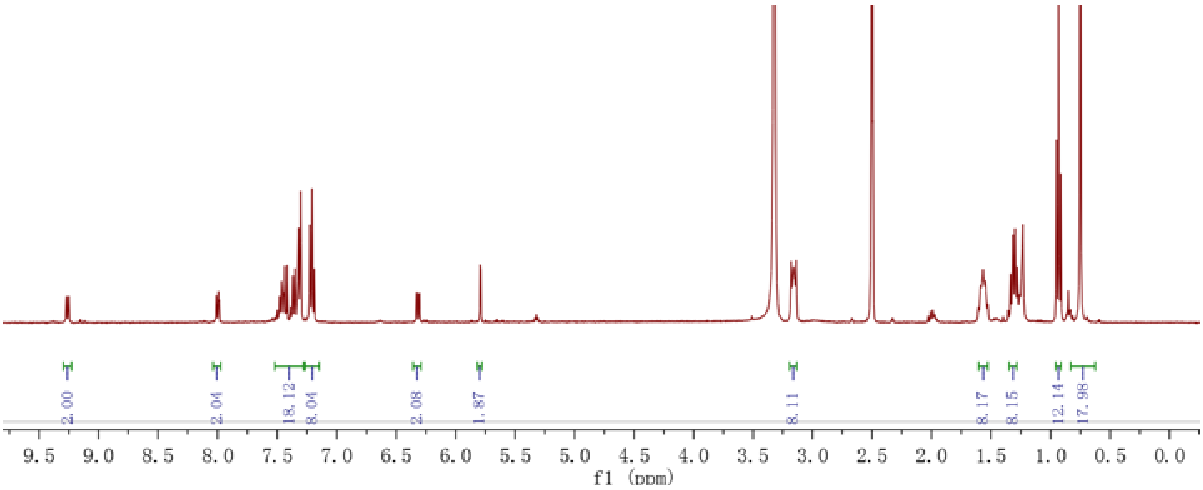
2.00
2.04
18.12
8.04
2.08
1.87
8.11
8.17
8.15
12.14
17.98
9.5 9.0 8.5 8.0 7.5 7.0 6.5 6.0 5.5 5.0 4.5 4.0 3.5 3.0 2.5 2.0 1.5 1.0 0.5 0.0
f1 (ppm)

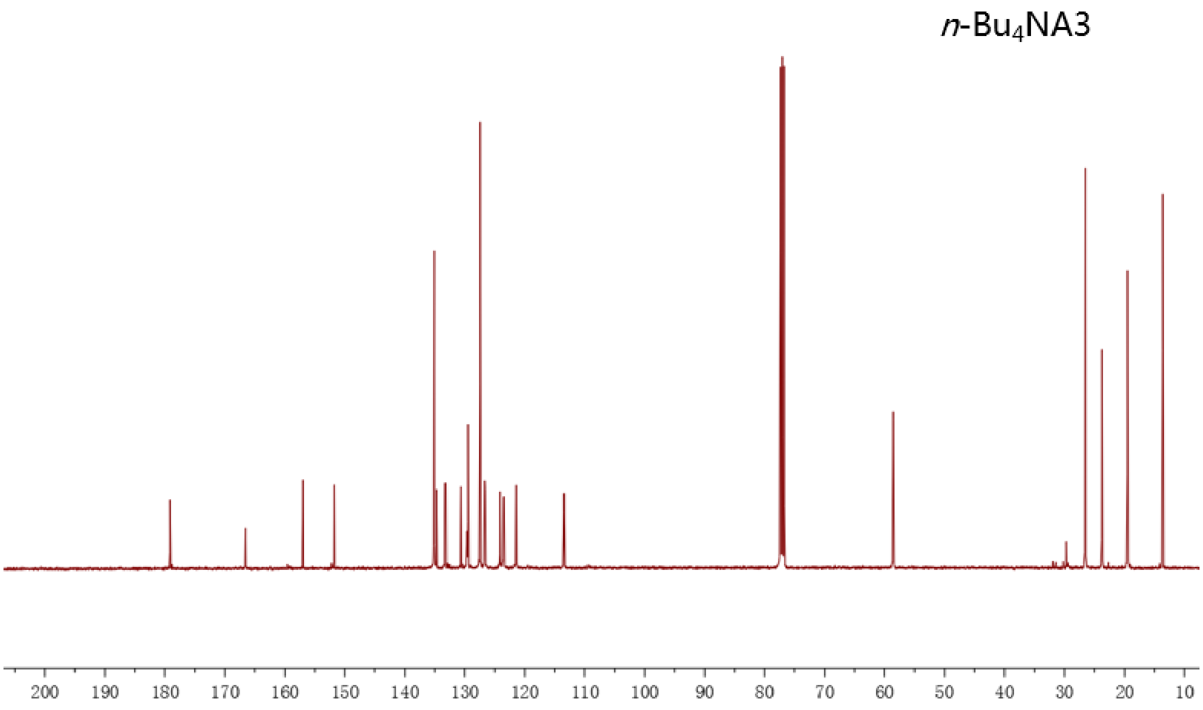
n-Bu4NA3
200
190
180
170
160
150
140
130
120
110
100
90
80
70
60
50
40
30
20
10

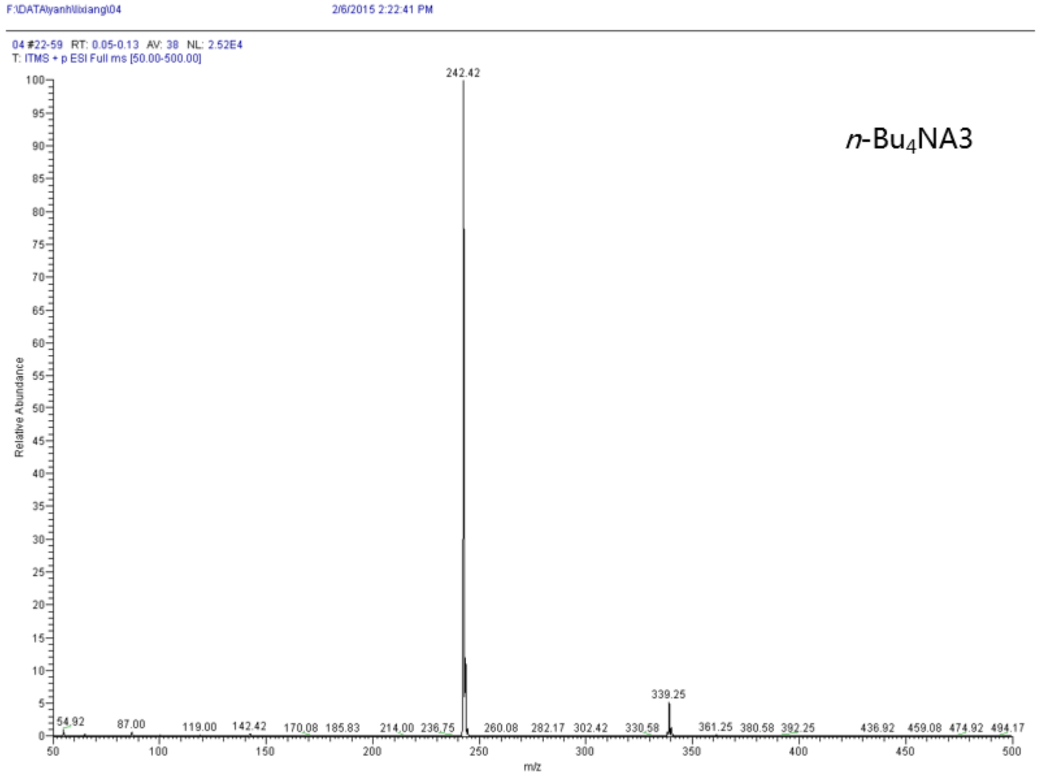
F:\DATA\yanh\lixiang\04
2/6/2015 2:22:41 PM
04 #22-59 RT: 0.05-0.13 AV: 38 NL: 2.52E4
T: ITMS + p ESI Full ms [50.00-500.00]
242.42
n-Bu₄NA3
Relative Abundance
339.25
54.92
87.00
119.00
142.42
170.08
185.83
214.00
236.75
260.08
282.17
302.42
330.58
361.25
380.58
392.25
436.92
459.08
474.92
494.17
m/z

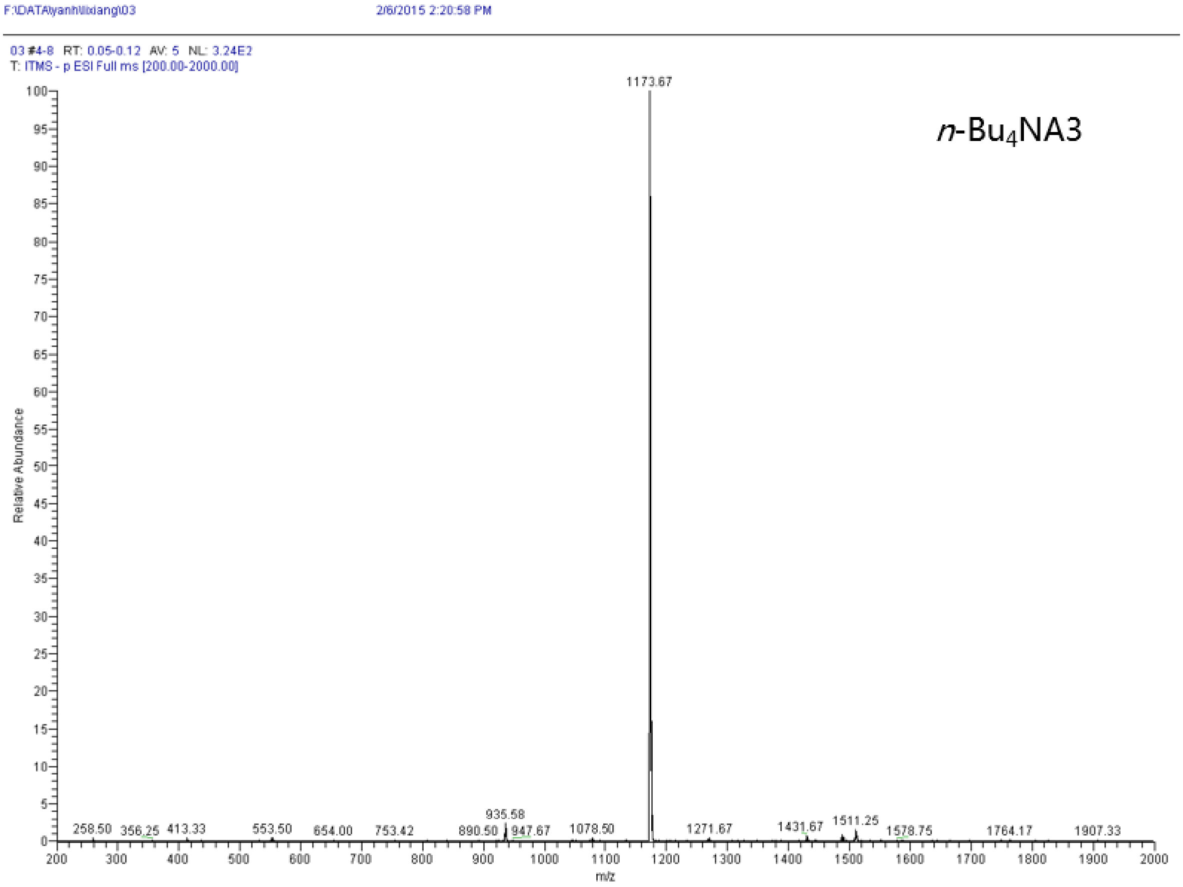
F:\DATA\yanhl\lixiang\03
2/6/2015 2:20:58 PM
03 #4-8 RT: 0.05-0.12 AV: 5 NL: 3.24E2
T: ITMS - p ESI Full ms [200.00-2000.00]
1173.67
n-Bu4NA3
Relative Abundance
m/z
258.50
356.25
413.33
553.50
654.00
753.42
890.50
935.58
947.67
1078.50
1271.67
1431.67
1511.25
1578.75
1764.17
1907.33

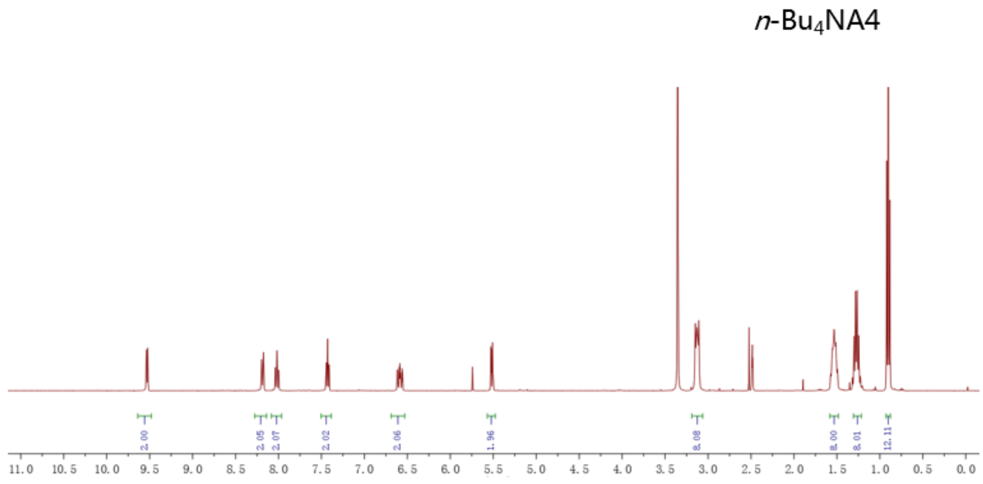
n-Bu4NA4
2.00
2.05
2.07
2.02
2.06
1.96
8.08
8.00
8.01
12.11
11.0 10.5 10.0 9.5 9.0 8.5 8.0 7.5 7.0 6.5 6.0 5.5 5.0 4.5 4.0 3.5 3.0 2.5 2.0 1.5 1.0 0.5 0.0

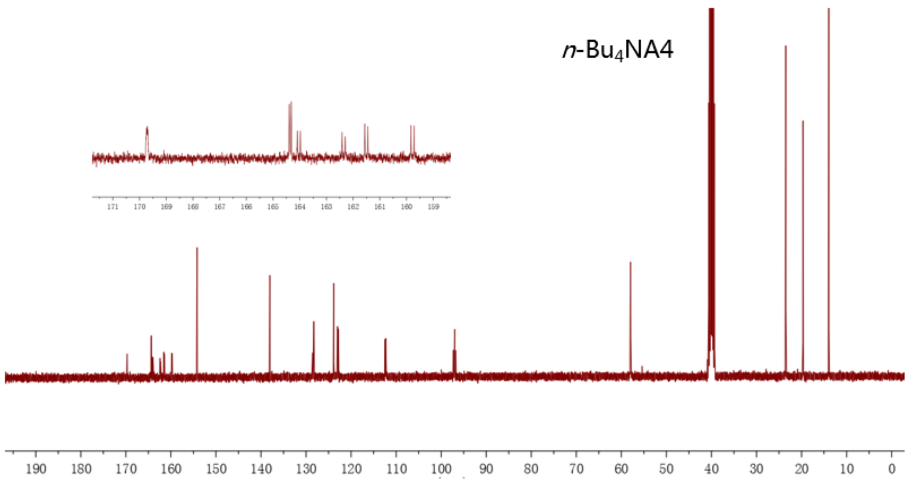

n-Bu4NA4
171 170 169 168 167 166 165 164 163 162 161 160 159
190 180 170 160 150 140 130 120 110 100 90 80 70 60 50 40 30 20 10 0

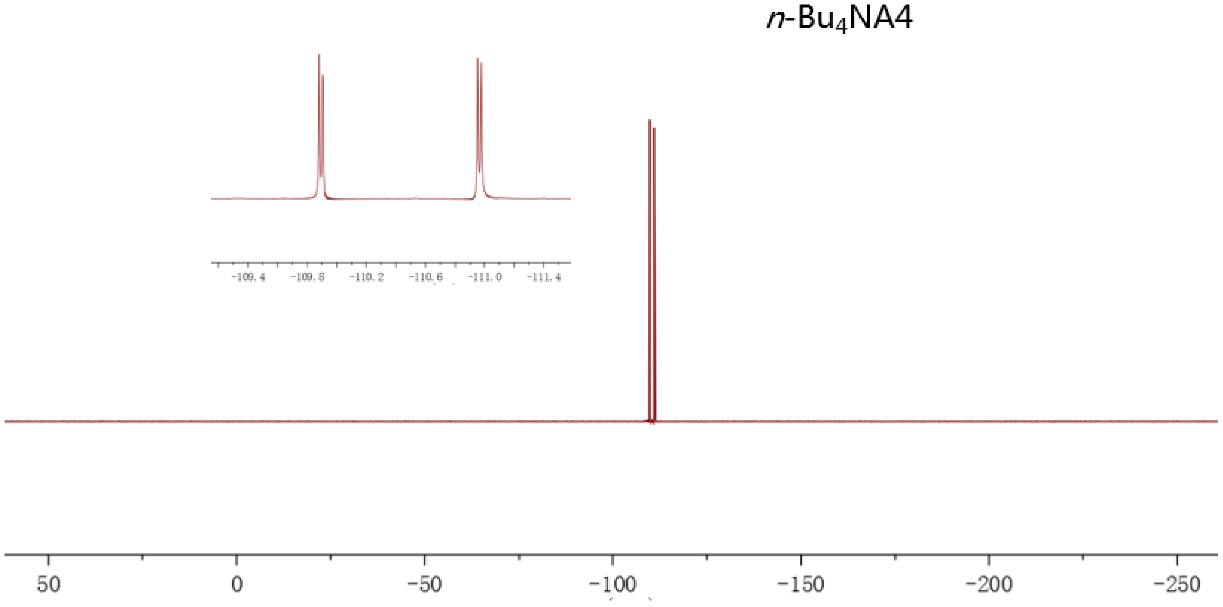

$^{19}F\{^{1}H\}$ NMR (377 M)

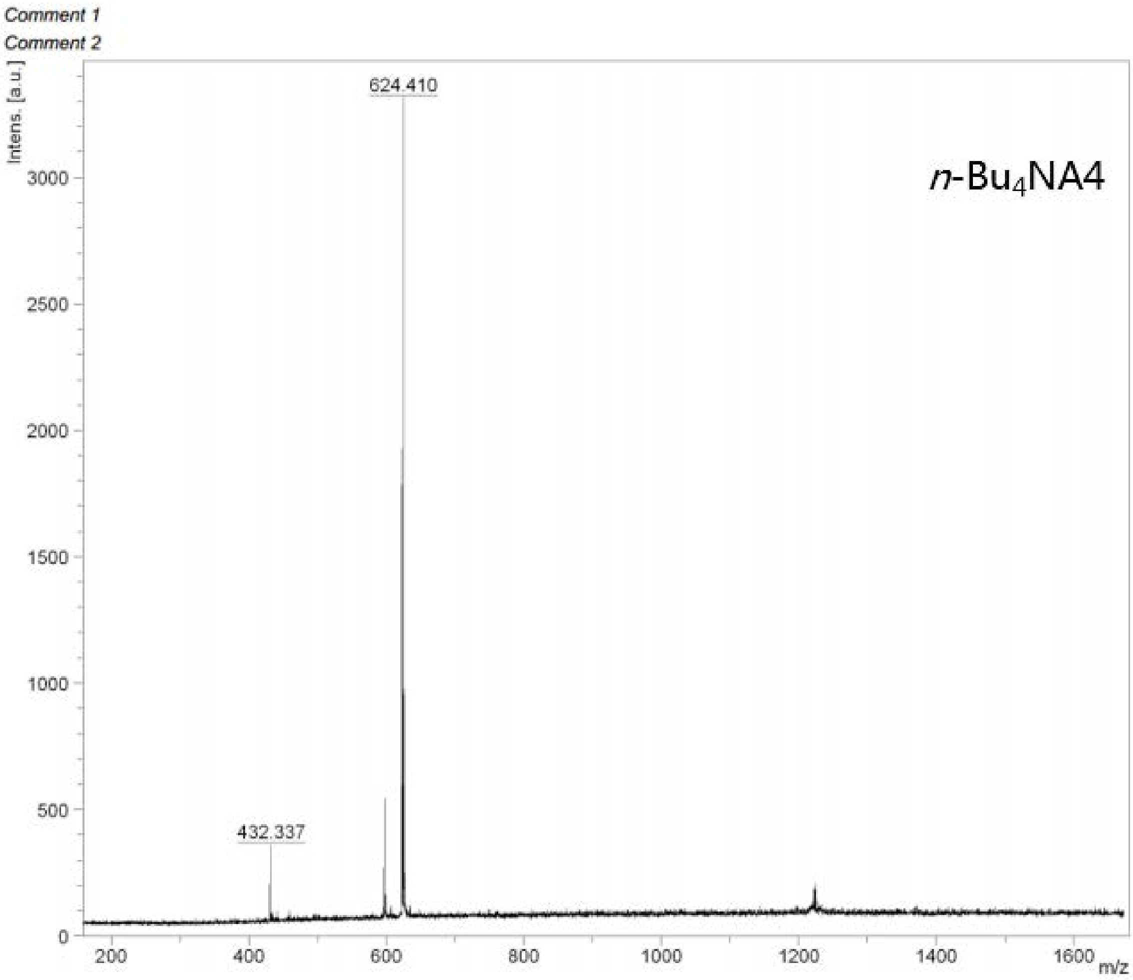
Comment 1
Comment 2
Intens. [a.u.]
624.410
432.337
n-Bu4NA4
m/z
3000
2500
2000
1500
1000
500
0
200
400
600
800
1000
1200
1400
1600

IP1

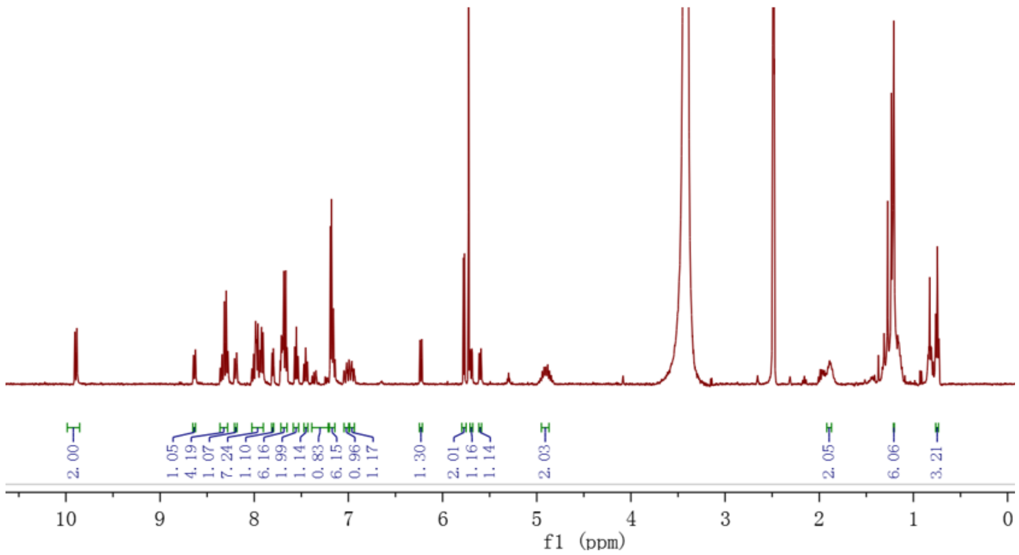
2.00
1.05
4.19
1.07
7.24
1.10
6.16
1.99
1.14
0.83
6.15
0.96
1.17
1.30
2.01
1.16
1.14
2.03
2.05
6.06
3.21
10
9
8
7
6
5
4
3
2
1
0
f1 (ppm)

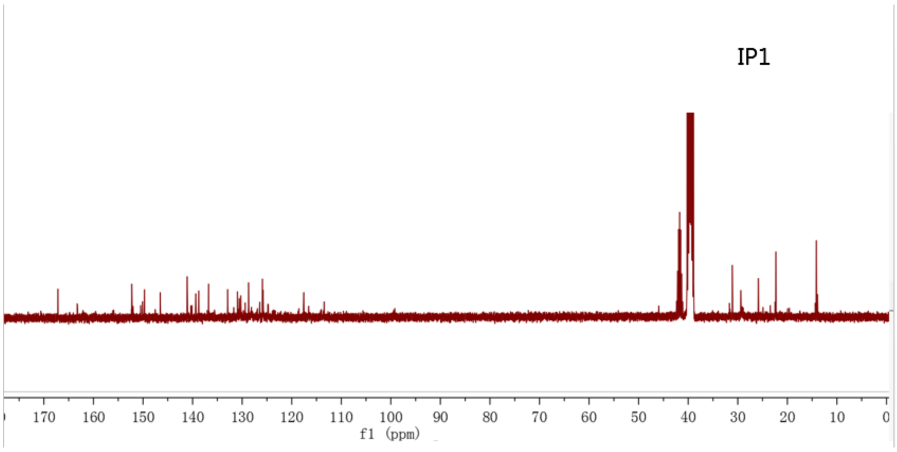

IP1
170
160
150
140
130
120
110
100
90
80
70
60
50
40
30
20
10
0
f1 (ppm)

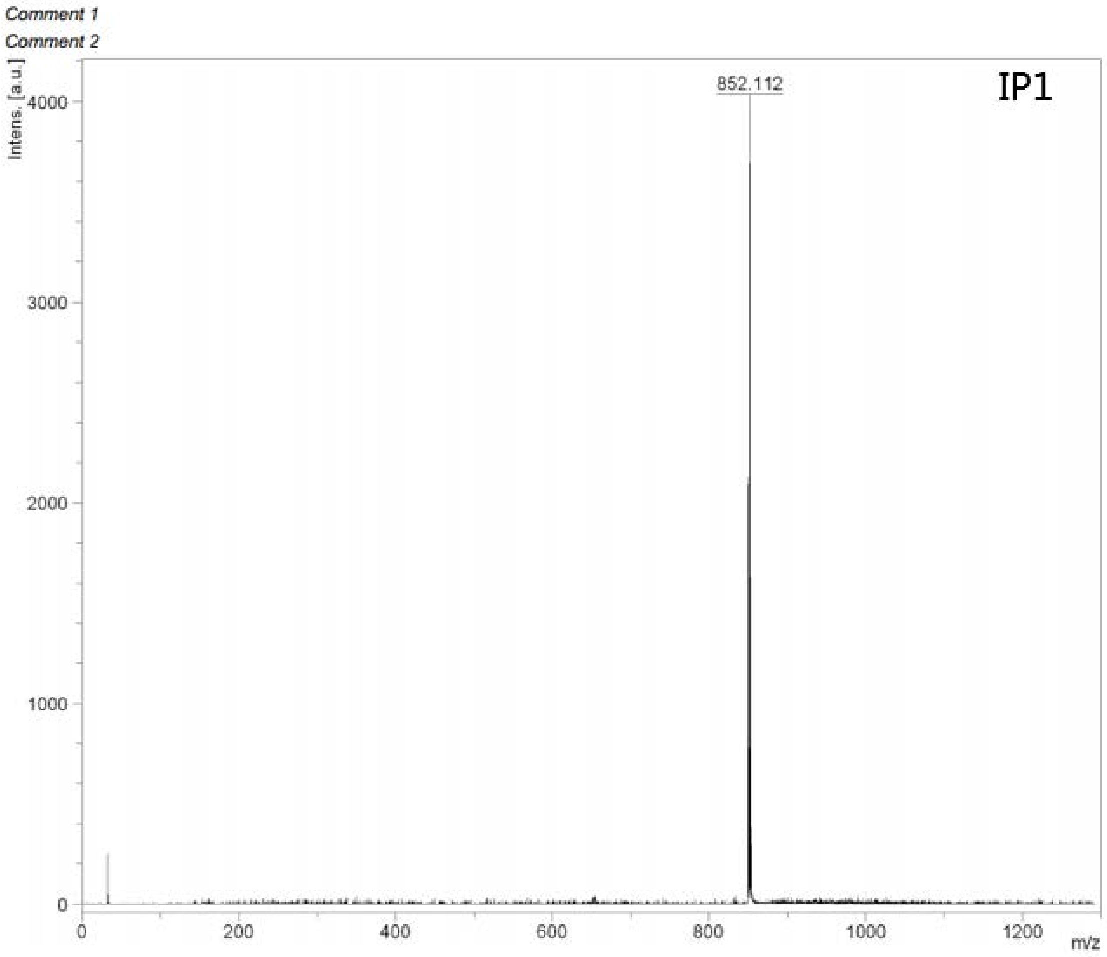
Comment 1
Comment 2
Intens. [a.u.]
852.112
IP1
4000
3000
2000
1000
0
0
200
400
600
800
1000
1200
m/z

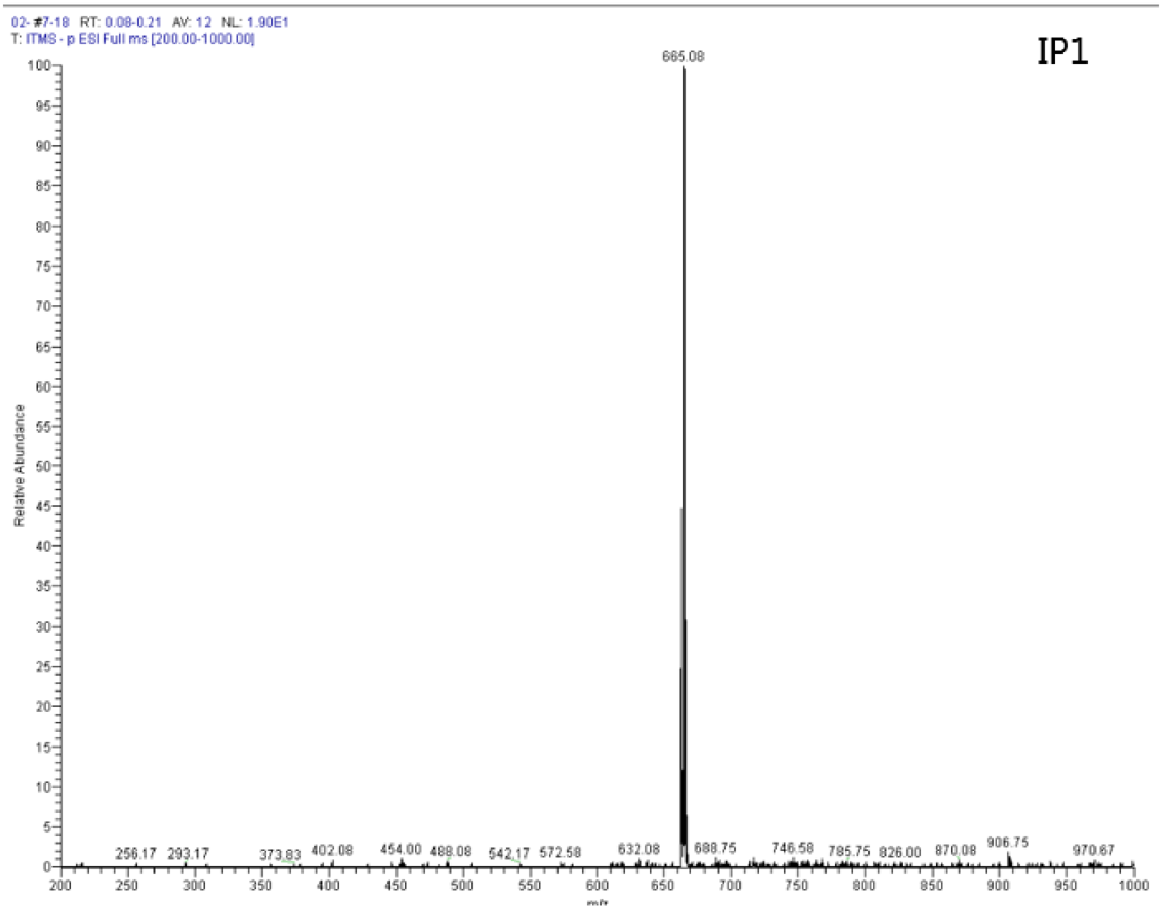
02- #7-18 RT: 0.08-0.21 AV: 12 NL: 1.90E1
T: ITMS - p ESI Full ms [200.00-1000.00]
IP1
665.08
Relative Abundance
256.17
293.17
373.83
402.08
454.00
488.08
542.17
572.58
632.08
688.75
746.58
785.75
826.00
870.08
906.75
970.67
m/z

IP2

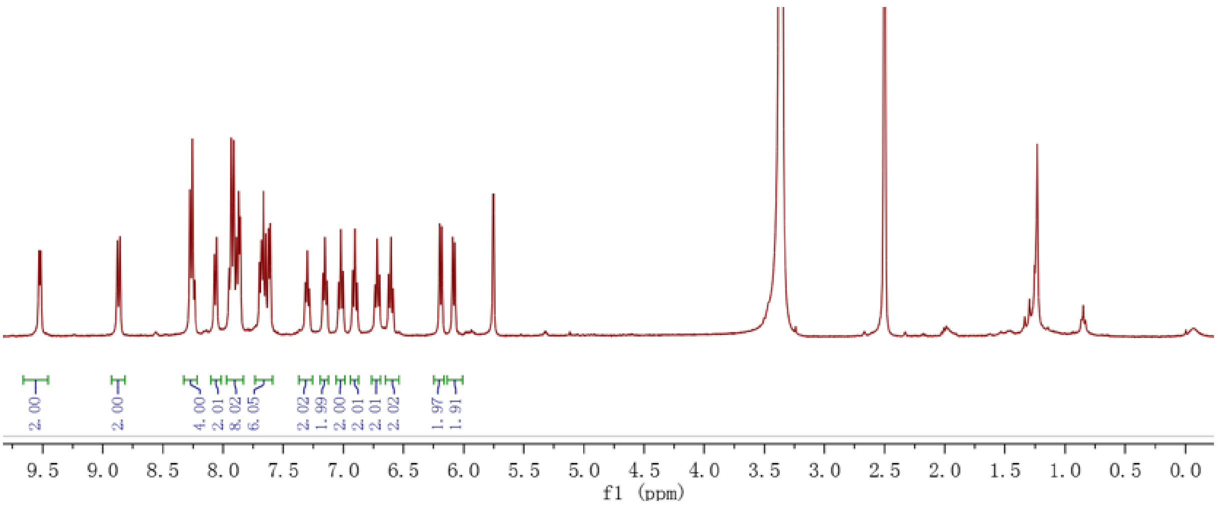
2.00
2.00
4.00
2.01
8.02
6.05
2.02
1.99
2.00
2.01
2.01
2.02
1.97
1.91
9.5 9.0 8.5 8.0 7.5 7.0 6.5 6.0 5.5 5.0 4.5 4.0 3.5 3.0 2.5 2.0 1.5 1.0 0.5 0.0
f1 (ppm)

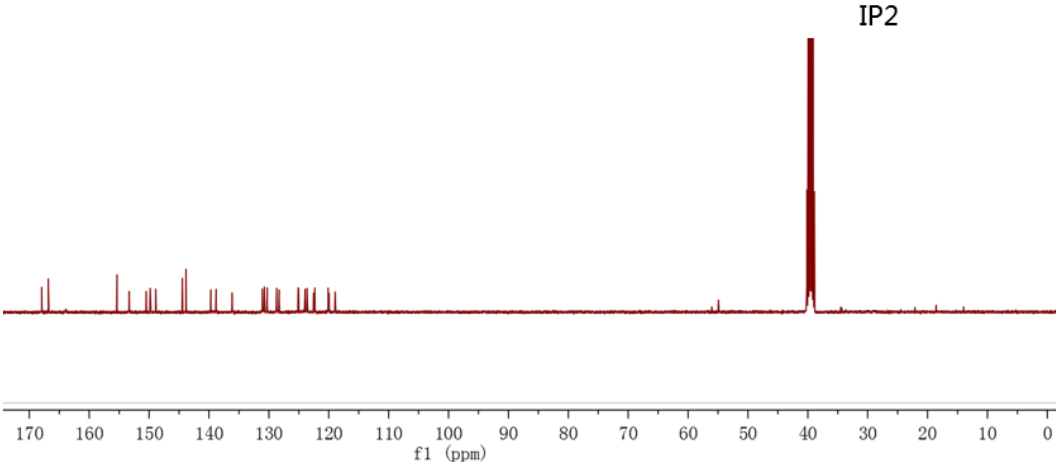
IP2
170 160 150 140 130 120 110 100 90 80 70 60 50 40 30 20 10 0
f1 (ppm)

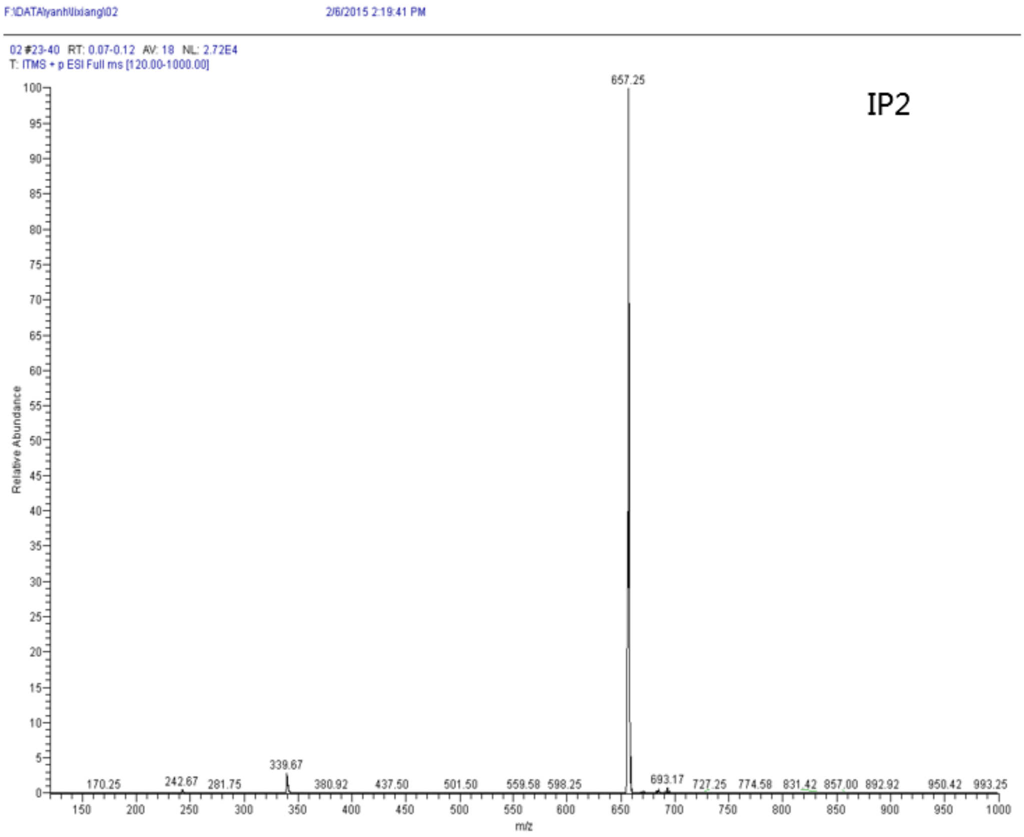
F:\DATA\yanhl\lixiang\02
2/6/2015 2:19:41 PM
02 #23-40 RT: 0.07-0.12 AV: 18 NL: 2.72E4
T: ITMS + p ESI Full ms [120.00-1000.00]
IP2
Relative Abundance
m/z
657.25
339.67
693.17
170.25
242.67
281.75
380.92
437.50
501.50
559.58
598.25
727.25
774.58
831.42
857.00
892.92
950.42
993.25

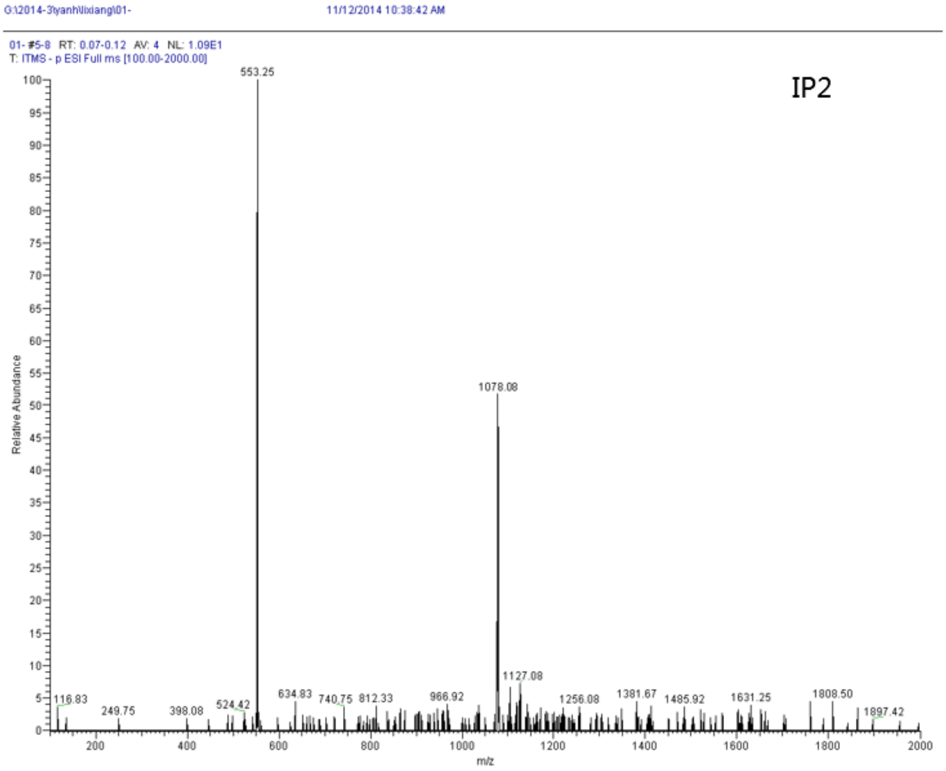

G:\2014-3\yanh\lixiang\01-
11/12/2014 10:38:42 AM
01- #5-8 RT: 0.07-0.12 AV: 4 NL: 1.09E1
T: ITMS - p ESI Full ms [100.00-2000.00]
IP2
553.25
1078.08
1127.08
116.83
249.75
398.08
524.42
634.83
740.75
812.33
966.92
1256.08
1381.67
1485.92
1631.25
1808.50
1897.42
Relative Abundance
m/z

IP3

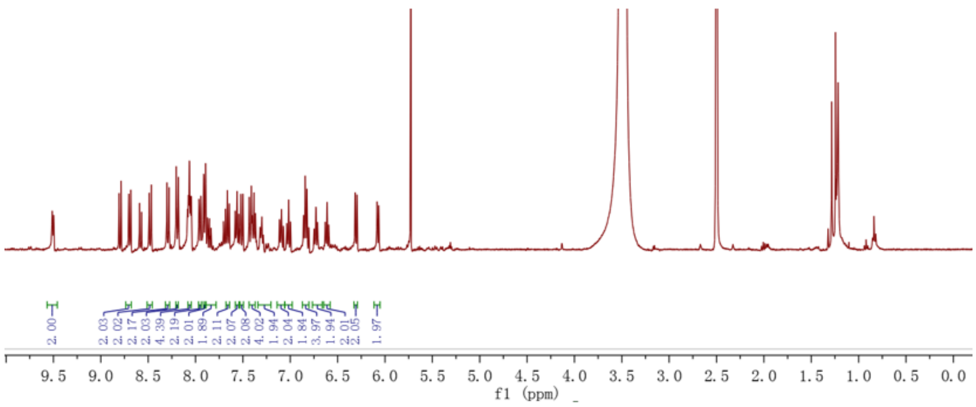

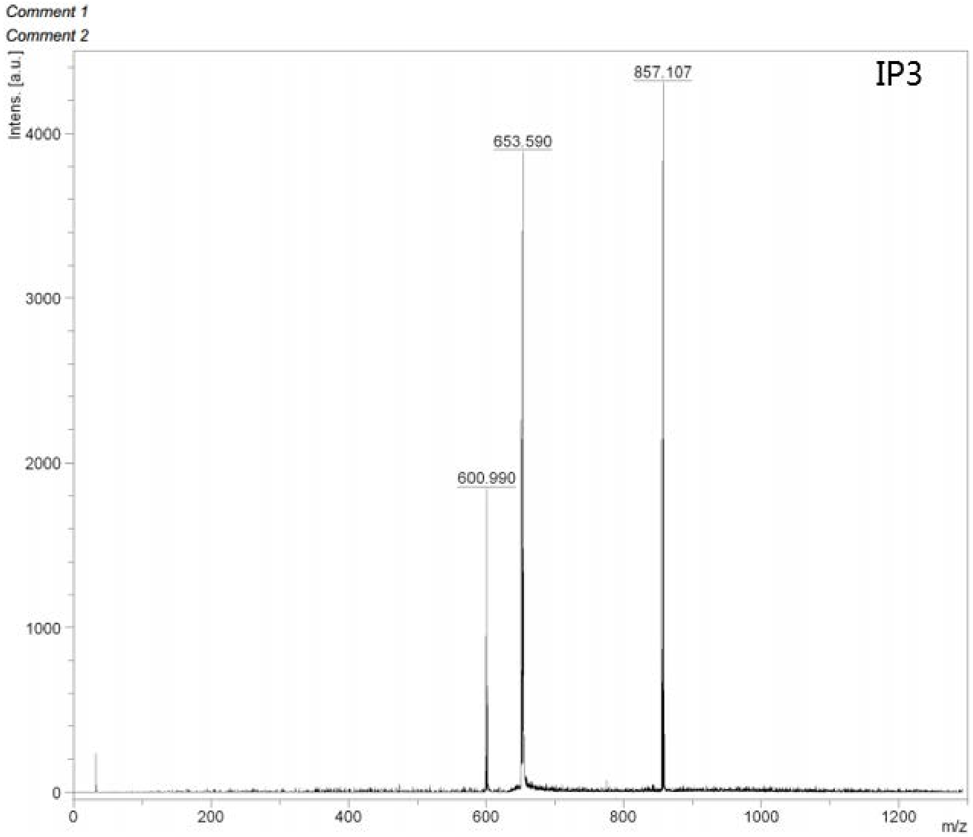
Comment 1
Comment 2
Intens. [a.u.]
IP3
857.107
653.590
600.990
4000
3000
2000
1000
0
0
200
400
600
800
1000
1200
m/z

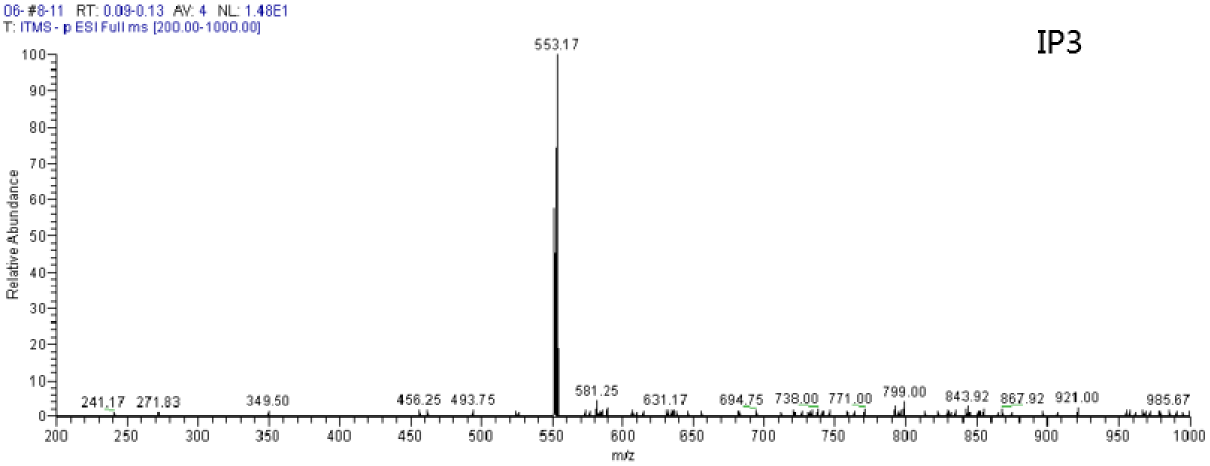
06- #8-11 RT: 0.09-0.13 AV: 4 NL: 1.48E1
T: ITMS - p ESI Full ms [200.00-1000.00]
IP3
553.17
Relative Abundance
m/z
241.17
271.83
349.50
456.25
493.75
581.25
631.17
694.75
738.00
771.00
799.00
843.92
867.92
921.00
985.67

IP4

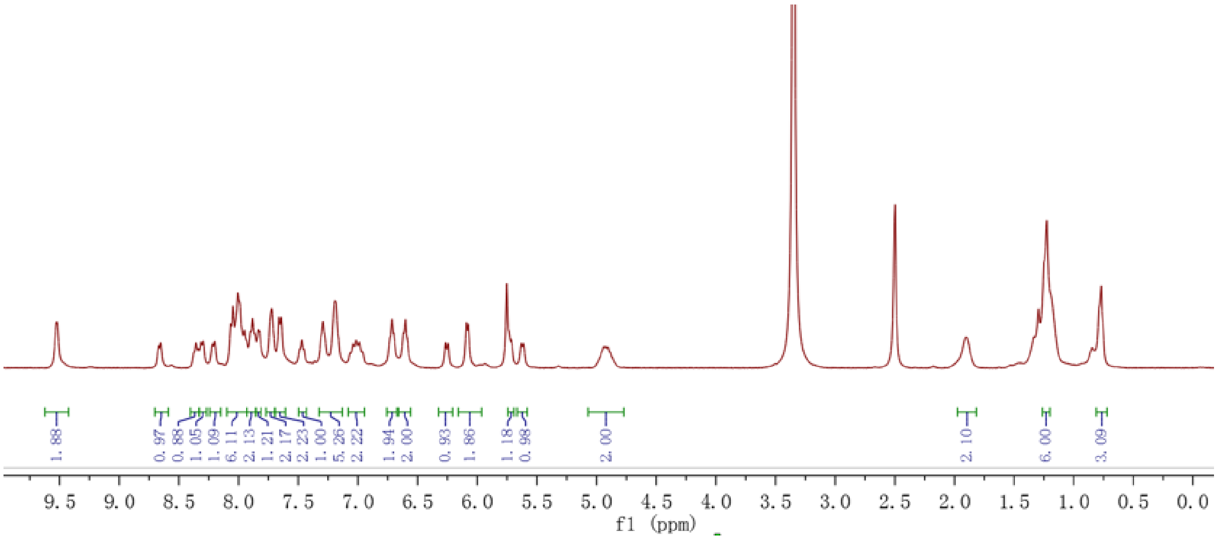

1.88
0.97
0.88
1.05
1.09
6.11
2.13
1.21
2.17
2.23
1.00
5.26
2.22
1.94
2.00
0.93
1.86
1.18
0.98
2.00
2.10
6.00
3.09
9.5
9.0
8.5
8.0
7.5
7.0
6.5
6.0
5.5
5.0
4.5
4.0
3.5
3.0
2.5
2.0
1.5
1.0
0.5
0.0
f1 (ppm)

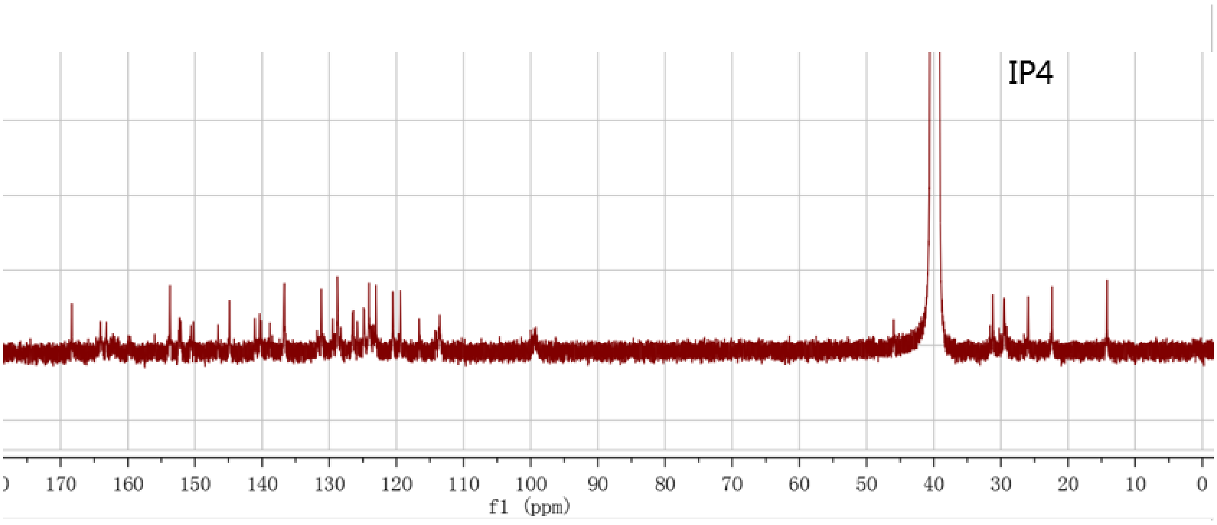
IP4
170 160 150 140 130 120 110 100 90 80 70 60 50 40 30 20 10 0
f1 (ppm)

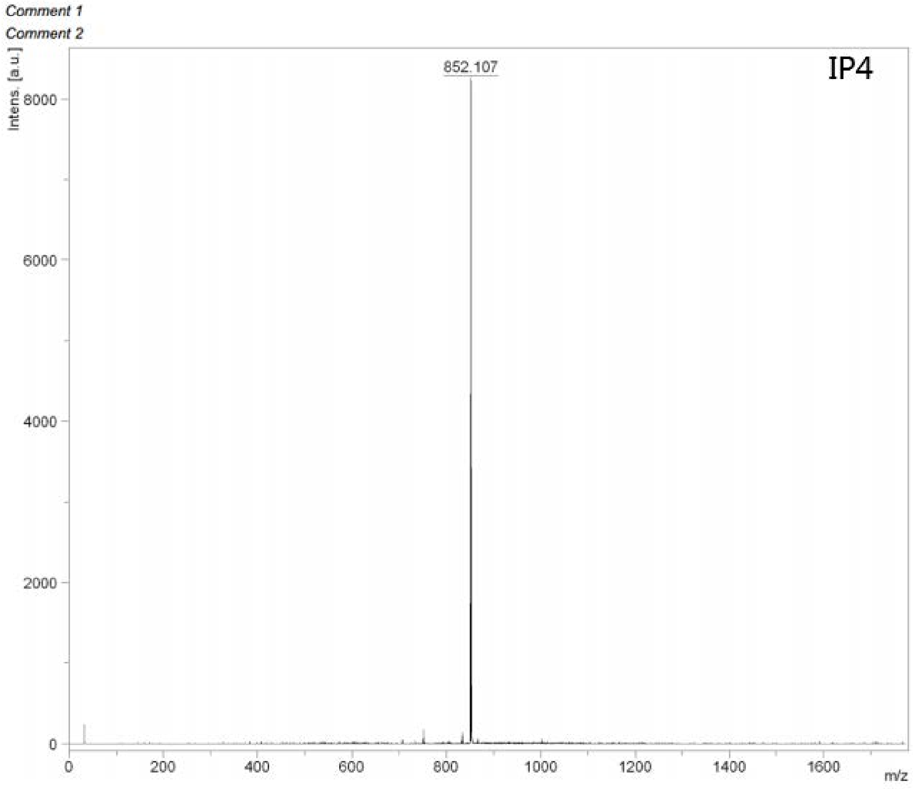
Comment 1
Comment 2
852.107
IP4
Intens. [a.u.]
8000
6000
4000
2000
0
0
200
400
600
800
1000
1200
1400
1600
m/z

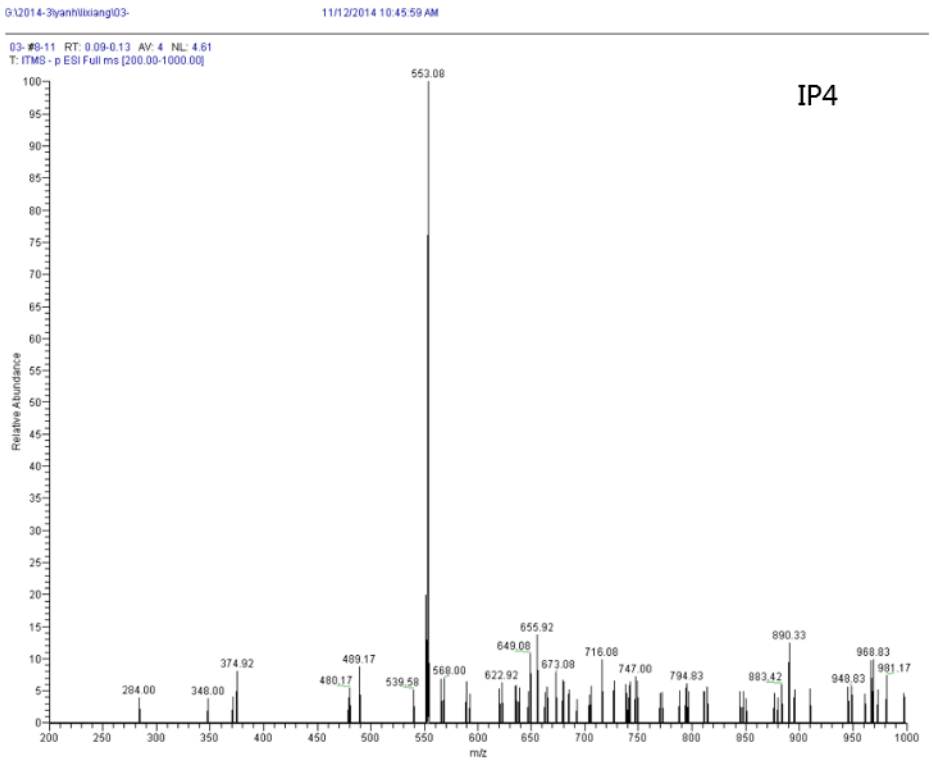
G:\2014-3\yanh\lixiang\03-
11/12/2014 10:45:59 AM
03- #8-11 RT: 0.09-0.13 AV: 4 NL: 4.61
T: ITMS - p ESI Full ms [200.00-1000.00]
IP4
Relative Abundance
m/z
553.08
655.92
890.33
649.08
716.08
968.83
489.17
673.08
747.00
981.17
374.92
568.00
622.92
794.83
883.42
948.83
480.17
539.58
284.00
348.00

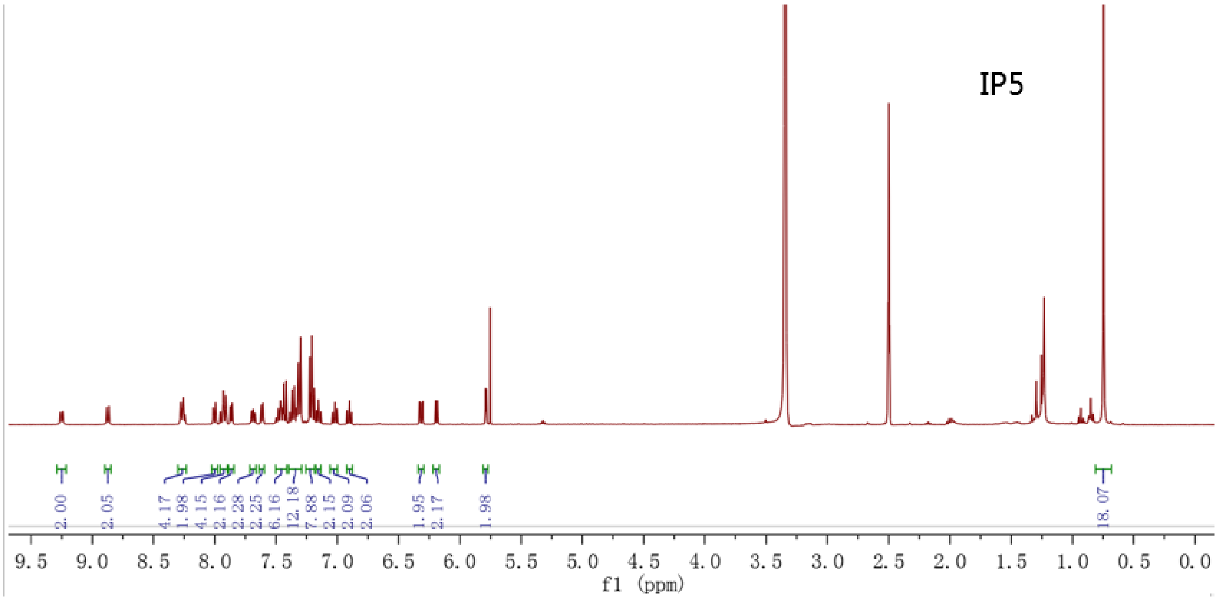
IP5
2.00
2.05
4.17
1.98
4.15
2.16
2.28
2.25
6.16
12.18
7.88
2.15
2.09
2.06
1.95
2.17
1.98
18.07
9.5 9.0 8.5 8.0 7.5 7.0 6.5 6.0 5.5 5.0 4.5 4.0 3.5 3.0 2.5 2.0 1.5 1.0 0.5 0.0
f1 (ppm)

IP5

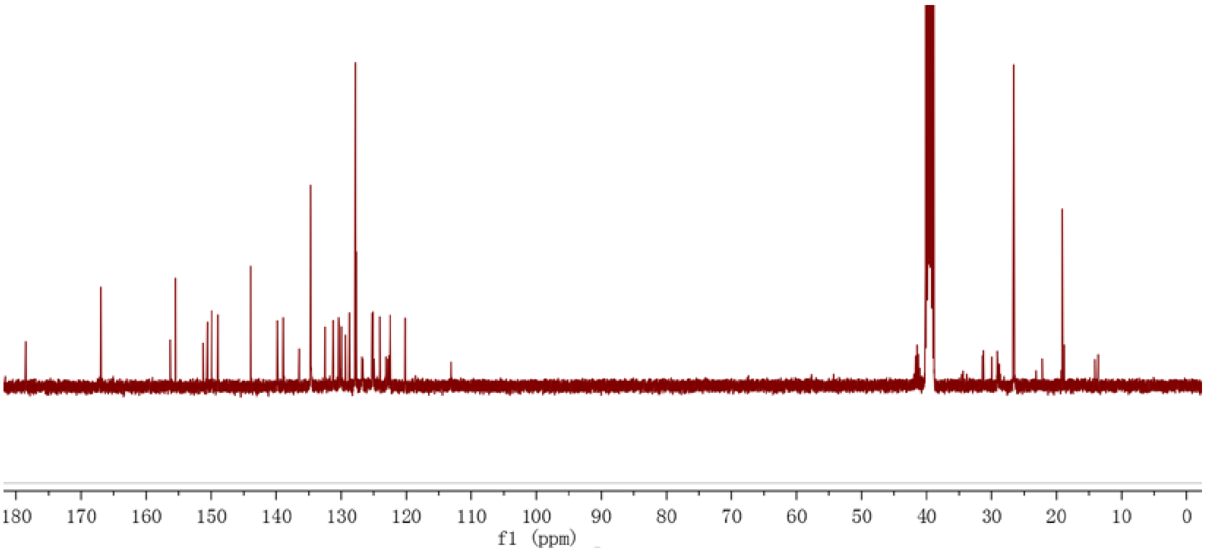

180
170
160
150
140
130
120
110
100
90
80
70
60
50
40
30
20
10
0
f1 (ppm)

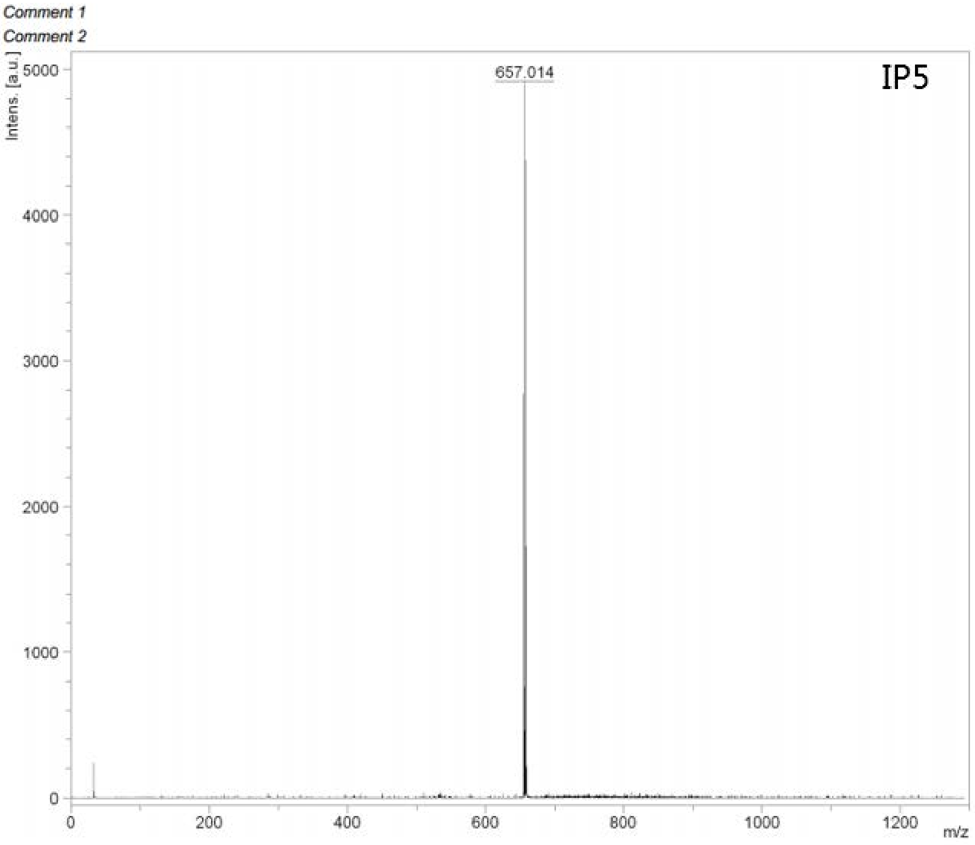
Comment 1
Comment 2
Intens. [a.u.]
5000
4000
3000
2000
1000
0
657.014
IP5
0
200
400
600
800
1000
1200
m/z

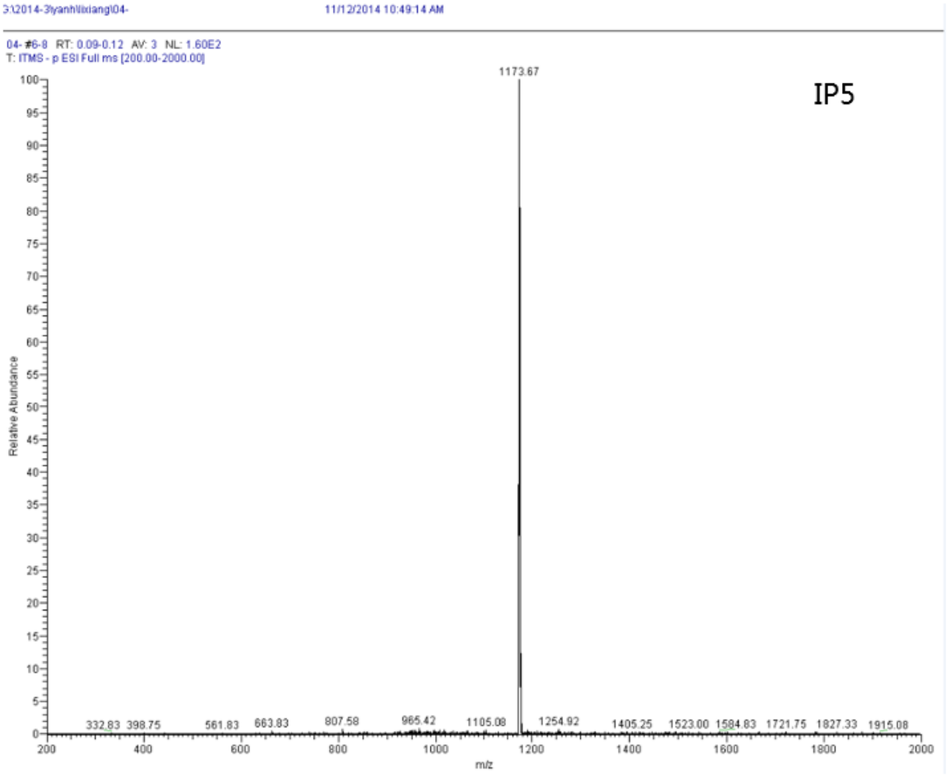

G:\2014-3\yanh\lixiang\04-
11/12/2014 10:49:14 AM
04- #6-8 RT: 0.09-0.12 AV: 3 NL: 1.60E2
T: ITMS - p ESI Full ms [200.00-2000.00]
IP5
1173.67
332.83 398.75 561.83 663.83 807.58 965.42 1105.08 1254.92 1405.25 1523.00 1584.83 1721.75 1827.33 1915.08
Relative Abundance
m/z

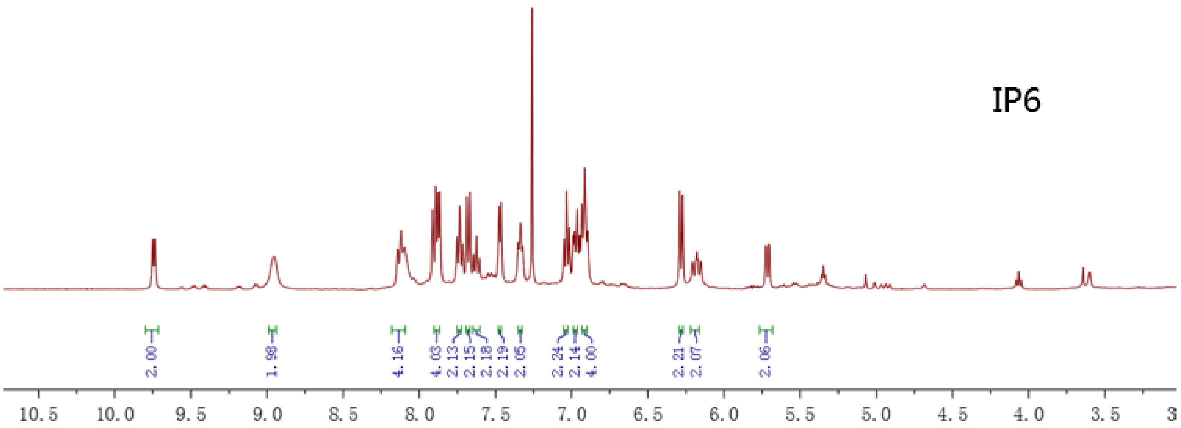
IP6
2.00
1.98
4.16
4.03
2.13
2.15
2.18
2.19
2.05
2.24
2.14
4.00
2.21
2.07
2.06
10.5
10.0
9.5
9.0
8.5
8.0
7.5
7.0
6.5
6.0
5.5
5.0
4.5
4.0
3.5
3

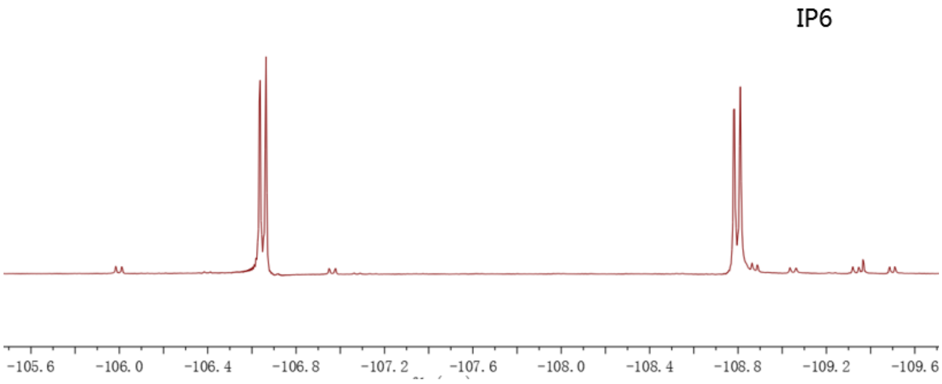

$^{19}F\{^1H\}$ NMR (377 M)

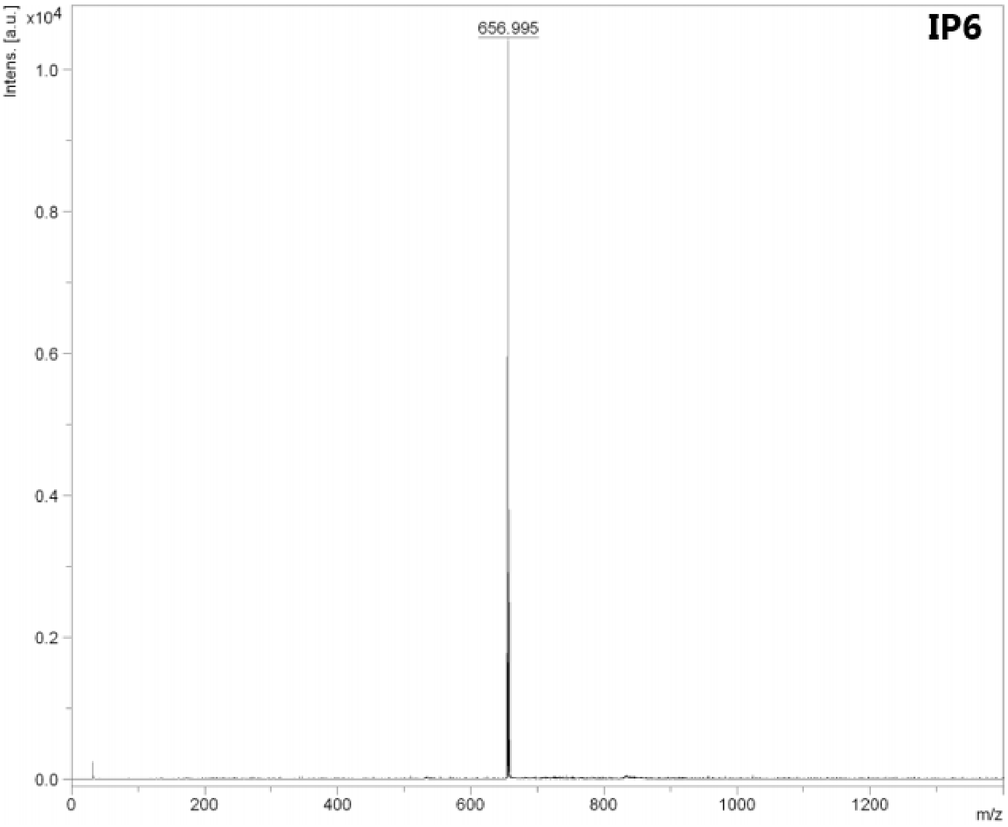
Intens. [a.u.]
x10^4
656.995
IP6
1.0
0.8
0.6
0.4
0.2
0.0
0
200
400
600
800
1000
1200
m/z

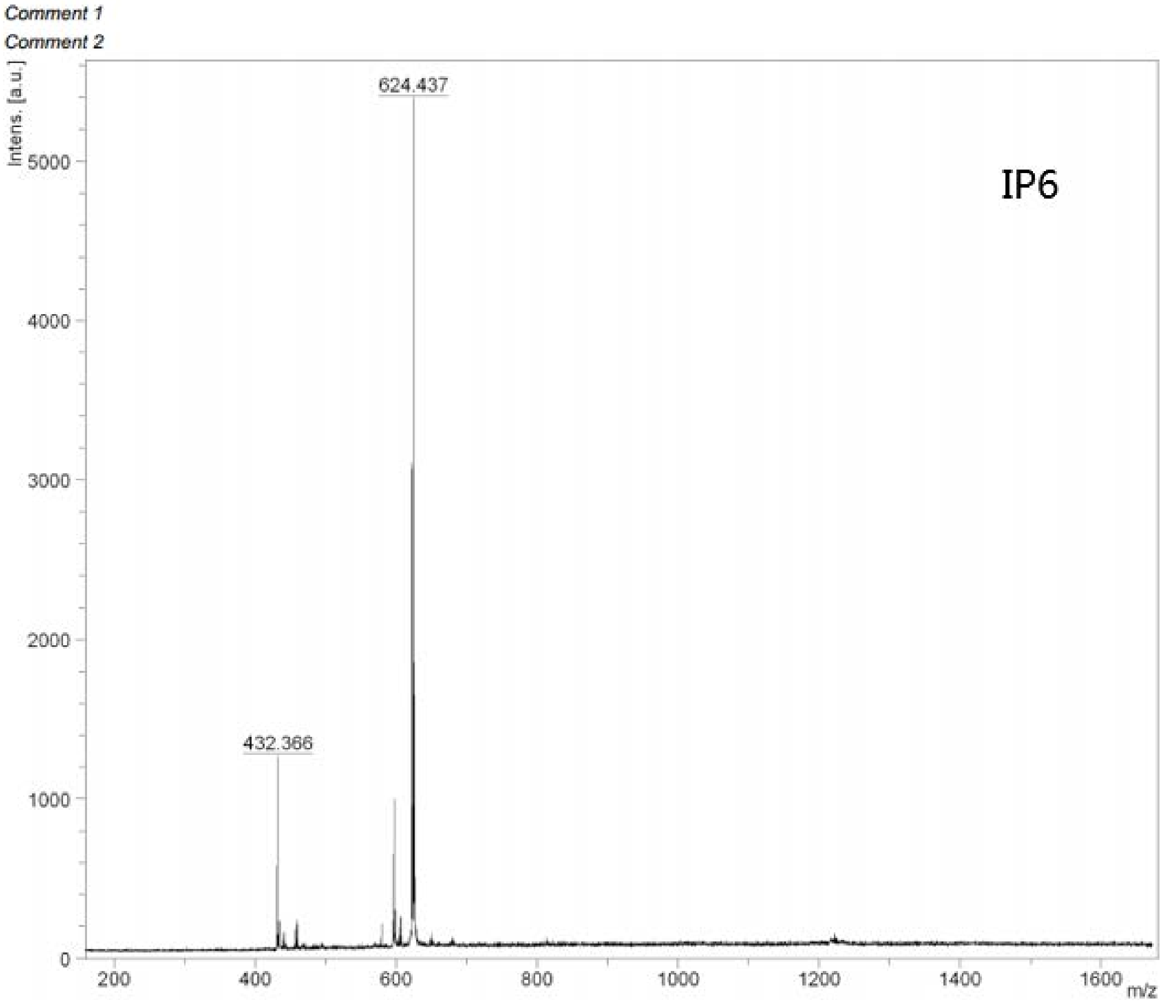
Comment 1
Comment 2
Intens. [a.u.]
624.437
432.366
IP6
5000
4000
3000
2000
1000
0
200
400
600
800
1000
1200
1400
1600
m/z
